# Tacrolimus or Mycophenolate Mofetil for Frequently Relapsing or Steroid-Dependent Nephrotic Syndrome: A Randomized Clinical Trial

**DOI:** 10.1001/jamapediatrics.2025.0765

**Published:** 2025-05-12

**Authors:** Jingjing Wang, Fei Liu, Weili Yan, Jianhua Zhou, Yu Zhang, Liping Rong, Xiaoyun Jiang, Fei Zhao, Chunhua Zhu, Xiaochuan Wu, Xiaoyan Li, Shuzhen Sun, Jing Wang, Mo Wang, Qin Yang, Hong Xu, Jing Chen, Cuihua Liu, Ming Tian, Shipin Feng, Qinwei Duan, Xuhui Zhong, Yun Zhu, Xiaozhong Li, Haidong Fu, Lingfei Huang, Daqing Ma, Jie Ding, Qing Ye, Jianhua Mao

**Affiliations:** 1 Department of Nephrology, Children’s Hospital, Zhejiang University School of Medicine, National Clinical Research Center for Child Health, Hangzhou, China; 2 Department of Clinical Epidemiology and Clinical Trial Unit, Children’s Hospital of Fudan University, National Children’s Medical Center, Shanghai, China; 3 Department of Pediatrics, Tongji Hospital, Tongji Medical College, Huazhong University of Science and Technology, Wuhan, China; 4 Department of Pediatric Nephrology and Rheumatology, The First Affiliated Hospital, Sun Yat-sen University, Guangzhou, China; 5 Department of Pediatric Nephrology, Children's Hospital of Nanjing Medical University, Nanjing, China; 6 Department of Pediatrics, The Second Xiangya Hospital of Central South University, Changsha, China; 7 Department of Pediatric Nephrology and Rheumatism and Immunology, Shandong Provincial Hospital Affiliated to Shandong First Medical University, Jinan, China; 8 Department of Nephrology, Children Hospital of Chongqing Medical University, National Clinical Research Center for Child Health and Disorders, Ministry of Education Key Laboratory, Chongqing, China; 9 Department of Nephrology, Children’s Hospital of Fudan University, Shanghai, China; 10 Department of Pediatric Nephrology and Rheumatology, Children’s Hospital Affiliated to Zhengzhou University, Henan Children’s Hospital, Zhengzhou Children’s Hospital, Zhengzhou, China; 11 The Affiliated Women’s and Children’s Hospital, School of Medicine, University of Electronic Science and Technology of China (UESTC), Chengdu, China; 12 Chengdu Women’s and Children’s Central Hospital, Chengdu, China; 13 Department of Pediatric Nephrology, Peking University First Hospital, Beijing, China; 14 Nephrology and Immunology Department of Children Hospital Affiliated to Soochow University, Suzhou, China; 15 Department of Pharmacy, Children’s Hospital, Zhejiang University School of Medicine, National Clinical Research Center for Child Health, Hangzhou, China; 16 Perioperative and Systems Medicine Laboratory, Department of Anesthesiology, Children’s Hospital, Zhejiang University School of Medicine, National Clinical Research Center for Child Health, Hangzhou, China; 17 Division of Anaesthetics, Pain Medicine and Intensive Care, Department of Surgery and Cancer, Faculty of Medicine, Chelsea and Westminster Hospital, Imperial College London, London, United Kingdom; 18 Department of Laboratory Medicine, Children Hospital, Zhejiang University School of Medicine, National Clinical Research Center for Child Health, Hangzhou, China

## Abstract

**Importance:**

Both tacrolimus (TAC) and mycophenolate mofetil (MMF) are recommended for children with frequently relapsing nephrotic syndrome (FRNS) or steroid-dependent nephrotic syndrome (SDNS). However, their comparative effectiveness and safety have not been evaluated through randomized clinical trials.

**Objective:**

To compare the effectiveness and safety of TAC and MMF in children with FRNS or SDNS.

**Design, Setting, and Participants:**

In this multicenter, open-label randomized clinical trial conducted at 12 pediatric nephrology centers across China, 270 children aged 2 to 18 years with FRNS or SDNS were allocated at a 1:1 ratio to treatment with either TAC or MMF. The study was conducted from November 2019 to July 2023, and data analysis was completed from July 2023 to March 2024.

**Intervention:**

Patients received either TAC (0.025-0.050 mg/kg, orally twice daily) or MMF (10-15 mg/kg, orally twice daily) for 1 year, along with a tapering regimen of steroids.

**Main Outcomes and Measures:**

The primary end point was 1-year relapse-free survival. Relapse frequency, cumulative steroid dosage, and safety profiles were also evaluated.

**Results:**

A total of 292 patients from 12 care centers were assessed for eligibility, and 270 patients were randomized to receive either TAC (n = 135) or MMF (n = 135). Among 270 patients, median (IQR) age was 6.91 (4.25-9.96) years, and 70 patients (25.9%) were female. Compared with MMF, the 1-year relapse-free survival rate in the TAC group was 1.86-fold higher (hazard ratio [HR], 2.86; 95% CI, 1.79-4.76; *P* < .001) in the intention-to-treat analysis. This difference was also significant after adjusting for the per-protocol analysis (HR, 2.78; 95% CI, 1.72-4.55; *P* < .001). The mean (SD) time to first relapse was significantly longer in the TAC group (323.99 [98.33] days) compared to the MMF group (263.21 [132.84] days). Furthermore, the TAC group showed a lower annual relapse rate than the MMF group (17.78% vs 41.48%) and required a significantly lower mean (SD) cumulative steroid dose (0.22 [0.10] mg/kg/day vs 0.34 [0.22] mg/kg/day). The safety profile was similar in both groups.

**Conclusions and Relevance:**

In this randomized clinical trial, compared with MMF, a 1-year course of TAC therapy significantly extended the period of relapse-free survival in children with FRNS or SDNS.

**Trial Registration:**

ClinicalTrials.gov Identifier: NCT04048161

## Key Points

**Question:**

Despite both tacrolimus (TAC) and mycophenolate mofetil (MMF) being recommended for children with steroid-sensitive but frequently relapsing nephrotic syndrome (FRNS) or steroid-dependent nephrotic syndrome (SDNS), their comparative efficacy and safety have not been evaluated through randomized clinical trials.

**Findings:**

This multicenter, open-label, parallel-arm randomized clinical trial evaluates the efficacy and safety of TAC and MMF in the treatment of pediatric FRNS or SDNS. The results show that TAC extends relapse-free survival more than MMF, with similar safety.

**Meaning:**

This study suggests that TAC is superior for children with FRNS or SDNS.

## Teaser

This randomized clinical trial conducted at 12 pediatric nephrology centers in China compares the effectiveness and safety of tacrolimus and mycophenolate mofetil in children with frequently relapsing nephrotic syndrome or steroid-dependent nephrotic syndrome.

## Introduction

Idiopathic nephrotic syndrome, which is characterized by significant proteinuria, hypoalbuminemia, edema, and hyperlipidemia, is the most prevalent glomerular disease in children, with an estimated incidence of 3 cases per 100 000 children annually.^1^ For more than half a century, steroids have been used as the main therapy for this disease.^2^ Most patients (85%-90%) who have steroid-sensitive nephrotic syndrome achieve complete remission of proteinuria within 4 to 6 weeks of treatment with glucocorticoids^3^; unfortunately, approximately half of these patients develop frequently relapsing nephrotic syndrome (FRNS) or steroid-dependent nephrotic syndrome (SDNS).^1,4,5^ Therefore, FRNS and SDNS pose significant challenges because of the necessity of monitoring both disease complications and the adverse effects of treatment.

Recently, the International Pediatric Nephrology Association (IPNA) guidelines recommended the introduction of one of the following steroid-sparing agents (in alphabetical order) as the first-line therapy for children with FRNS or SDNS^3^: calcineurin inhibitors; cyclophosphamide; levamisole; and mycophenolate mofetil (MMF) or mycophenolic sodium (grade A, strong recommendation). However, the guidelines do not recommend which steroid-sparing agents should be prioritized for use,^3,6^ and there is insufficient evidence to establish the optimal choice of these agents. Previous uncontrolled studies have indicated that tacrolimus (TAC) may be as effective as MMF.^7,8^ Therefore, we designed this prospective, multicenter, open-label, parallel-arm randomized clinical trial to assess the comparative efficacy and safety of TAC and MMF in pediatric patients with FRNS or SDNS.

## Methods

### Study Design

This study of TAC vs MMF in pediatric patients with FRNS or SDNS (the STAMP trial) was a prospective, multicenter, open-label, parallel-arm randomized clinical trial. The study protocol and statistical analysis plan are available in Supplement 1 and Supplement 2, respectively. The reporting of this study adhered to the Consolidated Standards of Reporting Trials (CONSORT) reporting guidelines.^9^

### Participants

Patients aged 2 to 18 years with FRNS or SDNS were consecutively screened for eligibility and recruitment from 12 pediatric nephrology centers across China. The standard definitions^6,10^ used for the onset, remission, and relapse of nephrotic syndrome are delineated in Table 1. The inclusion criteria encompassed an estimated glomerular filtration rate (eGFR) greater than 90 mL/min/1.73 m^2^, a disease remission period, and no use of TAC, MMF, cyclosporine A, rituximab, or cyclophosphamide within the 2-year period prior to enrollment. The exclusion criteria were a secondary form of nephrotic syndrome and active infection. The comprehensive inclusion and exclusion criteria are elaborated in the study protocol (Supplement 1).

**Table 1.** Definitions^a^

Item	Definitions
Steroid-dependent nephrotic syndrome	2 Consecutive relapses during therapy with prednisone or prednisolone (either at full dose or during tapering) or within 15 d of prednisone or prednisolone discontinuation
Frequently relapsing nephrotic syndrome	≥2 Relapses per 6 mo within 6 mo of disease onset or ≥4 relapses per 12 mo in any subsequent 12-mo period
Relapse	For 3 consecutive days, morning urinary protein changes from negative to (+++) or (++++), 24-h urinary protein quantification ≥50 mg/kg, or urinary protein/creatinine ratio (mg/mg) ≥2.0
Remission	UPCR (based on first morning void or 24-h urine sample) ≤20 mg/mmol (0.2 mg/mg), <100 mg/m^2^ per day, or negative or trace dipstick on ≥3 consecutive days

Abbreviation: UPCR, urine protein to creatinine ratio.

^a^ All definitions are derived from references.^6,10^

This study received approval from the ethics committee of the Children’s Hospital, Zhejiang University School of Medicine (approval: 2019-IEC-003). After presenting a comprehensive written description of the study encompassing potential benefits, as well as a thorough assessment of study safety and risks to the patients, written informed consent was obtained from the parents of all patients. Additionally, for patients older than 8 years, patient assent was also secured.

### Randomization With No Masking

Eligible children were randomized 1:1 to receive TAC or MMF along with prednisolone over a 12-month period. Computer-generated random codes were used and the random envelopes were prepared accordingly. Each envelope was associated with a specific treatment group. To ensure allocation concealment, sequentially numbered, opaque, sealed envelopes were used. These envelopes were opened only after participants were enrolled to disclose the group assignment. Once a random number was used, if it became invalid during the process, it could not be reused. The trial was open-label, meaning that neither the patients nor the study staff were blinded to the treatment allocation.

### Procedures

Eligible patients were randomized 1:1 to receive oral TAC or MMF within a week postrandomization, with prednisolone tapering over a year (Supplement 1). Monthly blood samples were collected premedication for drug concentration measurements. TAC was administered at 0.025 to 0.050 mg/kg twice daily.^10^ The target blood concentration was 5 to 10 ng/mL for 6 months, after which it was maintained at less than 5 ng/mL.^10^ MMF was administered at a dose of 10 to 15 mg/kg twice daily. The target for mycophenolic acid area under the curve (MPA-AUC) was 30 to 50 μg·h/mL for 6 months, and subsequently maintained at 40 or less μg·h/mL (eMethods 1 in Supplement 3).^7,10^ Patients were monitored for 1 year, with visits at weeks 1, 4, and 8, then every 8 weeks until 40 weeks, with a final visit at 52 weeks. The following factors were monitored: relapses, corticosteroid dose, adverse events (AEs), tacrolimus level, MPA-AUC, blood liver or kidney function, lipids, and urine. Medication was dispensed at visits, with vials returned for counting. Participants and their caregivers kept daily diaries for medication and proteinuria. Treatment was stopped for withdrawal criteria (Supplement 1), including lack of efficacy.

### Outcomes

The primary outcome was 1-year relapse-free survival. The secondary outcomes included relapse frequency, the first relapse time, the relapse-free survival rate in the first 6 months, cumulative steroid dosage, blood pressure, height, body weight, blood cholesterol, serum albumin concentration, eGFR, and other kidney function indicators. Treatment safety was also assessed with respect to vital signs (respiration and heart rate), liver function, blood glucose, and adverse reactions. All AEs were coded with the Medical Dictionary for Regulatory Activities (MedDRA) version 26.0 to be presented as the total number and incidence of AEs. Participants were censored at their last follow-up or upon withdrawal or death if no clinical event had occurred.

### Statistical Analysis

Sample size was calculated based on a prior study showing 1-year relapse-free survival rates of 56% for the MMF group and 70% for the TAC group. With α = .05 (2-sided), 80% power, and 1:1 enrollment, PASS version 16 software (NCSS Statistical Software) estimated 114 patients prescribed MMF and 115 prescribed TAC (229 total). Accounting for 15% loss to follow-up, 270 patients (135 per group) were enrolled. Schoenfeld residuals assessed proportional hazards. Demographic and baseline characteristics were compared using *t* tests, Wilcoxon tests, χ^2^ tests, or Fisher exact tests. Comparisons between groups were made using the χ^2^ test or Fisher exact test, with *P* values only reported. Intention-to-treat analysis was followed, with censored data for incomplete visits. The log-rank test analyzed relapse-free survival, with Cox models calculating hazard ratios (HRs) and 95% confidence intevals. Sensitivity analysis used per-protocol set (PPS) data, adjusting for covariates. Kaplan-Meier curves plotted 1-year survival rates. Mixed models for repeated measures assessed eGFR changes, reporting least-square means, least-square mean differences, and 95% confidence intervals. Annualized eGFR slopes were compared using a linear mixed-effects model. Safety analyses used the safety set, with AEs coded by MedDRA version 26.0. The detailed statistical analysis methods are outlined in the eMethods 2 in Supplement 3 and the statistical analysis plan provided in Supplement 2.

## Results

### Patient Characteristics

**Figure 1.**
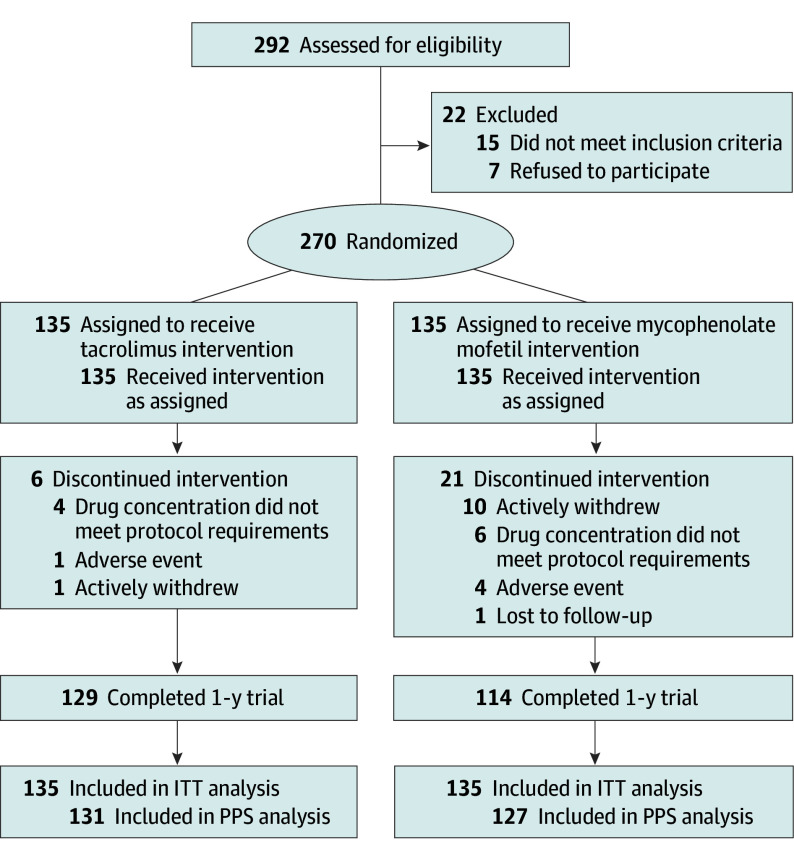
Trial Flow ITT indicates intention to treat; PPS, per-protocol set.

A total of 292 patients from 12 care centers were assessed for eligibility from November 2019 to May 2022, and 270 patients were randomized to receive either TAC (n = 135) or MMF (n = 135). Among 270 patients, median (IQR) age was 6.91 (4.25-9.96) years, and 70 patients (25.9%) were female. Study follow-up ended in July 2023. A total of 243 patients completed a 1-year trial, with 27 dropouts (6 from the TAC group and 21 from the MMF group). The total dropout rate of 10% in the MMF group (27 of 270 total patients) was due to active withdrawal (10 patients), inability to achieve the target drug concentration even after 1 month of dose adjustment (6 patients), AEs (4 patients), and loss to follow-up (1 patient), whereas in the TAC group, dropout was due to active withdrawal (1 patient), the inability to achieve the target drug concentration even after 1 month of dose adjustment (4 patients), and AEs (1 patient) (Figure 1; eTable 1 in Supplement 3). Similar baseline characteristics were observed between treatment groups not only in the initial cohort, but also in the patients who completed the study (Table 2; eTable 2 in Supplement 3).^11,12^ Diary reviews and returned pill counts suggested good treatment adherence in both groups.

**Table 2.** Characteristics of Patients at Baseline

Characteristic	Mean (SD)	
	TAC (n = 135)	MMF (n = 135)
Age of enrollment, median (range), y	6.86 (2.16 to 16.43)	7.00 (1.95 to 16.53)
Sex, No. (%)		
Female	34 (25.19)	36 (26.67)
Male	101 (74.81)	99 (73.33)
Height, cm	118.77 (20.97)	119.91 (21.11)
Height for age *z* score	−0.71 (1.25)	−0.46 (1.08)
Weight, kg	27.08 (12.31)	28.40 (15.30)
Weight for age *z* score	0.43 (1.08)	0.46 (1.17)
BMI^a^	18.33 (3.59)	18.42 (3.61)
BMI for age *z* score	1.05 (1.61)	1.04 (1.29)
Systolic blood pressure, mm Hg	104.90 (11.44)	104.87 (10.40)
Systolic blood pressure *z* score^b^	1.62 (0.87)	1.57 (0.78)
Diastolic blood pressure, mm Hg	65.82 (9.83)	65.54 (9.46)
Diastolic blood pressure *z* score^b^	0.98 (1.09)	0.92 (1.15)
eGFR, ml/min/1.73 m^2^^c^	164.81 (35.71)	165.54 (39.46)
Serum creatinine, mg/dL	0.41 (0.12)	0.41 (0.14)
Total cholesterol, mg/dL	253.67 (89.96)	242.86 (74.90)
Albumin, g/dL	3.42 (0.72)	3.47 (0.72)
Total protein, g/dL	5.80 (0.79)	5.83 (0.84)
Fasting blood glucose, mg/dL	78.20 (12.97)	79.82 (14.95)
Duration of disease, median (IQR), y	1.40 (0.75 to 3.00)	1.13 (0.70 to 2.40)
No. of relapses in the prestudy half year	2.16 (0.74)	2.27 (1.10)

Abbreviations: BMI, body mass index; eGFR, estimated glomerular filtration rate; MMF, mycophenolate mofetil; TAC, tacrolimus.

SI conversion factors: To convert serum creatinine from mg/dL to μmol/L, multiply by 88.4; total cholesterol, from mg/dL to mmol/L, multiply by 0.0259; albumin, from g/dL to g/L, multiply by 10; total protein, from g/dL to g/L, multiply by 10; blood glucose, from mg/dL to mmol/L, multiply by 0.0555.

^a^ Calculated as weight in kilograms divided by height in meters squared.

^b^ *z* Scores for systolic and diastolic blood pressure were derived based on published data.^11^

^c^ eGFR (calculated by the Schwartz formula^12^), height for age *z* scores, weight for age *z* scores, and BMI for age *z* scores were derived based on data published by the World Health Organization.

### Efficacy

**Figure 2.**
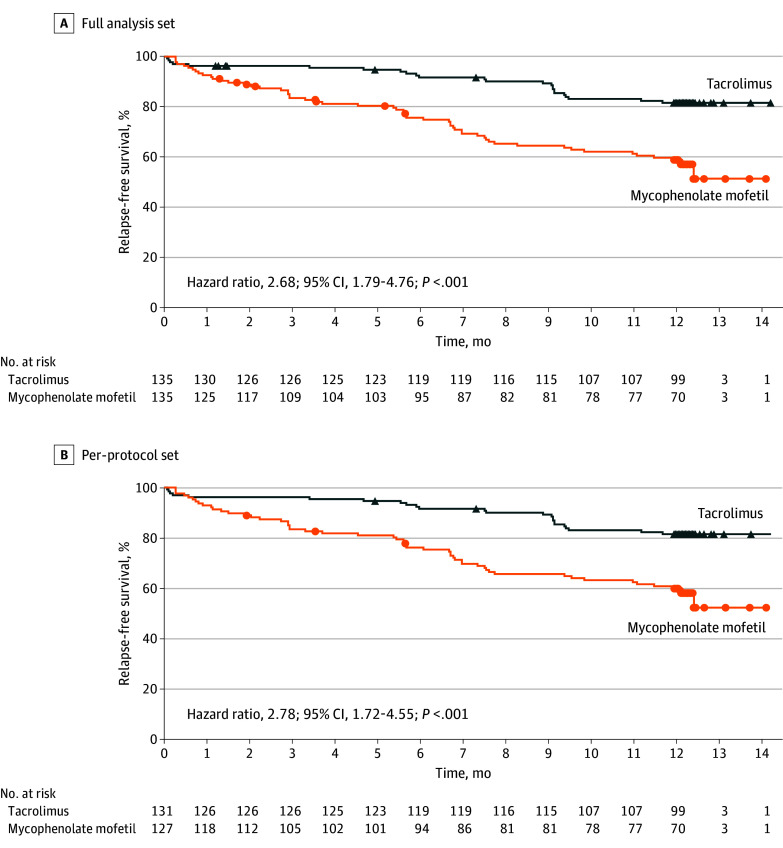
One-Year Relapse-Free Survival Kaplan-Meier curves for relapse-free survival rates in the tacrolimus and mycophenolate mofetil groups. A, In the full analysis set, compared with mycophenolate mofetil, the 1-year relapse-free survival rate in the tacrolimus group was 1.86-fold higher. B, In the per-protocol set, compared with mycophenolate mofetil, the 1-year relapse-free survival rate in the tacrolimus group was 1.78-fold higher.

Schoenfeld residual tests confirmed that the proportional hazards assumption was satisfied for all covariates. Compared with MMF, TAC was superior for treating children with FRNS or SDNS. In the full analysis set (FAS), the 1-year relapse-free survival rate in the TAC group was 1.86-fold higher (HR, 2.86; 95% CI, 1.79-4.76; *P* < .001; Figure 2A, Table 3). In line with these results, the PPS analysis also revealed that the 1-year relapse-free survival rate in the TAC-treated group increased by 1.78-fold (HR, 2.78; 95% CI, 1.72-4.55; *P* < .001; Figure 2B). The TAC group showed significantly lower relapse rates (17.78% in FAS and 18.32% in PPS) compared to the MMF group (41.48% in FAS and 41.73% in PPS). Log-rank tests yielded *P* values less than .001, indicating significant survival differences (Table 3). Unadjusted and adjusted Cox regression analyses consistently demonstrated HRs of 0.35 to 0.36 for TAC vs MMF (95% CI, 0.21-0.59; *P* < .001; eTable 3 in Supplement 3). During the 1-year treatment, 24 patients experienced relapse, and a total of 43 relapse events were recorded among the 135 patients in the TAC group; however, in the MMF group, 56 patients experienced relapse, and 105 relapse events were noted among the 135 patients. The mean (SD) time to first relapse was significantly longer in the TAC group (323.99 [98.33] days) compared to the MMF group (263.21 [132.84] days; Table 3). Furthermore, the subgroup analysis revealed the TAC group had a lower incidence of relapse than the MMF group in both the FAS and PPS. For toddler-aged participants (<4 years), the relapse rates were 17.24% vs 51.72% (FAS: HR, 0.15; 95% CI, 0.05-0.45; PPS: HR, 0.09; 95% CI, 0.03-0.33). For patients of preschool age (4-<7 years), the rates were 12.20% vs 47.37% (FAS: HR, 0.14; 95% CI, 0.05-0.40; PPS: HR, 0.14; 95% CI, 0.05-0.41). For school-aged and older patients (≥7 years), the rates were 21.54% vs 33.82% (FAS: HR, 0.52; 95% CI, 0.26-1.05; PPS: HR, 0.55; 95% CI, 0.27-1.11; eFigure in Supplement 3).

**Table 3.** Primary End Point and Secondary End Points

End point	Group		Between-group difference (95% CI)^a^	*P* value
	TAC	MMF		
Primary end point, No.	135	135	NA	NA
1-y Relapse-free survival, No. (%)	111 (82.22)	79 (58.52)	2.86 (1.79 to 4.76)	<.001
Secondary end points				
Time to first relapse after enrollment, mean (SD), d	323.99 (98.33)	263.21 (132.84)	60.79 (32.77 to 88.80)	<.001
Cumulative incidence of relapses within 1 y, No. (%)	24 (17.78)	56 (41.48)	−0.24 (−0.34 to −0.13)	<.001
Frequency of relapses, No. (%)				
0	111 (82.22)	79 (58.52)	NA	NA
Approximately 1-3	19 (14.07)	50 (37.04)	NA	NA
≥4	5 (3.70)	6 (4.44)	NA	NA
No. of patients who relapsed within 6 mo, No. (%)	8 (5.93)	32 (23.70)	4.55 (2.08 to 10.00)	<.001
Cumulative prednisolone dose in study year, mean (SD), mg/kg/d	0.22 (0.10	0.34 (0.22)	−0.12 (−0.16 to −0.07)	<.001
Change in HAZ from baseline at 1 y	0.09 (0.86	0.00 (0.44)	0.09 (−0.08 to 0.26)	.44
Change in WHZ from baseline at 1 y	−0.22 (0.88	−0.21 (0.75)	−0.00 (−0.26 to 0.25)	.84
Change in BMI *z* score from baseline at 1 y^b^	−0.45 (1.55	−0.37 (1.11)	−0.08 (−0.42 to 0.26)	.96
Change in SBP *z* score from baseline at 1 y	−0.38 (0.98	−0.35 (0.92)	−0.03 (−0.28 to 0.22)	.81
Change in DBP *z* score from baseline at 1 y	−0.11 (1.32	−0.20 (1.27)	0.09 (−0.25 to 0.42)	.61
Change in total cholesterol from baseline at 1 y, mean (SD), mg/dL	−89.58 (89.19)	−69.88 (76.06)	−19.31 (−40.54 to 1.93)	.07
Change in albumin from baseline at 1 y, mean (SD), g/dL	0.93 (9.25 (0.82)	0.82 (8.16 (0.78)	0.11 (−0.09 to 0.31)	.20
Annualized eGFR slope estimate from baseline to month 12, ml/min/1.73 m^2^	−7.05 (2.69	−1.85 (2.80)	−5.54 (−13.62 to 2.54)	.18
Annualized serum creatinine slope estimates from baseline to month 12, mg/dL	0.04 (0.01)	0.01 (0.01)	0.02 (−0.00 to 0.05)	.09
Annualized serum urea nitrogen slope estimates from baseline to month 12, mg/dL	1.32 (0.31)	−0.70 (0.34)	2.07 (1.06 to 3.08)	<.001
Annualized serum cystatin C slope estimate from baseline to month 12, mg/L	0.01 (0.02	−0.02 (0.02)	0.03 (−0.02 to 0.08)	.28

Abbreviations: BMI, body mass index; DBP, diastolic blood pressure; eGFR, estimated glomerular filtration rate; HAZ, height for age *z* score; MMF, mycophenolate mofetil; NA, not applicable; SBP, diastolic blood pressure; TAC, tacrolimus; WHZ, weight for age *z* score.

SI conversion factors: To convert total cholesterol from mg/dL to mmol/L, multiply by 0.0259; albumin, from g/dL to g/L, multiply by 10; serum creatinine, from mg/dL to μmol/L, multiply by 88.4; urea nitrogen, from mg/dL to mmol/L, multiply by 0.357.

^a^ The between-group difference is given as hazard ratio or mean difference and corresponding 95% confidence intervals, with the TAC group being the reference.

^b^ Calculated as weight in kilograms divided by height in meters squared.

Moreover, during the study period, the TAC group had lower mean daily steroid use per unit body weight than the MMF group (0.22 mg/kg/day vs 0.34 mg/kg/day) (Table 3).

### Safety

AEs were reported in 94.81% of the TAC group and 92.59% of the MMF group (*P* = .62), with 34.07% and 36.30% of AEs attributed to the study medication, respectively (*P* = .80; eTable 4 in Supplement 3). Serious adverse events (SAEs) occurred in 8.89% of TAC group participants and 10.37% of MMF group participants (*P* = .84), with 3.70% and 5.19% of SAEs linked to the study medication, respectively (*P* = .77). Infections were the most common SAE, affecting 8 of 12 TAC patients with SAEs (66.7%) and 10 of 14 MMF patients with SAEs (71.4%). Medication-related SAEs included pneumonia (TAC: 1.48%; MMF: 0.74%), upper respiratory tract infections (MMF: 1.48%), and hyperglycemia (TAC: 0.74%). One case of superficial lower extremity erosion was reported in the MMF group (0.74%). Study withdrawal due to AEs occurred in 0.74% of participants in both groups (*P* > .99), while SAEs led to withdrawal in 0.74% of the TAC group and in no participants in the MMF group (*P* > .99). Acute kidney disease, defined as an eGFR less than 60 ml/min/1.73 m^2^, a decrease in eGFR of 35% or greater, or an increase in serum creatine of more than 50% within 3 months,^13^ was observed in 1 patient in the TAC group and 1 patient in the MMF group. In the TAC group, patient 07010 exhibited generally stable kidney function, with a significant deterioration noted at visit 7. In the MMF group, patient 09030 experienced a relapse of nephrotic syndrome during visit 5, leading to acute kidney disease. Following adjustment of steroid therapy, kidney function improved; however, the patient subsequently withdrew from the clinical trial. All SAEs were resolved or alleviated after symptomatic treatment (eTable 4 in Supplement 3). At the beginning of the treatment, the mean (SD) eGFR levels (calculated by the Schwartz formula^12^) in the TAC and MMF groups were 164.81 [35.71] mL/min/1.73 m^2^ and 165.54 [39.46] mL/min/1.73 m^2^, respectively. At the end of the treatment in both the TAC and MMF groups, the mean (SD) levels of serum creatinine (0.44 [0.14] mg/dL vs 0.43 [0.11] mg/dL [to convert serum creatinine from mg/dL to μmol/L, multiply by 88.4]), eGFR (158.4 [34.2] mL/min/1.73 m^2^ vs 163.4 [33.2] mL/min/1.73 m^2^), blood urea nitrogen (13.03 [3.73] mg/dL vs 11.04 [3.08] mg/dL [to convert urea nitrogen from mg/dL to mmol/L, multiply by 0.357]), and cystatin C (0.88 [0.18] mg/L vs 0.82 [0.18] mg/L) remained within normal ranges (Table 3). However, the more negative eGFR slope was seen in the TAC group (−7.0 vs −1.9; Table 3; eTable 5 in Supplement 3). The updated formula-calculated eGFR and the comparative results between the 2 groups are presented in eTable 5 in Supplement 3.

## Discussion

Both the Kidney Disease: Improving Global Outcomes (KDIGO) and IPNA guidelines recommend MMF and TAC as first-line therapies for FRNS and/or SDNS,^3,6^ but studies comparing MMF and TAC in FRNS and/or SDNS are lacking. In other autoimmune diseases, such as lupus nephritis, meta-analyses have shown that TAC achieves a higher overall kidney remission rate than MMF when overall kidney remission is used as an indicator of treatment efficacy.^14^ Furthermore, in patients with autoimmune hepatitis who did not respond to standard treatment, TAC led to a higher rate of complete response than MMF.^15^ In patients with pediatric nephrotic syndrome, only 2 previous uncontrolled, small-sample studies have suggested that these 2 agents might be equally effective.^7,8^ Considering the widespread use of these 2 drugs in pediatric FRNS and/or SDNS, it is imperative to compare their efficacy and safety.

In the STAMP randomized clinical trial, the efficacy and safety of TAC and MMF in treating pediatric patients with steroid-sensitive nephrotic syndrome (either FRNS or SDNS) were assessed. This study ranks among the most significant explorations into the application of steroid-sparing agents. We studied a homogeneous group of patients who had not been prescribed steroid-sparing agents within 2 years before enrollment, thereby minimizing the influence of other steroid-sparing agents on the evaluation of these 2 medications. Our data indicated that TAC was superior to MMF in this patient cohort, as demonstrated by a 65.0% reduction in relapse risk, increased 1-year relapse-free survival, and a reduced requirement for steroid therapy in the TAC-treated group. Additionally, the dropout rates differed between the TAC and MMF groups, with TAC showing a lower rate. In the MMF group, 6 patients dropped out due to failure to achieve the required MPA-AUC despite dose adjustments. The lower dropout rate in the TAC group suggests better tolerability and adherence, further supporting its superiority in maintaining remission among pediatric patients with FRNS or SDNS. To the best of our knowledge, this study is the first large-scale, multicenter, prospective, randomized clinical trial to directly compare the therapeutic benefits and safety profiles of TAC and MMF in children with FRNS or SDNS. At the end of the 1-year treatment period, the TAC group manifested a statistically significant increase in the relapse-free survival rate (82.22%) relative to the MMF cohort (58.52%). Furthermore, the incidence of relapses in the TAC group was substantially lower, with 17.78% of patients reporting 43 relapses, whereas 41.48% of patients in the MMF group reported 105 relapses. Additionally, the TAC cohort also benefited from a reduced cumulative dosage of steroids. Importantly, the time to first relapse was significantly shorter in the MMF group compared to the TAC group, but the relapse-free survival rates (58.52%) show that MMF still effectively prevents early relapse. This suggests TAC is better for sustaining long-term remission, while MMF may be sufficient for patients with less-aggressive relapse patterns.

In accordance with the 2021 KDIGO guidelines for managing glomerular diseases^6^ and the 2023 IPNA recommendations for diagnosing and managing children with steroid-sensitive nephrotic syndrome,^3^ steroid-sparing agents are strongly advised. However, definitive evidence for the optimal initial treatment remains elusive. In preceding uncontrolled studies,^7,8^ the efficacy of TAC was comparable or superior to that of MMF. The present rigorously designed STAMP trial further demonstrates the superiority of TAC over MMF in the maintenance of remission among patients with FRNS or SDNS. Notably, our trial revealed a relapse-free survival rate of 94.07% at 6 months and 82.22% at 12 months, which not only corroborates findings from several studies, but also exceeds outcomes reported in other prospective investigations, with 12-month remission rates achieved with TAC ranging between 43.2% and 63.3%.^16,17,18^

The safety profile of TAC and MMF revealed that most AEs were mild. The incidence of AEs that occurred after the administration of TAC or MMF was similar in the pooled TAC group and the MMF group (94.8% vs 92.6%). Notably, 1 patient in the TAC group withdrew because of worsening acne, and 4 patients in the MMF group withdrew because of repeated SAEs accompanied by nephrotic syndrome relapse, all of which are associated with infection. Consistent with other studies,^7,19^ infections represented the most frequent significant AE, affecting 8 of 12 participants in the TAC group (66.7%) and 10 of 14 participants in the MMF group (71.4%).

Drug-induced nephrotoxicity remains a significant concern for the long-term use of calcineurin inhibitors^20,21^; however, in the current trial, only 1 patient in the TAC group and 1 patient in the MMF group experienced acute kidney disease. These 2 patients recovered without receiving any special treatment, and their condition remained good after 1 year of study. In this trial, elevated baseline eGFR in both groups decreased after therapy, reflecting possible hyperfiltration in untreated nephrotic syndrome improved with therapy. However, the more pronounced decline in eGFR slope observed in the TAC group (−7.0 vs −1.9) suggests potential subclinical nephrotoxicity. This aligns with the known mechanisms of TAC, which can induce progressive kidney dysfunction through kidney vasoconstriction and tubular interstitial damage,^22,23^ even in the absence of overt acute kidney injury. Importantly, studies have indicated that the nephrotoxic effects of calcineurin inhibitors typically become more apparent with prolonged use (2-5 years).^24,25,26,27,28^ Given that this trial was limited to a 1-year follow-up, the long-term kidney risks associated with TAC remain uncertain and warrant further investigation. To mitigate these risks, close monitoring of kidney function and individualized dosing strategies are strongly recommended. In practice, MMF may be preferable for patients with baseline kidney vulnerability or those prioritizing long-term safety over maximal relapse control.

### Limitations

This study has several limitations. First, the duration of exposure to the investigational drugs, which was limited to 12 months, precluded a comprehensive evaluation of their long-term safety profile in patients with pediatric nephrotic syndrome. Second, our analysis was limited to relapse-free survival during active treatment, and postdiscontinuation outcomes were not examined. Third, the study’s geographic limitation to China necessitates validation of our findings across racially and ethnically diverse populations with FRNS or SDNS. Fourth, there is a lack of kidney pathology information for patients, especially kidney pathology reports after 1 year of TAC use. Finally, this study did not systematically explore the impact of the 2 treatment protocols on quality of life, an area that warrants further investigation.

## Conclusions

Collectively, in the STAMP randomized clinical trial, a 1-year regimen of TAC was associated with a significantly greater relapse-free survival period and a reduced requirement for corticosteroids compared with MMF in pediatric patients who presented with steroid-sensitive nephrotic syndrome characterized by frequent relapses or steroid dependency. Treatment effectiveness was sustainable during the course of treatment.

## Notes

Supplement 1. Trial Protocol

Supplement 2. Statistical Analysis Plan

Supplement 3. eTable 1. Reasons for Active Withdrawal eTable 2. Baseline Characteristics of the Retained Participants eTable 3. 1-Year Relapse-Free Survival Using Cox Regression (FAS and PPS) eTable 4. Adverse Events eTable 5. Comparison of eGFRs Estimated via Various Formulas and Assessment of Differences in Renal Function Between the TAC and MMF Groups via the MMRM eFigure. Subgroup Analysis of Relapses Within 1-year eMethods 1. Pharmacokinetic Studies eMethods 2. Statistical Analysis eReferences.

Supplement 4. Data Sharing Statement
