# Supplementary material for: Tacrolimus or Mycophenolate Mofetil for Frequently Relapsing or Steroid-Dependent Nephrotic Syndrome: A Randomized Clinical Trial
Source: JAMA Pediatr. 2025 May 12;179(7):722–9. doi: 10.1001/jamapediatrics.2025.0765 (PMC12070277; doi:10.1001/jamapediatrics.2025.0765)
Supplement: Supplement 1. — Trial Protocol [file jamapediatr-e250765-s001.pdf]

Study of Tacrolimus vs Mycophenolate Mofetil in Pediatric patients with Frequently Relapsing or Steroid-dependent Nephrotic Syndrome: a Prospective, Randomized, Multicenter, Open-label, and Parallel-arm Study on Efficacy and Safety (the STAMP study)

**Main research unit:**	The Children's Hospital, Zhejiang University School of Medicine
**Principal investigator:**	Mao Jianhua
**Sponsor:**	Hangzhou Zhongmeihuadong Pharmaceutical Co., Ltd.
**Protocol No.:**	NSTAMF02
**Version No.:**	V 2.1
**Version date:**	June 10, 2019

This clinical trial protocol is confidential and owned by the Sponsor. Without the permission from the Sponsor, it is not allowed to provide the contents, in part or in full, to any third party (group or individual).

Contents

Contents 2

Compliance Statement 5

For investigator's signature 6

For Sponsor's signature 7

Abbreviations 8

Protocol Abstract 10

1.1 Abstract 10

1.2 Research Plan 12

1.3 Clinical trial flow chart 13

1. Background 17

2. Purpose 20

2.1 To observe the efficacy of test drugs on the frequently relapsing or steroid-dependent nephrotic syndrome in pediatric patients 20

2.2 To observe the safety outcomes of test drugs 20

3. Design basis, type, principle and case allocation 20

3.1 Design basis 20

3.2 Design type 20

3.3 Design principle and case allocation 20

4. Selection of cases 21

4.1 Inclusion criteria 21

4.2 Exclusion criteria 21

4.3 Elimination criteria 22

4.4 Withdrawal criteria 22

4.5 Drop-out and treatment 23

4.6 Relevant definitions in inclusion and exclusion criteria 23

5. Test drugs and their allocation 24

5.1 Drug name and specifications 24

5.2 Administration method, dose, route and course of treatment 24

5.3 concomitant medication 26
5.4 Management of test drugs 26
5.5 Medication adherence 28
6. Test process and steps 28
7. Evaluation of efficacy and safety 28
7.1 Primary efficacy outcomes 28
7.2 Efficacy evaluation 28
7.3 Safety evaluation 29
8. Reporting adverse events 29
8.1 Definition of adverse events 29
8.2 Definition of serious adverse event 33
8.3 Records of adverse events 34
8.4 Report of serious adverse events 36
9. Quality control and assurance of the trial 36
9.1 Quality control measures in laboratory 36
9.2 Quality control and assurance in the trial process 37
9.3 Measures to improve subject compliance 39
9.4 Quality control and quality assurance systems 39
10. Data management 39
10.1 Data management system 39
10.2 Data record 40
10.3 Data supervision 40
11. Statistical analysis 40
11.1 Analysis data set 40
11.2 Statistical method 40
11.3 Content of statistical analysis 41
11.3.1 Basic value equilibrium analysis 41
11.3.2 Effectiveness analysis 41
11.3.3 Primary efficacy analysis 41
11.3.4 Secondary efficacy analysis 42

11.3.5 Other efficacy analysis 42
11.3.6 Safety analysis 42
12. Ethical principles 42
12.1 Ethical review 42
12.2 Benefits and risks 43
12.3 Informed consent process 43
12.4 Medical treatment and protection of subjects 43
12.5 Protection of subject privacy 43
13. Ethical requirements 43
14. Data preservation 44
15. Expected progress 44
16. Follow-up and medical measures after the trial 45
17. References 45
Appendix 1 Estimation of glomerular filtration rate 47
Appendix 2 Blood pressure reference values for children 48
Appendix 3 Comparison and acceptance criteria of laboratory observation indexes 50
Appendix 4 Parameters and results sample size calculation 51

Compliance Statement

This clinical research will comply with the *Good Clinical Practice* (GCP) and relevant regulations of the China Food and Drug Administration (CFDA).

The investigators and clinical research staff responsible for the implementation, management and supervision of this project need to complete GCP training.

This Protocol, Informed Consent Form, recruitment materials and other subject materials have been

Submitted to the Ethics Committee for review and approval. Subject recruitment would begin after the Protocol and Informed Consent Form are approved by the Ethics Committee. Any revision of the Protocol during this research must be reviewed and approved by the Ethics Committee before its implementation. In addition, all revisions to the Informed Consent Form shall be approved by the Ethics Committee. Subjects who have signed the old Informed Consent Form and are still in the groups shall re-sign the new version of Informed Consent Form.

For investigator's signature

I hereby agree:

- To be responsible for the normal operation of this research in our center.
- To carry out this research in accordance with the research protocol and its amendment as well as the relevant research implementation specifications provided by Hangzhou Zhongmeihuadong Pharmaceutical Co., Ltd.
- Not to change or violate this research protocol without the review and written approval from the Sponsor and the Medical Ethics Committee, unless it is for the purpose to eliminate the harm to the subjects as soon as possible or for needs of trial management (requiring permission from the corresponding regulatory authority).
- I am fully familiar with the rational application of the research medicine described in the Protocol and the relevant materials provided by the Sponsor, including but not limited to the Clinical Investigator Manual or the corresponding documents provided by Hangzhou Zhongmeihuadong Pharmaceutical Co., Ltd., as well as the supplementary contents of the Investigator Manual (if necessary) and the product description.
- I understand and will abide by the GCP and all relevant regulations and rules.
- To ensure that all personnel assisting me in this research are fully familiar with the research medicine from Hangzhou Zhongmeihuadong Pharmaceutical Co., Ltd. as well as the responsibilities and functions related to this research as described in this Protocol.
- I have been informed that some regulatory authorities require the Sponsor to provide the ownership interests of the investigators in the research medicine and the financial relationship with the Sponsor. Hangzhou Zhongmeihuadong Pharmaceutical Co., Ltd. will use or provide such information for the purpose of complying with the requirements of the regulatory authorities.

Research center:

Investigator (signature):

Date: July 25, 2019

For Sponsor's signature

(Sponsor) Statement: Our company will be responsible for initiating, applying for, organizing, supervising and inspecting this clinical trial and providing trial funds in accordance with the provisions of GCP. In particular, we will provide treatment compensation to the subjects who would suffer from the trial-related damage or death during the trial, and provide legal and economic guarantees to the investigators.

Sponsor representative name (signature)____________

Sponsor name (seal)_____________

Date:__________(YYYY/MM/DD)

Abbreviations

Abbreviations	Description
ALT	Alanine aminotransferase
ALB	Albumin
AUC	Area under curve
ACEi	Angiotensin converting enzyme inhibitor
ARB	Angiotensin II receptor blocker
AE	Adverse event
ANCOVA	Analysis of covariance
BID	Bis in die
CMV	Cytomegalovirus
CsA	Cyclosporin A
CR	Complete remission
CSR	Clinical Study Report
CRF	Case Report Form
CMH	Cochran's and Mantel-Haenszel
DAE	Drug adverse event
EB	Epstein-Barr
EBV-DNA	Epstein-Barr virus deoxyribonucleic acid
eGFR	Glomerular filtration rate
eCRF	Electronic Case Report Form
FAS	Full analysis set
FR	Frequent relapse
FDA	Food and Drug Administration
FK506	Tacrolimus
FK-BP-12	Tacrolimus binding protein-12
FK-BP-52	Tacrolimus binding protein-52
FK506-FKBP	Tacrolimus - tacrolimus binding protein
FRNS	Frequently relapsing nephrotic syndrome
GCP	Good Clinical Practice
g	Gram
GTP	Guanine nucleotide
HBV	Hepatitis B virus
HbsAg	Hepatitis B surface antigen
HbeAg	Hepatitis B E antigen

HbcAb	Hepatitis B core antigen
HCV	Hepatitis C virus
hr	Hour
ITT	Intentionality
LSMEAN	Least squares mean
LC-MS	Liquid chromatography-mass spectrometry
MMF	Mycophenolate mofetil
MPA	Mycophenolic acid
MPA-d3	Mycophenolic acid-d3
MPA-AUC	Mycophenolic acid - Area under curve
MI	Myocardial infarction
mg	Milligram
NR	No remission
PP	Per-protocol
PR	Partial remission
PML	Progressive multifocal leukoencephalopathy
PRCA	Pure red cell aplasia
PPS	Per-protocol set
SAS 9.4	STATISTICAL ANALYSIS SYSTEM Version 9.4
SD	Steroid-dependent
SDNS	Steroid-dependent nephrotic syndrome
SRNS	Steroid-resistant nephrotic syndrome
SCV	Site close-out visit
SAE	Serious adverse event
SOP	Standard Operation Procedures
SS	Safety set
Scr	Serum creatinine
TBiL	Total bilirubin
TIMP-1	Tissue inhibitor of metalloproteinase-1
TIA	Transient ischemic attack
WHO	World Health Organization
WBDC	Web-based data collection
α	Inspection level
β	Power of test
δ	Cut-off value

Protocol Abstract

1.1 Abstract

Title: Study of Tacrolimus vs Mycophenolate Mofetil in Pediatric Patients with Frequently Relapsing or Steroid-dependent Nephrotic Syndrome: a Prospective, Randomized, Multicenter, Open-label, and Parallel-arm Study on Efficacy and Safety

Objective: To evaluate the efficacy and safety of tacrolimus and mycophenolate mofetil in the treatment of frequently relapsing and steroid-dependent nephrotic syndrome in children, in order to provide a more effective and safer choice for the treatment of nephrotic syndrome in pediatric patients.

Design: Prospective, randomized, multicenter, open-label, and parallel-arm trial

Primary center: The Children's Hospital, Zhejiang University School of Medicine

Test drug name: Saikeping® Mycophenolate Mofetil Dispersible Tablets; Saifukai® Tacrolimus Capsules

Subjects: Pediatric patients with confirmed steroid-sensitive but frequently relapsing or steroid-dependent primary nephrotic syndrome

Treatment course: 1 year

Medication:

(1) Tacrolimus group: FK506 dose: 0.05-0.10 mg/kg/day, BID; in the induction period (the first six months after administration), the trough plasma concentration of tacrolimus is maintained at 5-10 ng/mL; in the maintenance period (the last six months after administration), the trough concentration is less than 5 ng/mL.

(2) Mycophenolate mofetil group: MMF dose: 20-30 mg/kg/day, BID; in the induction period (the first six months after administration), the plasma concentration MPA-AUC is maintained at 30-50 μg.h/mL; in the maintenance period (the last six months after administration), the MPA-AUC is ≤40 μg.h/mL.

(3) Prednisone dose: The initial dose is 1.0-1.5 mg/kg every other day or 0.5-0.75 mg/kg every day. The dose shall be reduced after 4 weeks by 0.25 mg/kg (every other day) or 0.125 mg/kg (every day) every 2-4 weeks. If the condition is stable, continue to reduce and maintain at 5mg (body surface area > 1 m^2) and 2.5mg (body surface area < 1 m^2) every other day.

Efficacy outcomes:

Primary observation outcomes: 1-year relapse-free survival

Secondary observation outcomes: Renal function; cumulative steroid dosage; blood pressure; height; body weight; blood lipids; hemoglobin; serum albumin; urinary protein/creatinine (morning urine); 24hr urinary protein quantification (applicable to subjects > 3 years old); frequency of

relapses; the time of the first relapse after enrollment; relapse-free rate in the first six months after enrollment; adverse reactions;

Safety outcomes: Changes of vital signs (respiration and heart rate), blood and urine routine, liver function, blood glucose, ECG, chest X-ray, and observation of clinically reported adverse events and adverse reactions.

Sample size: 270 cases. Including 135 cases in MMF Group, and 135 cases in FK506 Group.

Statistical analysis: SAS 9.4 software for data statistical analysis.

1.2 Research Plan

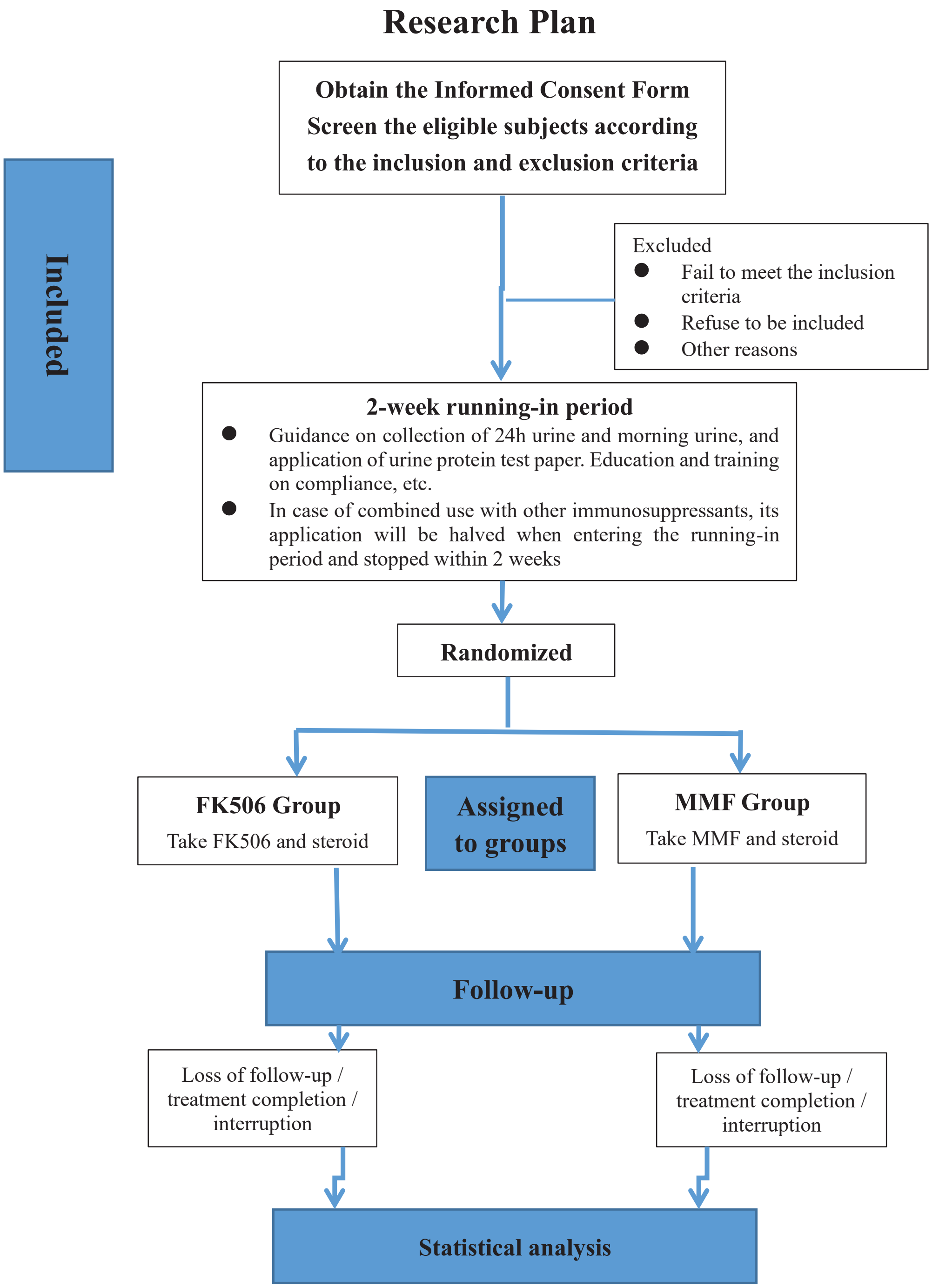

1.3 Clinical trial flow chart

Clinical trial flow chart

Items	Screening and enrollment period		Treatment and follow-up period							
	Screen (V0)	Visit 1 (V1)	Visit 2 (V2)	Visit 3 (V3)	Visit 4 (V4)	Visit 5 (V5)	Visit 6 (V6)	Visit 7 (V7)	Visit 8 (V8)	Visit 9 (V9)
Follow-up week	Day -30 - 0	Included	Day 7	Day 28	Day 56	Day 112	Day 168	Day 224	Day 280	Day 364
Sign the Informed Consent Form	√									
Inclusion/Exclusion Criteria		√								
Stop the original immunosuppressants after 2 weeks	$	$								
Randomized		√								
General data	√									
Medical history (course of disease, frequency of relapses in 1 year and 6 months before enrollment, steroid dosage)	√									
Vital signs	√	√	√	√	√	√	√	√	√	√

Items	Screening and enrollment period		Treatment and follow-up period							
	Screen (V0)	Visit 1 (V1)	Visit 2 (V2)	Visit 3 (V3)	Visit 4 (V4)	Visit 5 (V5)	Visit 6 (V6)	Visit 7 (V7)	Visit 8 (V8)	Visit 9 (V9)
Height and weight	√	√	√	√	√	√	√	√	√	√
Physical examination	√	◎	√	√	√	√	√	√	√	√
Blood routine examination	√	◎	√	√	√	√	√	√	√	√
Urine routine examination	√	◎	√	√	√	√	√	√	√	√
Fasting blood glucose	√		√	√	√	√	√	√	√	√
Serum liver function	√	◎	√	√	√	√	√	√	√	√
Blood lipid	√	◎	√	√	√	√	√	√	√	√
Serum renal function	√	◎	√	√	√	√	√	√	√	√
Blood EBV-DNA, and CMV antibody	√									√
24h urinary protein (>3 years old)	√	◎	√	√	√	√	√	√	√	√
Urinary protein / creatinine	√	◎	√	√	√	√	√	√	√	√
eGFR	√	◎	√	√	√	√	√	√	√	√

Items	Screening and enrollment period		Treatment and follow-up period							
	Screen (V0)	Visit 1 (V1)	Visit 2 (V2)	Visit 3 (V3)	Visit 4 (V4)	Visit 5 (V5)	Visit 6 (V6)	Visit 7 (V7)	Visit 8 (V8)	Visit 9 (V9)
Tacrolimus concentration in plasma			√	√	√	√	√	√		√
Mycophenolic acid concentration in plasma			√				√			
Chest X-ray	√									√
ECG	√									√
Markers of hepatitis B and hepatitis C virus	√									
Examination of Mycobacterium tuberculosis and fungi	√									
Biological samples of blood and urine	√						√			√
Drug distribution and measurement		√	√	√	√	√	√	√	√	√
Guidance on the administration of test drug		√	√	√	√	√	√	√	√	√

**Items**	**Screening and enrollment period**		**Treatment and follow-up period**							
	Screen (V0)	Visit 1 (V1)	Visit 2 (V2)	Visit 3 (V3)	Visit 4 (V4)	Visit 5 (V5)	Visit 6 (V6)	Visit 7 (V7)	Visit 8 (V8)	Visit 9 (V9)
Clinical efficacy (relapse or no)			√	√	√	√	√	√	√	√
Concomitant medication	√	√	√	√	√	√	√	√	√	√
Adverse event			√	√	√	√	√	√	√	√
Record case report form	√	√	√	√	√	√	√	√	√	√

Remarks:

1. Liver function: ALT, TBiL, ALB; Renal function: creatinine, urea nitrogen, uric acid, Cystatin C;
2. Blood lipids: Triglycerides, cholesterol; Urinary protein / creatinine: morning urine; Markers of hepatitis B and hepatitis C virus: HBsAg, HBeAg, HBcAb, HCV;
3. Mycobacterium tuberculosis examination: T-sport; Fungal examination: fungal G test or other; Estimated glomerular filtration rate: eGFR = K × height (cm) / serum creatinine (mg / dL) (see Appendix);
4. Visit time window: ±3 days for visit 2, and ±7 days for other visits;
5. $ items are applicable to the pediatric patients with combined use of other immunosuppressants before enrollment; ◎ items are optional, depending on the judgment and decision of the investigators;
6. This research accepts the examination data from our hospital within 2 weeks before the screening period, and no repeated test is needed.

1. Background

Primary nephrotic syndrome, the most common glomerular disease in children, accounts for about 90% of the total nephrotic syndrome cases in pediatric patients[1]. It is reported that the annual incidence rate of pediatric nephrotic syndrome is about 2-4/100000, and the prevalence rate is about 16/100000[2, 3]. The statistics of hospitalized children in some provincial and municipal hospitals in China show that primary nephrotic syndrome accounts for about 20% of hospitalized children with urinary system diseases[4]. Renal biopsy shows minimal lesions in more than 80% of patients. This disease can occur at any age, but it mostly occurs at the age of 2-6. It is more common in boys, with a male/female ratio of about 3:2. Since the 1950s, glucocorticoids have been recognized as the first-line treatment. About 80%-90% of pediatric patients with primary nephrotic syndrome can achieve complete remission after initial steroid treatment, but 76%-93% of the pediatric patients have relapses, of which 45%-50% show frequent relapses (FR) or steroid dependence (SD)[5]. The relapses of proteinuria, especially frequently relapsing/steroid-dependent nephrotic syndrome, have always been a very difficult and urgent problem for clinicians. Some pediatric patients may have steroid resistance after repeated relapses and eventually develop into chronic dysfunction. The long-term or repeated use of steroids at high doses will lead to side effects such as obesity, growth inhibition, hypertension, diabetes, glaucoma, cataract, osteoporosis and serious infection. Its combined use with traditional immunosuppressants such as cyclophosphamide and cyclosporine A (CSA) will bring some serious irreversible side effects. Therefore, it is particularly important to explore new immunosuppressants and their application schemes.

Tacrolimus is a 23-membered macrolide antibiotic extracted from the fermentation of Streptomyces. It was used clinically in 1989 for the first time, and officially used in the United States after being approved by the U.S. Food and Drug Administration (FDA) in 1995. It was registered as an imported drug in China in 1998 and gradually used in clinical application. Tacrolimus, a new type of calcineurin inhibitor, can be combined with FK506 binding protein-12 (FK-BP-12) and FK506 binding protein-52 (FK-BP-52) receptors in the cytoplasm of T lymphocytes in vivo to form FK506-FKBP complex, with an function of inhibiting the phosphorylase activity of calmodulin phosphatase, thus inhibiting the proliferation of T lymphocytes. This indirectly affects the production of B lymphocytes and antibodies, and finally causes immunosuppression [6]to achieve the purpose of treating glomerular diseases. Its immunosuppressive effect is 10 - 100 times that of cyclosporine A[7]. In addition to its strong immunosuppressive effect, tacrolimus also has its non-immunosuppressive effects: (1) It can produce hormone-like effect or reduce the dosage of hormone; (2) It can significantly down-regulate the expression of tissue inhibitor of metalloproteinase-1 (TIMP-1), while CsA can up-regulate the expression of TIMP-1. Therefore, tacrolimus shows weaker effect on tissue fibrosis than CsA; (3) It can inhibit platelet aggregation, reduce thrombosis

and reduce inflammatory reaction[8]; (4) It has podocyte protection effect. The studies by Li Xiayu et al.[[9] have shown that as compared with cyclophosphamide, tacrolimus can induce the remission of nephrotic syndrome more quickly in adult patients with steroid dependence and minimal lesion in pathology. About 72.7% of patients using tacrolimus can successfully stop the application of steroid. In the treatment of steroid-dependent or steroid-resistant focal segmental sclerosis, the studies by Hong Ren et al. [10]have shown that tacrolimus and cyclophosphamide have similar effects, both of which can reduce urinary protein, improve renal function and blood albumin level. The previous clinical studies of the Department of Nephrology of the Children's Hospital, Zhejiang University have also found that tacrolimus can effectively reduce the frequent relapses or the relapse frequency of steroid-dependent nephrotic syndrome, and down-regulate the total amount of hormone use.

Mycophenolate mofetil (MMF) is a precursor of mycophenolic acid (MPA). Mycophenolic acid is a reversible and non-competitive inhibitor of hypoxanthine monophosphate dehydrogenase, a rate limiting enzyme in purine synthesis. It can significantly inhibit the classical synthesis pathway of guanine nucleotide (GTP), without any impact on other synthesis pathways and remedial synthesis pathways.[11] MMF will not significantly damage liver, kidney or bone marrow cells, and can avoid cytotoxic damages caused by other immunosuppressants[12]. MMF can quickly inhibit the synthesis of antibodies, and effectively inhibit the secretion of surface adhesion molecules, to play the pharmacological role in anti-inflammatory response. In addition, MMF can also significantly inhibit the proliferation rate of vascular smooth muscle cells and endothelial cells, inhibit the production of cell adhesion molecules and aggregation of a large number of leukocytes near the inflammatory tissues, enhance its pharmacological effect of immunosuppression by inducing lymphocyte apoptosis, and finally significantly reduce the degree of renal parenchyma damage and avoid the occurrence of glomerulosclerosis[13]. Animal researches have also confirmed that MMF can play a great role in the process of activation under the activation of inflammatory cells, transdifferentiation of myofibroblasts and massive aggregation of extracellular matrix, so as to prevent the pathological process of renal interstitial fibrosis[14]. In 1998, Briggs et al. first used MMF in the treatment of adult steroid-resistant or steroid-sensitive but recurrent nephrotic syndrome, showing a good curative effect[15]. Then, many studies at home and abroad have shown that mycophenolate mofetil can reduce the relapse frequency of frequently relapsing nephrotic syndrome and reduce the dosage of steroid. Recent studies by Gellermann et al. [16]have shown that cyclosporine A is better than mycophenolate mofetil in reducing the relapse frequency of frequently relapsing nephrotic syndrome, but mycophenolate mofetil is significantly better than cyclosporine A in protecting the renal function. In short, it is believed that MMF can effectively reduce the relapse rate of nephropathy, reduce and even stop the steroid use, with less adverse reactions and no nephrotoxicity. It is an effective and safe immunosuppressant for the treatment of FRNS in pediatric patients.

In addition, domestic and foreign expert guidelines, such as *KDIGO Clinical Practice Guideline for Glucorulonephritis (2012)*, *Evidence-based Guideline for the Diagnosis and Treatment of Steroid-resistant Nephrotic Syndrome (2016)* and *Evidence-based Guideline for the Diagnosis and Treatment of Steroid-sensitive and Recurrent/Dependent Nephrotic Syndrome in Pediatric Patients (2016)*, all recommend tacrolimus and mycophenolate mofetil for frequently relapsing/steroid-dependent nephrotic syndrome[17-19]. Among these two types of drugs, tacrolimus with the dual effects of immunosuppression and podocyte protection, is more widely used in Department of Nephrology, and mycophenolate mofetil has also gradually obtained the attention from nephrologists in recent years because of its non-nephrotoxicity, less adverse reactions and high safety. However, currently there are no multicenter studies comparing the efficacy of tacrolimus and mycophenolate mofetil in pediatric patients with frequently relapsing/steroid-dependent nephrotic syndrome at home and abroad. Therefore, the purpose of this research is to observe the efficacy and safety of tacrolimus and mycophenolate mofetil in the treatment of frequently relapsing/steroid-dependent nephrotic syndrome in pediatric patients with a prospective, randomized, multicenter, open-label and parallel-arm approach, and evaluate their safety, hoping to provide a more effective and safe treatment scheme for frequently relapsing/steroid-dependent nephrotic syndrome in pediatric patients.

2. Purpose

2.1 To observe the efficacy of test drugs on the frequently relapsing or steroid-dependent nephrotic syndrome in pediatric patients

Efficacy outcomes:

Primary observation outcomes: 1-year relapse-free survival

Secondary observation outcomes: Renal function; cumulative steroid dosage; blood pressure; height; body weight; blood lipids; hemoglobin; serum albumin; urinary protein/creatinine (morning urine); 24hr urinary protein quantification (applicable to subjects > 3 years old); frequency of relapses; the time of the first relapse after enrollment; relapse-free **survival** in the first six months after enrollment; adverse reactions;

2.2 To observe the safety outcomes of test drugs

Changes of vital signs (respiration and heart rate), routine blood and urine tests, liver function, renal function, blood glucose, ECG, chest X-ray, and observation of clinically reported adverse events and adverse reactions.

3. Design basis, type, principle and case allocation

3.1 Design basis

- *Pharmaceutical Administration Law of the People's Republic of China*, 2001
- *Drug Registration Regulation*, 2007
- *Good Clinical Practice (GCP)*, 2003
- *Declaration of Helsinki*, 2008
- Results of pharmacodynamic and toxicological studies of tacrolimus and mycophenolate mofetil ingredients, and current clinical trial experience

3.2 Design type

This trial adopts a prospective, randomized, multicenter, open-label and parallel-arm clinical research method. The whole observation period is 1 year for this trial. See P12 "Clinical trial flow chart" for details.

3.3 Design principle and case allocation

3.3.1 Difference analysis

In this research, Logrank method is used to compare the difference of 1-year relapse-free survival between the two groups.

Eligible patients will be randomly divided into FK506 Group or MMF Group at the ratio of 1:1. The random codes are generated by the computer and the random envelopes are made according to these random codes. Grouping information is attached in each envelope. Each code is provided with a corresponding random envelope, and the random numbers are given to the subjects from small to large according to the time sequence when the subjects meet the inclusion criteria. The corresponding random envelopes can only be opened when grouping. Any random number falling off midway after use cannot be used again.

3.3.2 Determination of the number of cases

It is estimated that 1-year relapse-free survival is 0.56 in MMF Group, and 0.70 in FK506 Group. The enrollment time of this research is expected to be 2 years, and the total time of this research is 3 years, with a test level $\alpha = 0.05$, and a power of test $1-\beta = 80\%$. It is estimated to have 114 patients in MMF group and 115 patients in FK506 group, with a total of 229 patients (see Appendix 4 for details). With the consideration of 15% loss of follow-up, a total of 270 patients are needed, including 135 patients in each group.

4. Selection of cases

4.1 Inclusion criteria

Only those who meet all the conditions listed in this column can be considered for inclusion in this research:

(1) Steroid-sensitive nephrotic syndrome with frequent relapse (FRNS) or steroid dependence (SDNS);

(2) Age: 2-18 years old;

(3) Normal renal function: eGFR≥90mL/min/1.73m^2;

(4) At the time of enrollment, urinary protein <1+ or urinary protein/creatinine <0.2g/g (< 20 mg/mmol) in the morning for 3 consecutive days or more;

(5) No use of tacrolimus, mycophenolate mofetil, cyclosporine A, rituximab or cyclophosphamide within 2 years before enrollment;

4.2 Exclusion criteria

Anyone with one of the following conditions cannot be included in this research:

(1) Steroid-resistant nephrotic syndrome (SRNS);

(2) Family history of nephrotic syndrome, chronic glomerulonephritis or uremia;

(3) Leukopenia (leukocyte $\leq 3.0*10^9$/L);

(4) Moderate and severe anemia (hemoglobin < 9.0 g/dL);

(5) Thrombocytopenia (platelet count < $100*10^{12}$/L);

(6) Those with positive HBV serological indexes (HBsAg or/and HBeAg or/and HBcAb), positive HCV or abnormal liver function (ALT or total bilirubin exceeding 2 times or more of the upper limit, with a continuous increase for 2 weeks);

(7) There are chronic active infections such as Epstein-Barrvirus, cytomegalovirus or Mycobacterium tuberculosis, and the usage of steroids and immunosuppressive agents may aggravate the state of an illness;

(8) Secondary nephrotic syndrome (such as purpura nephritis ,lupus nephritis,etc);

(9) Those who with hematological or endocrine system diseases as well as serious organs illness such as heart, liver or kidney;

(10) Other autoimmune diseases or primary immunodeficiency or tumors;

(11) Those who was known be sensitized to tacrolimus, mycophenolate mofetil, glucocorticoids or any ingredient of the above drugs;

(12) Those who have participated in other clinical trials within three months before enrollment;

(13) Those who was not suitable for participating this study judged by investigator;

4.3 Elimination criteria

- Those who fail to take the test drugs as required, with a compliance <80% or >120%;
- Those who cannot cooperate well or complete the follow-up as planned, and those who change the drug dose at will;

4.4 Withdrawal criteria

Patients are free to withdraw from the trial at any time without any reason.

Patients must withdraw from this research in the following conditions:

- Withdrawal of the Informed Consent Form;
- Violation of the inclusion criteria or compliance with the exclusion criteria;
- No administration of the test drug according to the Protocol for 14 consecutive days due to various reasons;
- For two consecutive times, the measured value of SCr with an interval of at least 4 weeks has increased more than twice the baseline, and eGFR < 60mL/min.1.73m^2;
- The target concentration of tacrolimus or mycophenolate mofetil can not be reached after dose adjustment for one month;
- ALT or total bilirubin levels reach more than 2 times of the upper limit after medication, with a continuous increase for 2 weeks; if ALT or total bilirubin levels still rise to more than 2 times of the upper limit after 2 weeks of liver protection drug treatment, stop the test drug. In case of no recovery after 2 weeks of drug withdrawal, the patient shall withdraw from the test;

- Occurrence of unknown adverse events of unacceptable nature, severity and/or duration, or occurrence of known unacceptable adverse events or the occurrence frequency exceeding expectations;
- The clinical events or safety-affecting events result in withdrawal from the clinical trial, and the investigators believe that such subjects shall withdraw from the trial;
- If pregnancy occurs during treatment, withdraw from the trial;
- Unauthorized use of drugs prohibited by this research (see "5.3 Concomitant medication");

4.5 Drop-out and treatment

4.5.1 Definition of drop-out: All patients who have completed the Informed Consent Form and screened to be enrolled in the trial have the right to withdraw at any time. Whenever and for whatever reason, subjects who fail to complete the observation period specified in the Protocol are considered as drop-out cases, including the following situations:

- The subject has serious adverse events and the clinician believes that he/she should withdraw from the clinical trial;
- The subject shows aggravated condition or has other symptoms affecting the trial observation during the trial, and the clinician believes that he/she should withdraw from the clinical trial;
- Important deviations occur in the implementation of clinical trial protocol, such as poor compliance, which would cause difficulty in evaluating the drug effect;
- The subject is unwilling to continue the clinical trial and asks to withdraw from the clinical trial, or loss of follow-up has occurred although no request to withdraw from the trial is proposed.

4.5.2 Treatment of drop-off cases: The investigators shall contact the drop-off subjects by means of visiting, making an appointment for follow-up, calling, letter and so on, to ask the reasons, record the last medication time and complete the evaluation items that can be completed. For the cases withdrawing from the trial due to allergy or other adverse reactions and ineffective treatment, the investigators shall take corresponding treatment measures according to the actual situation of the subjects. The investigators shall properly keep the relevant test data of the drop-off cases, not only for filing, but also for full analysis and statistics.

4.6 Relevant definitions in inclusion and exclusion criteria

4.6.1 Steroid-sensitive nephrotic syndrome (SSNS): Complete remission after 4 weeks of prednisone or prednisolone at standard dose [2mg/kg.d or 60mg/m^2. d].

4.6.2 Steroid-resistant nephrotic syndrome (SRNS): Lack of complete remission at 4 weeks of therapy with daily prednisone or prednisolone at standard dose [2mg/kg.d or 60mg/m^2. d].

4.6.3 Steroid-dependent nephrotic syndrome (SDNS): Two consecutive relapses during therapy with prednisone or prednisolone (either at full dose or during tapering) or within 15 days of prednisone or prednisolone discontinuation.

4.6.4 Frequently relapsing nephrotic syndrome (FRNS): ≥2 relapses per 6 months within 6months of disease onset or 24 relapses per 12 months in any subsequent 12-month period.

4.6.5 Secondary nephrotic syndrome: It refers to the nephrotic syndrome with clear causes. Secondary nephrotic syndrome can be caused by autoimmune diseases (such as systemic lupus erythematosus), diabetes, infections (such as bacteria and hepatitis B virus), circulatory system diseases, drug poisoning, etc.

5. Test drugs and their allocation

5.1 Drug name and specifications

(1) Tacrolimus capsules (trade name: Saifukai®， capsules, 1.0 mg/capsule, 0.5 mg/capsule); Storage conditions: Keep away from sunlight, and store in a dry place below 25°C after sealing.

(2) Mycophenolate mofetil dispersible tablets (trade name: Saikeping®, tablets, 250 mg/tablet); Storage conditions: Store under 30°C and keep away from sunlight.

(3) Storage conditions of prednisone tablets (tablets, 5 mg/tablet): Keep away from sunlight, and store at room temperature after sealing.

[Prednisone tablets can be replaced by other glucocorticoids during the test, but the dose shall have the same therapeutic effect, for example prednisone 5mg = prednisolone 5mg, prednisone 5mg = methylprednisolone 4mg, etc.]

5.2 Administration method, dose, route and course of treatment

5.2.1 Initial administration protocol

FK506 dose: 0.05-0.10 mg/kg/day, BID

Maintained concentration: In the induction period (the first six months after treatment) (visit 2, 3, 4, 5 and 6), the trough plasma concentration of tacrolimus is 5 - 10 ng/mL;

In the maintenance period, drug dose is gradually reduced when the patient's condition is stable:

In the last six months after administration (visit 7, 8, and 9), the trough concentration **is <5 ng/mL**

MMF dose: 20-30 mg/kg/day, BID

Plasma concentration (AUC) [Formula: MPA-AUC =7.75+(6.49*C0)+(0.76*C0.5)+(2.43*C2)]

Maintained concentration: In the induction period (the first six months after treatment) (visit 2, 3, 4, 5 and 6)

MPA-AUC=30 - 50 µg.h/mL

In the maintenance period, drug dose is gradually reduced when the patient's condition is stable:

In the last six months after administration (visit 7, 8, and 9), MPA-AUC≤40 µg.h/mL

Prednisone dose: The initial dose is 1.0-1.5 mg/kg every other day or 0.5-0.75 mg/kg every day. The dose shall be reduced after 4 weeks by 0.25 mg/kg (qd alt) or 0.125 mg/kg (every day) every 2-4 weeks. If the condition is stable, continue to reduce to 5mg (body surface area > 1 m^2) and 2.5mg (body surface area < 1 m^2) qd alt. Maintain at such doses until this research is completed.

5.2.2 Treatment of relapses

If the disease is complicated with infection in the relapse for the pediatric patients, anti-infection treatment can be given first. After infection control, if the disease still shows relapse, the prednisone dose shall be restored to 1.0-1.5 mg/kg per day, with a maximum dose ≤60mg; If there is no infection at the time of relapse in the pediatric patients, the prednisone dose should be restored to 1.0-1.5 mg/kg per day directly, with a maximum dose ≤60mg. 5-7 days after the urine protein turns into negative results, the prednisone dose is reduced by 1.0-1.5 mg/kg every other day or 0.5-0.75mg/kg per day × 4 weeks. Then the dose is reduced as per the above steroid reduction method, while the dose of MMF or FK506 remains unchanged.

5.2.3 Dose adjustment of tacrolimus (FK506)

5.2.3.1 Adjust the dose according to the trough plasma concentration of tacrolimus. If the trough plasma concentration cannot meet the target concentration, it can be increased by adding Wuzhi capsules, ketoconazole, Hexin Shuang or cimetidine. If the trough plasma concentration of tacrolimus is >10 ng/mL, the dose of tacrolimus shall be reduced, and the trough concentration shall be rechecked after one week to adjust the target concentration. After treatment, the increase of SCr ≥ 30% of the basic value and eGFR < 60mL/min.1.73m^2 (excluding non-drug factors) shall prompt tacrolimus dose reduction by 50%. If the concentration still fails to reach the target after tacrolimus dose adjustment for one month, the subject shall withdraw from the trial.

5.2.3.2 If the trough plasma concentration of tacrolimus in visit 7 is ≥ 5 ng/mL, the dose of tacrolimus needs to be reduced by 20%-30%, and the trough concentration shall be rechecked in visit 8. If the trough concentration of tacrolimus is still ≥ 5 ng/mL in visit 8, it is necessary to continue to reduce the dose of tacrolimus by 20%-30%, and recheck this concentration in visit 9.

5.2.4 Dose adjustment of mycophenolate mofetil (MMF)

5.2.4.1 Adjust the dose according to the plasma concentration of mycophenolic acid. If MPA-AUC fails to meet the target, increase the oral dose of mycophenolate mofetil, but the maximum dose shall not exceed 1.0g each time, BID. Recheck MPA-AUC after 1 week and adjust the drug administration to meet the target concentration. If the patient has blood neutrophils less than 1300 per μL or total leukocytes less than 3000 per μL, diarrhea or severe infection, the dose of mycophenolate mofetil needs to be reduced by 25%. If the side effect persists, it shall be adjusted to half of the original dose. If the case of mycophenolate mofetil dose adjustment, the plasma concentration shall be measured according to the judgment of the investigators. If the concentration still fails to reach the target after mycophenolate mofetil dose adjustment for one month, the subject shall withdraw from the trial.

5.2.4.2 If MPA-AUC is ≥ 40 ng/mL in visit 6, the dose of mycophenolate mofetil needs to be reduced by 20%-30%. After that, retest is no longer required.

5.3 concomitant medication

- Statins are allowed for use during this research, which can be increased or decreased according to the blood lipid status.
- Various anticoagulant drugs are allowed for use during this research.
- Immunosuppressants other than the test drug are prohibited for use.
- Plasma exchange and biological agents are prohibited for treatment.
- IVIG can be used during this research, but the reason needs to be recorded.
- When hypertension occurs during treatment, angiotensin converting enzyme inhibitor (ACEi) or angiotensin II receptor blocker (ARB) cannot be used, but dihydropyridine calcium antagonists, β- receptor blocker, diuretic and/or α-receptor blocker can be used for control, with a target blood pressure of: <120/80 mmHg.
- Liver protection drugs are allowed for use during this research.
- If blood glucose rises during treatment, the blood glucose shall be actively controlled with hypoglycemic drugs.
- Various Chinese herbal medicines are prohibited for the treatment of kidney diseases during this research.
- The investigators shall ask the subjects to bring all the drugs they are taking at the time of follow-up, so as to check the concomitant medication for the subjects. For the other drugs or treatments that must be continued for the complicated diseases, the drug name (or other treatment name), dosage, times of use and time must be recorded on the Clinical Study Report for analysis and reporting during summary.

5.4 Management of test drugs

5.4.1 Distribution and storage of test drugs

The distribution and management of test drugs shall be carried out according to the steps described in the Protocol. According to the requirements of registration regulations, only the subjects enrolled in the trial can get the test drugs; and only authorized personnel (drug administrators) in each research center can provide and keep the test drugs. All test drugs shall be stored in a safe place, and only a few personnel related to the test are allowed to get in and out for the drugs. The authorized test personnel shall ensure that the amount of the test drugs is consistent with that on the test drug requisition.

All the test drugs shall be locked and stored in a safe, dark and dry place before distribution. The storage temperature shall be below 25°C and 30°C respectively for tacrolimus and mycophenolate mofetil. Each research center shall have a registration form for drug reception and drug distribution, which shall always be kept by a person (drug administrator) designated by the research center. The drug administrator shall ensure that the drugs are intact when they are distributed to the subjects, and the remaining period of validity shall be at least 3 months.

5.4.2 Count of test drugs

The drug administrator has the responsibility to ensure that the quantity of drugs is consistent with the registered quantity, and has the responsibility to keep the drug registration records. According to the requirements of registration laws and regulations, the drug administrator shall keep the drug inventory and verification records throughout the research process, including the records of the quantity of test drugs received from Hangzhou Zhongmeihuadong Pharmaceutical Co., Ltd., the quantity of test drugs stored and/or distributed, and the quantity of drugs returned by the subjects to the research center.

When the test drugs arrive at each research center, the drug administrator shall sign on the drug reception form to confirm the receipt of test drugs for trial use. The form includes but is not limited to: drug name, batch number, quantity, receiving date and drug inspection report.

The drug administrator shall record all the distributions on the distribution checklist. At the end of this research, the quantity of the test drugs on the checklist shall be consistent with the quantity sent by the Sponsor, the quantity of drugs kept in the center, the quantity sent to the subjects and the quantity returned. At the same time, a reasonable explanation shall be given for any discrepancy. During this research, the drugs shall be recycled in stages, and after this research, all empty boxes for used drugs and unopened drugs shall be returned to the Sponsor, with signatures on the return drug form. The recovered drugs shall be destroyed together in the factory of the Sponsor. The destruction of drugs is carried out by authorized special personnel who shall have a certain sense of prevention against the possible dangers in drug destruction. At the same time, they shall not begin such operation without written authorization from the Sponsor. The whole process of destroying drugs will be completely recorded and archived.

5.4.3 Packages and labels of test drugs

The test drugs are packaged by Hangzhou Zhongmeihuadong Pharmaceutical Co., Ltd. according to the relevant laws and regulations, and the labels of the test drugs comply with the requirements of relevant laws and regulations.

5.5 Medication adherence

Distribute the tacrolimus capsules or mycophenolate mofetil dispersible tablets according to the Dosage and Administration specified in the Protocol. The quantity of drugs distributed before administration, the quantity of drugs used and the quantity returned by each subject shall be recorded in detail. Including the following content:

A: Name, date and quantity of drugs distributed to subjects

B: Name, date and quantity of drugs returned by subjects

The clinical research associate shall check the inventory drugs, returned drugs and drug distribution records of the research center on a regular basis. At the end of this research, the remaining drugs, returned drugs and their packages shall be counted and recovered.

The medication adherence is calculated as follows, and shall be ranging from 80% to 120%:

$$\text{adherence} = \frac{\text{actual administration}}{\text{planned administration}} \times 100\%$$

At each follow-up, the investigators shall emphasize the importance of medication adherence to the subjects.

6. Test process and steps

The selected patients are randomly divided into two groups. Patients in FK506 Group receive tacrolimus capsules and steroids for one year; and patients in MMF Group receive mycophenolate mofetil dispersible tablets and steroids for one year. Both groups follow the "clinical trial flow chart" in operation.

7. Evaluation of efficacy and safety

7.1 Primary efficacy outcomes

1-year relapse-free survival

7.2 Efficacy evaluation

7.2.1 Primary observation outcomes: 1-year relapse-free survival

7.2.2 Secondary observation outcomes:

(1) Frequency of relapses; the time of the first relapse after enrollment; relapse-free **survival** in the first six months after enrollment;

(2) Renal function: Serum creatinine and glomerular filtration rate (eGFR, see Appendix 1 for the calculation method)

(3) Cumulative steroid dosage: The total amount of steroids taken during this research and the total amount of steroids in the first half of the year

(4) Others: Blood pressure (see Appendix 2 for details), height, weight, blood lipid, hemoglobin, blood albumin, urinary protein/creatinine (morning urine), etc.

(5) Adverse reaction

(6) 24hr urinary protein quantification (applicable to subjects > 3 years old)

7.2.3 Efficacy criteria

- Complete remission (CR): Completely normal in blood biochemistry test and urine examination;
- Partial remission (PR): Urinary protein positive < (+++);
- No remission (NR): Urinary protein positive ≥ (+++).

7.2.4 Relapse criteria

For 3 consecutive days, morning urinary protein changes from negative to (+++) or (++++), or 24hr urinary protein quantification ≥ 50 mg/kg, or urinary protein/creatinine (mg/mg) ≥2.0.

7.3 Safety evaluation

Changes of vital signs: Respiration and heart rate.

Various indexes in laboratory examination: Blood and urine routine examination, liver function, renal function, ECG, chest X-ray examination.

Clinically reported adverse events and adverse reactions.

8. Reporting adverse events

8.1 Definition of adverse events

Adverse event (AE) refers to the adverse medical conditions or deterioration of the original medical condition after or during drug treatment, regardless of whether there is a causal relationship with the drug. Adverse medical conditions can be symptoms (such as nausea, and chest pain), signs (such as tachycardia, and hepatomegaly), or abnormal examination results (such as laboratory examination, and ECG). In clinical researches, AE can occur at any time, including the adverse medical conditions in the screening period, even if no study treatment is received.

The term AE includes severe ones and non-severe ones.

8.1.1 Concerned averse reactions of test drug FK506:

Adverse reactions associated with immunosuppressive drugs are often difficult to be determined due to the underlying diseases of patients and combined use of multiple drugs at the same time. The following adverse reactions are reversible, or can be reduced or eliminated after dose reduction. Compared with intravenous administration, oral administration has a lower incidence of adverse reactions.

Cardiac abnormalities

Common: Ischemic coronary artery disease, and tachycardia; Unusual: Ventricular arrhythmia and cardiac arrest, heart failure, cardiomyopathy, ventricular hypertrophy, supraventricular arrhythmia, palpitation, abnormal ECG, abnormal heart rate and pulse; Rare: Pericardial effusion; Very rare: Abnormal echocardiography.

Blood and lymphatic system abnormalities

Common: Anemia, leucopenia, thrombocytopenia, leucocytosis and abnormal erythrocyte analysis; Unusual: Abnormal coagulation function, pancytopenia, and neutropenia; Rare: Thrombotic thrombocytopenic purpura, and low prothrombin; Unknown: Pure red cell aplasia, agranulocytosis, and hemolytic anemia.

Neurologic abnormalities

Very common: Tremor, and headache; Common: Epilepsy, disturbance of consciousness, paresthesia and dullness, peripheral neuropathy, vertigo, dysgraphia and nervous system disorders; Unusual: Coma, central nervous system hemorrhage and cerebrovascular accident, paralysis and local anesthesia, encephalopathy, abnormal language function and cognitive impairment; Rare: Hypertonia; Very rare: Myasthenia.

Eye and ear abnormalities

Common: Blurred vision, photophobia, and eye discomfort; Unusual: Cataract; Rare: blindness, abnormal ear and labyrinth; Common: Tinnitus; Unusual: Hearing retardation; Rare: Neurogenic deafness; Very rare: Hearing loss.

Respiratory, chest and mediastinum abnormalities

Common: Dyspnea, abnormal lung parenchyma, pleural effusion, pharyngitis, cough, nasal congestion and inflammation; Unusual: Respiratory failure, respiratory diseases, and asthma; Rare: Acute respiratory distress syndrome; Unknown: Interstitial lung disease, and pulmonary embolism.

Gastrointestinal abnormalities

Very common: Diarrhea, and nausea; Common: Gastrointestinal inflammation, gastrointestinal ulcer and perforation, gastrointestinal bleeding, stomatitis and ulcer, ascites, vomiting, abdominal pain, dyspepsia, constipation, abdominal distention, loose stools and other gastrointestinal symptoms and signs; Unusual: Paralytic intestinal obstruction, peritonitis, acute and chronic pancreatitis, elevated blood amylase, gastroesophageal reflux and abnormal gastric emptying; Rare: Intestinal obstruction, and pancreatic pseudocyst.

Renal and urinary abnormalities

Very common: Kidney injury; Common: Renal failure, oliguria, tubular necrosis, toxic nephropathy, and bladder and urethral symptoms; Unusual: Anuria, and hemolytic uremic syndrome; Very rare: Kidney disease, and hemorrhagic cystitis; Unknown: Membranous glomerulonephritis, hydronephrosis, nephromegaly, and nephrotic syndrome.

Skin and subcutaneous tissue abnormalities

Very common: Pruritus, rash, hair loss, acne, and hyperhidrosis; Unusual: Dermatitis, and photosensitivity; Rare: Toxic epidermal necrolysis (Lyell's syndrome); Very rare: Stevens-Johnson syndrome; Unknown: Melanoderma.

Skeletal muscle and connective tissue abnormalities

Common: Joint pain, muscle spasm, limb pain, and back pain; Unusual: Joint disorder; Unknown: Bone defect, kyphosis, patellar pain syndrome, and rhabdomyolysis.

Endocrine abnormalities

Rare: Hirsutism; Unknown: Diabetes ketoacidosis.

Metabolic and nutritional abnormalities, and other electrolyte abnormalities

Very common: Hyperglycemia, diabetes, and hyperkalemia; Common: Hypomagnesemia, hypophosphatemia, hypokalemia, hypocalcemia, hyponatremia, fluid retention, hyperuricemia, decreased appetite, anorexia, metabolic acidosis, hyperlipidemia, hypercholesterolemia, and hypertriglyceridemia. Other electrolyte abnormalities: Unusual: Dehydration, hypoproteinemia, hyperphosphatemia and hypoglycemia.

Infection and invasion

Like other potent immunosuppressants, tacrolimus administration shows an increased risk of infection (viruses, bacteria, fungi and protozoa). Existing infections may be aggravated, and the systemic and local infections may occur. Patients treated with immunosuppressants, including tacrolimus, have been reported to have BK virus-related nephropathy and JC virus-related progressive multifocal leukoencephalopathy (PML).

Injury, intoxication and operation complications

Benign, malignant and unclear tumors (including cysts and polyps). Patients treated with immunosuppressants have an increased risk of malignant tumors. Benign and malignant tumors, including EBV-related lymphoid hyperplasia and skin malignancies, have been reported to have possible association with the tacrolimus treatment.

Vascular abnormalities

Very common: Hypertension; Common: Bleeding, thrombus and local ischemia, peripheral vascular abnormalities, and hypotension; Unusual: infarction, limb deep vein thrombosis, and shock.

General abnormalities and administration site symptoms

Common: Weakness, fever, edema, pain and discomfort, elevated blood alkaline phosphatase, weight gain, and body temperature sensation disorder; Unusual: Multiple-organ failure, influenza like symptoms, temperature intolerance, chest compression, tension, paresthesia, elevated blood lactate dehydrogenase and weight loss; Rare: Thirst, fall, reduced activity, and ulcer; Very rare: Increased adipose tissue; Unknown: Death or sudden cardiac death, and abnormal gait.

Immune system abnormalities and hepatobiliary abnormalities

Allergic and anaphylactic reactions are observed in patients treated with tacrolimus.

Common: Abnormal liver function, cholestasis and jaundice, hepatocyte injury, hepatitis and cholangitis; Rare: Hepatic artery thrombosis, and venous occlusive liver disease; Very rare: Liver failure, and biliary stricture;

Reproductive system and breast abnormalities

Unususal: Dysmenorrhea and uterine bleeding; Unknown: Benign prostatic hyperplasia, and mental disorders; Very common: Insomnia; Common: Anxiety, confusion and disorientation, despondency, depression, emotional instability and disorder, nightmares, hallucinations and mental disorders; Unusual: Mental abnormality; Unknown: Aggressive behavior, and agitation.

8.1.2 Concecrned adverse reactions of test drug MMF:

Infection

It refers to the local tissue inflammatory reaction and systemic inflammatory reaction caused by bacteria, viruses, fungi, parasites and other pathogens invading the human body. Serious life-threatening infections, such as meningitis and infective endocarditis, are occasionally reported, and some evidences show that certain types of infections, such as conjugated and atypical waste bacilli infections, lay have a high incidence

Gastrointestinal adverse reactions

Gastrointestinal adverse reactions include nausea, vomiting, dyspepsia, abdominal pain, constipation, upper and lower gastrointestinal discomfort, gastrointestinal inflammation, gastroduodenal ulcer, gastrointestinal bleeding and perforation, etc. Colitis (sometimes caused by cytomegalovirus), pancreatitis, and individual cases of intestinal villus atrophy.

Blood system and immune system

In patients treated with this product in combination with other immunosuppressants, pure red cell aplasia (PRCA) and hypogammaglobulinemia have been reported.

Congenital diseases

Congenital malformations, including ear, face, heart and nervous system abnormalities, have been reported in the children of mothers treated with this product and other immunosuppressants during pregnancy. There are also reports of spontaneous abortion in early pregnancy.

Pregnancy, puerperium and perinatal periods

Spontaneous abortion has been reported in patients (mainly in early pregnancy) exposed to mycophenolate mofetil.

Malignant tumors

Patients taking this product as part of immunosuppression for immunosuppressive regimen, including the patients with combined use of drugs, have an increased risk of lymphoma and malignant tumors, especially skin tumors. In a controlled clinical trial for patients with a follow-up of at least 1 year after kidney, heart and liver transplantation, lymphoproliferative disease or lymphoma develop in 0.4% to 1% of the patients treated with this product (2g or 3g daily) in combination with other immunosuppressants. Non-melanoma skin cancer develops in 1.6% to 3.2% of the patients; 0.7% to 2.1% of patients have other types of malignant tumors. In the 3-year safety data of kidney and heart transplantation patients, the incidence rate of malignant tumors shows no unexpected difference with that in the 1-year data. All the patients with liver transplantation are followed up for more than 1 year and less than 3 years. In the controlled trial of refractory renal transplantation, the incidence of lymphoma is 3.9% with an average follow-up of 42 months.

Children (aged 3 months to 18 years)

In a clinical study of 100 pediatric patients aged 3 months to 18 years, the type and frequency of adverse reactions after oral administration of 600 mg/m^2 mycophenolate mofetil (BID) are generally similar to those observed in adult patients with daily administration of 1g mycophenolate mofetil (BID). However, as compared with the adult patients, the following treatment-related adverse events with a frequency of 10% are more common in the pediatric population, especially in children under the age of 6 years, including diarrhea, leukopenia, sepsis, infection and anemia.

Elderly patients (≥ 65 years old)

As compared with young people, the elderly, especially the elderly patients receiving this product as part of the combined immunosuppressive regimen, have an increased risk of some infections (including cytomegalovirus tissue invasion disease), possible gastrointestinal bleeding and pulmonary edema.

Toxicity criteria

Based on the revised standard of the World Health Organization (WHO) on the grading of acute and subacute toxicity of drugs.

8.2 Definition of serious adverse event

Serious adverse event (SAE) refers to the AE occurring at any stage of this research (i.e. screening period, follow-up period) and complying with one or more of the following criteria:

- Causing death
- Immediate life-threatening
- Requiring hospitalization or extension of the original length of stay

- Causing sustained or significant deformity/disability, or seriously impairing the ability to complete normal life functions
- Congenital malformation or defect at birth
- An important medical event that may cause harm to the patient or may require medical intervention to prevent one of the above outcomes

8.3 Records of adverse events

The information of AEs to be collected in this research includes the following aspects:

- Endpoint event
- Adverse events related to the test drugs (according to the judgment of the investigators)
- Serious adverse event

Cycle of adverse event collection

The collection of AEs and adverse drug reactions (DAEs) of concern will be carried out throughout this research, from randomization to site close-out visit (SCV, included).

SAE records will be carried out throughout this research, from the time of signing the Informed Consent Form to SCV (included).

Follow-up of unresolved adverse events

If any AE, DAE and SAE of concern remain unresolved at the patient's last study visit, the investigators shall follow up these events in accordance with the medical instructions as required for medical needs in this research, but it is not necessary to record further in the electronic case report form (eCRF). If deemed necessary, the Sponsor reserves the right to obtain the additional information on unresolved AE/SAE of concern.

Variables

For each non-serious AE and DAE of concern, the following variables will be collected:

- AE (verbatim record);
- AE start and end dates;
- Maximum intensity;
- Whether the AE is serious;
- The investigators' assessment of the causal relationship with the test drug (yes or no);
- Measures and outcomes taken for the test drug

In addition, the following variables will be collected for SAE:

- Date when AE meets SAE criteria;
- Date when the investigators find SAE;
- Reasons for classifying AE as SAE;
- Date of admission:
- Date of discharge:

- Possible causes of death
- Date of death:
- Autopsy performed (if any);
- Autopsy results
- AE description
- Assessment of causality with this research procedure;
- Assessment of causality with the other drugs

It is important to distinguish serious AE from severe AE. Severity is the index of intensity evaluation, while seriousness is defined according to the criteria in Section 8.2. AE with severe intensity is not necessarily serious. For example, nausea for several hours may be considered as severe nausea, but not SAE. On the other hand, a stroke that results in only a limited degree of disability may be considered as a mild stroke, but should be considered as SAE.

The definition of intensity classification is as follows:

1. Mild (signs or symptoms are felt but easily tolerated)
2. Moderate (the degree of discomfort is enough to interfere with normal activities)
3. Severe (resulting in disability and inability to perform normal activities)

Collection of causalities

The investigators will assess the causalities between the test drugs and each case of AE, and answer the following questions with "Absolutely related, Probably related, Possibly related, Possibly unrelated, and Unrelated".

"Do you think there is a reasonable possibility that the event is caused by the test drug?"

For SAE, the causalities between the event and other drugs and research procedures also need to be evaluated. Note: for SAE related to any research procedure, causality should be "possibly related".

Adverse events based on signs and symptoms

All concerned AEs (see sections 8.1.1 and 8.1.2), including those spontaneously reported by the patients, those reported in response to the open-ended question by the investigators, "Have you had any health problems since the last visit/inquiry?", or those found through observation will be all collected and recorded in eCRF. In the collection of AE information, the list of signs and symptoms shall be preferred as the record of diagnosis (if possible). However, if the diagnosis is known and there are other signs or symptoms not usually present in the diagnosis, the diagnosis and each other sign or symptom shall be recorded separately.

Adverse events based on examination and inspection

During this research, the laboratory safety assessment will not be carried out routinely (except at baseline), but can be carried out at the discretion of the investigators. When an AE, DAE, SAE or potential endpoint of concern is involved, different assessments may be required, including local laboratory assessment, and appropriate collection is needed before entry into eCRF.

The examination results of vital signs mandated by the Protocol shall be summarized in the clinical study report (CSR). Therefore, when the laboratory test values and protocol-mandated vital signs evaluation by the investigators show that the results are worse than the baseline level and meet the AE criteria or when they act as are the reasons for discontinuing the test drug treatment, such situations will be reported as AE.

If the deterioration of laboratory test values/vital signs is related to clinical signs and symptoms, the signs and symptoms will be reported as AE and relevant laboratory results/vital signs will be used as supplementary information. Investigators shall use clinical terms rather than laboratory terms (e.g. renal failure rather than elevated creatinine) as much as possible in the report. If no clinical signs or symptoms are present, the clinically relevant deterioration of non-mandatory parameters shall be reported as AE and SAE.

8.4 Report of serious adverse events

All SAEs must be reported, regardless of whether they are considered to have causalities with the test drugs or study procedure. All SAEs will be recorded in eCRF.

In case of any SAE during this research, the investigators or other staff of the center shall notify the relevant Sponsor representative immediately or no later than 24 hours after learning of the event.

The designated Sponsor representative shall work with the investigators to ensure that all necessary information is provided to the Sponsor's patient safety data entry center within 1 working day after the first receipt of fatal and life-threatening events and 5 working days after the first receipt of all other SAEs.

For fatal and life-threatening AEs, if important or relevant information is missing, immediate active follow-up is required. The investigators or other staff of the center shall notify the Sponsor representative of any follow-up information about the previously reported SAE immediately or no later than 24 hours after receiving the information.

If the Web-based data collection (WBDC) system is not available, the investigators or other staff of the center shall report SAE to the appropriate Sponsor representative by telephone, noting that the same reporting window (24 hours) is still applicable.

If initial or subsequent reporting is made through the means other than WBDC system, the investigators must finalize any SAE information into the eCRF through WBDC system as soon as this system is enabled again.

9. Quality control and assurance of the trial

9.1 Quality control measures in laboratory

9.1.1 Quality control before analysis

- Each center appoints a technician from the Clinical Laboratory Department to be responsible for the laboratory inspection related matters for this project. The appointed technician shall test the samples in time according to the requirements, and strictly abide by the rules of keeping and taking the samples on time.
- Project training shall be completed before trial initiation.

9.1.2 Quality assurance during analysis

- In the middle of the project (progress of about 50%), a comparison test of the laboratory observation indicators is conducted (5 samples for each project, and see Appendix 3 for the comparison criteria).

9.1.3 Quality assurance after analysis

- Each center can keep the test results for a long time;
- According to the requirements of this project, the remaining blood and urine samples (serum for blood samples, and the residual samples of 24h urine protein quantitation for the urine samples) shall be stored in the refrigerator of -80℃, and the preserved samples shall be transported regularly (within 3 months) to the Children's Hospital, Zhejiang University School of Medicine for centralized testing.
- Before the end of the project (progress of about 90%), another comparison test of the laboratory observation indicators is conducted (5 samples for each project, and see Appendix 3 for the comparison criteria).

9.2 Quality control and assurance in the trial process

(1) All hospitals participating in the clinical trial shall adopt standard operating procedures to ensure the implementation of quality control and quality assurance systems for the clinical trial.

(2) In order to ensure the quality of the multicenter trial, the principals of the participating hospitals shall discuss and prepare the clinical study plan together before the formal trial starts. The relevant medical personnel participating in the trial are trained in GCP at the same time.

(3) The hospitals participating in the trial shall, in accordance with the uniform clinical research protocol, start and end the trial at the same time as possible.

(4) All hospitals participating in the trial are required to manage the test drugs with the same procedures, including drug receiving, storage, distribution and recycling.

(5) All observed results and abnormal findings in the clinical trial shall be carefully verified and recorded in time to ensure the reliability of the data. All instruments, equipment, reagents and reference materials used in various test items in the clinical trial shall have strict quality

standards and ensure that they work under normal conditions. The recording and transfer of clinical data must be in the charge of experienced physicians and supervised or checked by special personnel to ensure the scientificity and accuracy of the data. Various conclusions of the clinical trial must be derived from the original data. All the original data and CRF shall be kept for 5 years.

(6) The physicians in charge of the trial shall fill in the case report form (CRF) in a complete, detail and accurate manner. After each subject completes the trial, all data related to the trial shall be centrally managed and analyzed before submission to the superior physician for signature, and then submitted or kept according to the specified procedures.

(7) Procedures shall be established for data keeping, data transmission and data query. The data to be kept include: medical records, imaging data, CRF, drug use registration form, subject code form, serious adverse event report form, GCP form to be filled by each hospital, follow-up report form and various original medical documents of the subjects. The data to be transmitted include: total randomization form of subjects, CRF, serious adverse event report form, as well as data and materials to be used for summarization. The drug regulatory authorities, the Sponsor and its entrusted supervisors, and the relevant leaders of the hospitals have the right to consult the relevant test data and original records of each hospital with the consent of the professor in charge of the trial. Finally in the sorting of all the test reports, the team leader's unit and the person in overall charge can consult or access the data and original records of all subjects with consent.

(8) In the summarization and analysis of the clinical trial results, standardized statistical analysis methods must be adopted, and personnel familiar with biostatistics should be invited for participation.

(9) In order to ensure the reliability and integrity of the test data, the person in overall charge of the team leader's unit, the Sponsor and its CRO supervisor shall regularly conduct systematic supervision on the clinical hospitals, to determine whether the implementation of the trial is consistent with the Protocol and whether the reported data is consistent with the records of the clinically participating units. A visit report shall be written for each supervision and visit.

(10) In order to better supervise and inspect the trial, the research and development unit and the leading unit of this clinical trial shall carry out irregular inspection on each clinical trial hospital. It aims to determine whether the implementation of the trial, data recording and analysis are consistent with the requirements specified in the Protocol, drug clinical trial management specifications and regulations. An report shall be written after the audit.

(11) The test drug keeper must carefully keep the test drugs according to the drug storage conditions proposed by the Sponsor.

(12) The responsible unit shall organize experts to train the investigators on the Protocol before the clinical trial, and conduct the consistency test of all quantitative standards.

(13) During the clinical trial, the supervisor of CRO company signed with the Sponsor will regularly visit the research centers to ensure that all content of the Protocol is strictly observed, and check the original data to ensure that the information filled in CRF is true, complete and correct.

9.3 Measures to improve subject compliance

To ensure that the enrolled patients can positively complete the trial, importance must be attached to the measures of subject compliance.

(1) For each enrolled patient, it is necessary to clarify the possible benefits and risks of the test drugs to them.

(2) The Informed Consent Form must be signed by each enrolled patient or his/her legal representative and guardian.

(3) Each enrolled patient must be required to use the test drug or control drug in a sufficient amount and an effective way each time.

(4) If it is found that any subject refuses to use drugs (including the control drug) in study, the ideological work shall be actively done for the subject and the guardian. In addition, it must be immediately reported to the competent physician for treatment.

9.4 Quality control and quality assurance systems

The investigator shall perform their respective responsibilities, strictly follow the clinical trial protocol and adopt standard operating procedures to ensure the implementation of quality control and quality assurance systems for the clinical trial.

The investigators shall follow the principles of GCP in the whole process of this research, and shall record all the content in CRF in a truthful, detailed and careful manner. All the observations and findings in the clinical trial shall be verified to ensure the reliability of the data. All laboratory tests shall strictly follow the standard operation procedures (SOP) to ensure that all provisions in the Protocol are strictly followed and the research data are filled in correctly.

10. Data management

10.1 Data management system

The electronic data management system - clinical research integration platform is adopted in this research to collect data and deal with relevant questions. The investigators shall ensure that the data are recorded in the electronic case report form (eCRF) in a timely manner in accordance with the

provisions of the Protocol and the instructions provided. The research staff will receive training and be responsible for inputting the data specified in the Protocol into the electronic data management system according to the eCRF instructions.

10.2 Data record

In accordance with the guidelines for completing the case report form, the investigators shall ensure the accuracy, completeness and timeliness of the recorded data, and ensure the response to data queries. The investigators shall verify the data that are significantly higher than or beyond the range of clinical acceptance, and make necessary explanations.

In modifying the data during filling in, the investigators shall indicate the reason for modification in the popped-up eCRF dialog box. Any modification by investigators will leave traces to ensure the authenticity of the data. For the questions in the CRF, the data administrator will generate a data query form and send a query to the investigators through the electronic data management system. The investigators shall give and return the answer as soon as possible.

10.3 Data supervision

The clinical supervisor shall regularly review the CRF and timely feed back the problems found to the person in charge of this research, until signature for confirmation upon compliance with the requirements.

11.Statistical analysis

11.1 Analysis data set

Full analysis set (FAS): It refers to the set of eligible cases and drop-off cases. The cases are required to have evaluation data after at least one treatment, but excluding the eliminated cases. Per-protocol set (PPS): It refers to the set of cases that meet the inclusion criteria, do not meet the exclusion criteria and complete the treatment plan, that is, the analysis of cases that meet the trial protocol, have good compliance and complete the specified content in CRF (PP analysis).

Safety set (SS): It refers to the actual data with safety index records after receipt of at least one treatment. The missed safety values shall not be carried forward. It includes some eliminated cases that can be evaluated, such as those whose age exceeds the inclusion criteria, but excludes those whose safety judgment cannot be made due to the use of banned drugs. For the incidence of adverse reactions, the number of cases in the safety set is used as the denominator.

11.2 Statistical method

11.2.1 General statistical methods

Two-sided test is adopted for all statistical tests. If the P value is less than 0.05, the tested difference will be considered "statistically significant" (unless otherwise specified).

Descriptive analysis: Sort out and summarize a large number of data obtained from the survey to find out the internal law of these data - centralized trend and decentralized trend. Single factor analysis is mainly carried out with the help of statistics represented by various data, such as mean and percentage.

11.2.2 Statistical inference method

- The description of quantitative indicators includes the calculation of the mean, standard deviation, median, minimum value, maximum value, lower quartile (Q1), upper quartile (Q3), as well as the number and percentage of various types of classification indicators.
- In the comparison of the general conditions between the two groups, appropriate methods shall be adopted for analysis according to the type of indicators. The group t-test or Wilcoxon rank sum test is used for the comparison of quantitative data between two groups; Chi-square test or exact probability method is used for classification data; Wilcoxon rank sum test or CMH test is used for hierarchical data.
- The report is mainly expressed in tables: The tables are self-evident, that is, they have table titles, table notes and number of cases.

11.2.3 Statistical software

SAS 9.4 software is adopted for statistical analysis.

11.3 Content of statistical analysis

11.3.1 Basic value equilibrium analysis

The demographic characteristics, general conditions and baseline conditions (basic value indicators) of the two groups are analyzed to measure the comparability of the two groups. Group t-test or Wilcoxon rank sum test is used for measurement data; Wilcoxon rank sum test is used for classification or grade data; χ^2 test or Fisher test is used for classification data.

11.3.2 Effectiveness analysis

All effectiveness analysis/global indicators will be based on ITT principles, and FAS is adopted, for only the adjudicated endpoint events. The investigators are required to report all the potential endpoint events in eCRF.

11.3.3 Primary efficacy analysis

Logrank test is used for the analysis of relapse-free survival rate which is then used as a covariate for stratification by centers. HR and its 95% confidence interval (CI) are calculated with Cox proportional hazard model to estimate the efficacy, the confidence interval is applied to verify the

superiority of the experimental group and the control group, with a cut-off value of 1 (risk ratio). The model incorporates the same covariate as the Logrank test for fitting, and the definition method of covariates is also the same as that in the Logrank test. The Kaplan-Meier curve of relapse-free survival is drawn by treatment groups. The 1-year relapse-free survival rate and its confidence interval are then calculated.

11.3.4 Secondary efficacy analysis

Secondary outcomes (renal function; cumulative steroid dosage; blood pressure; height; body weight; blood lipids; hemoglobin; serum albumin; urinary protein/creatinine (morning urine); 24hr urinary protein quantification (applicable to subjects > 3 years old); frequency of relapses; the time of the first relapse after enrollment; relapse-free rate in the first six months after enrollment) are described and compared with use of the above general statistical methods, and the adverse reactions are analyzed by descriptive analysis.

11.3.5 Other efficacy analysis

(1) Case distribution: Different data sets in each group, case distribution in each center, comparison of total drop-off rate, detailed list of termination reasons, and descriptive analysis.

(2) Compliance analysis: Compare whether the two groups of patients take the test drugs on time and in quantity, whether they take the drugs and foods prohibited in the Protocol, and conduct the same descriptive analysis as well as χ^2 test or Fisher test for analysis.

(3) The incidence of drug withdrawal or drug change due to adverse reactions in the clinical trial shall be analyzed with χ^2 test or Fisher test. The efficacy in the long-term follow-up after the trial is evaluated by descriptive analysis and χ^2 test or Fisher test.

11.3.6 Safety analysis

Safety analysis mainly adopts safety set. All adverse events are coded by MedDRA 20.0 to describe the total number and incidence of adverse events (including serious adverse events), and the frequency table is used to describe the frequency, severity of each adverse event and its relationship with the treatment. Adverse events relevant (related, probably related, and possibly relayed) to this research treatment are described and listed separately. Other safety indicators will also be described and summarized. The laboratory changes with clinical significance will be counted as AE.

12. Ethical principles

12.1 Ethical review

The clinical trial protocol shall be submitted to the Ethics Committee for approval before implementation. If this Protocol is to be revised during the clinical trial implementation, it shall be submitted to the Ethics Committee for approval again before implementation. If important but new information concerning the test drugs is found, the Informed Consent Form must be revised in writing and submitted to the Ethics Committee for approval before signing by the subjects again.

12.2 Benefits and risks

This test drug in this research is already on the market, and no serious adverse reactions have been reported in clinical application. In case of possible side effects, the investigators shall take the corresponding medical countermeasures according to the specific conditions of the patients and shall have the right to suspend the clinical trial based on their own judgment.

The research could expand the application scope of the marketed drug which may be used for the other patients with similar conditions. Subjects will receive good medical care during this research.

12.3 Informed consent process

The investigators must explain to the subjects about the details of the clinical trial, including its purpose, procedure, possible benefits and risks, the rights and obligations of the subjects, etc., so that the subjects can fully understand it and have plenty of time for consideration. The clinical trial can be started after the subjects get satisfactory answers to the questions raised, and sign the Informed Consent Form upon agreement. Whenever a patient signs the Informed Consent Form, the physician shall leave his/her contact number to the patient so that the patient can find him/her at any time if any condition change occurs. Informed Consent Form shall be submitted to the Ethics Committee for review.

12.4 Medical treatment and protection of subjects

The investigators are responsible for the medical treatment of the subjects, making medical decisions related to the clinical trial, and ensuring that the subjects could receive appropriate treatment in case of adverse events during the trial.

12.5 Protection of subject privacy

Only the investigators and supervisors involved in the clinical trial may have access to the personal medical records of the subjects. During data processing, the method of "data anonymity" is adopted, and the information that can identify the individual identity of the subjects will be omitted.

13. Ethical requirements

The clinical trial must be conducted in accordance with the Declaration of Helsinki and the relevant clinical trial research norms and regulations in China. The clinical trial can be implemented after the Protocol is approved by the Ethics Committee of the unit in charge of the clinical research.

Before each patient is enrolled in this research, the research physician has the responsibility to completely and comprehensively introduce the purpose, procedure and possible risks of this research to the patient or his/her designated representative in a written form, and let the patients know that they have the right to withdraw from this research at any time. Before enrollment, each patient or his family members must be given a written Informed Consent Form. The research physician has the responsibility to obtain the Informed Consent before each patient is included in this research. The Informed Consent Form shall be kept as a clinical trial document for future reference.

14. Data preservation

The trial protocol and its amendment (signed), Informed Consent Form (format), approval from the Ethics Committee, membership list of the Ethics Committee, original outpatient or inpatient medical record, case report form (filled in, signed and dated), original of adverse event report and electronic data management system database.

15. Expected progress

15.1 Trial start time: This Protocol will be officially implemented after being approved by the Ethics Committee. It is planned to start the clinical trial after the test drugs, research materials, and research funds are in place and the project kick-off meeting.

15.2 Planned enrollment time: Complete the enrollment of subjects within 24 months after the start of the trial.

15.3 The time of clinical trial data collection, statistics and summary is shown in the table below:

**Working contents**	**Plan completion time**
Kick-off meeting	2019.6
Enrollment of the first subject	2019.7
Enrollment of half the subjects	2020.7
Enrollment of the last subject	2021.7
Phased analysis and summary	2020.7
End of the observation	2022.7
Data collection	2019.7 - 2022.7
Statistical analysis	2022.9

Summary, article	2022.12

16. Follow-up and medical measures after the trial

If the subjects have adverse events/reactions during the clinical trial, they must be followed up within 1 month after the treatment to ensure their safety. If the subjects still need therapy for their uncured conditions after the end of the trial, the current standard therapeutic drugs can be used for treatment.

17. References

1. Filler G, Young E, Geier P, Carpenter B, Drukker A, Feber J (2003) Is there really an increase in non-minimal change nephrotic syndrome in children? Am J Kidney Dis 42:1107-1113.
2. Eddy AA, Symons JM (2003) Nephrotic syndrome in childhood. Lancet 362:629-639.
3. Wong W (2007) Idiopathic nephrotic syndrome in New Zealand children, demographic, clinical features, initial management and outcome after twelve-month follow-up: results of a three-year national surveillance study. J Paediatr Child Health 43:337-341.
4. 全国儿童常见肾脏病诊治现状调研工作组 (2014) 我国儿童激素敏感、复发/依赖肾病综合征诊疗现状的多中心研究. 中华儿科杂志 52:194-200.
5. Tarshish P, Tobin JN, Bernstein J, Edelmann CM, Jr. (1997) Prognostic significance of the early course of minimal change nephrotic syndrome: report of the International Study of Kidney Disease in Children. J Am Soc Nephrol 8:769-776.
6. Shaw KT, Ho AM, Raghavan A, Kim J, Jain J, Park J, Sharma S, Rao A, Hogan PG (1995) Immunosuppressive drugs prevent a rapid dephosphorylation of transcription factor NFAT1 in stimulated immune cells. Proc Natl Acad Sci U S A 92:11205-11209.
7. Koefoed-Nielsen PB, Karamperis N, Hojskov C, Poulsen JH, Jorgensen KA (2006) The calcineurin activity profiles of cyclosporin and tacrolimus are different in stable renal transplant patients. Transpl Int 19:821-827.
8. 吴晓明, 夏正坤 (2014) 他克莫司在儿童肾脏疾病中的临床应用及作用机制. 国际儿科学杂志 41:386-389.
9. Li X, Li H, Chen J, He Q, Lv R, Lin W, Li Q, He X, Qu L, Suya W (2008) Tacrolimus as a steroid-sparing agent for adults with steroid-dependent minimal change nephrotic syndrome. Nephrol Dial Transplant 23:1919-1925.
10. Ren H, Shen P, Li X, Pan X, Zhang W, Chen N (2013) Tacrolimus versus cyclophosphamide in steroid-dependent or steroid-resistant focal segmental glomerulosclerosis: a randomized controlled trial. Am J Nephrol 37:84-90.

11. 汤京华, 汤婷, 方莉, 冯曦 (2011) 吗替麦考酚酯治疗难治性原发性肾病综合征疗效观察. 中国药师 14:104-105.

12. 李艳伟, 夏天 (2009) 难治性肾病综合征的治疗及其进展. 医学综述 15:2497-2499.

13. Sepe V, Libetta C, Giuliano MG, Adamo G, Dal Canton A (2008) Mycophenolate mofetil in primary glomerulopathies. Kidney Int 73:154-162.

14. 郝海英, 郑朝霞, 徐岚 (2011) 吗替麦考酚酯联合泼尼松治疗老年难治性肾病综合征的临床分析. 中国医师进修杂志 34:29-31.

15. Briggs WA, Choi MJ, Scheel PJ, Jr. (1998) Successful mycophenolate mofetil treatment of glomerular disease. Am J Kidney Dis 31:213-217.

16. Gellermann J, Weber L, Pape L, Tonshoff B, Hoyer P, Querfeld U, Gesellschaft fur Padiatrische N (2013) Mycophenolate mofetil versus cyclosporin A in children with frequently relapsing nephrotic syndrome. J Am Soc Nephrol 24:1689-1697.

17. KDIGO Board Members (2012) KDIGO Clinical Practice Guideline for Glomerulonephritis. Kidney International 2:139-251.

18. 中华医学会儿科学分会肾脏学组(2017) 激素耐药型肾病综合征诊治循证指南（2016). 中华儿科杂志 55:805-809.

19. 中华医学会儿科学分会肾脏学组(2017) 儿童激素敏感及复发/依赖肾病综合征诊治询证指南（2016）中华儿科杂志 55:729-734.

20. Schwartz GJ, Brion LP, Spitzer A (1987) The use of plasma creatinine concentration for estimating glomerular filtration rate in infants, children, and adolescents. Pediatr Clin North Am 34:571-590.

Appendix 1 Estimation of glomerular filtration rate

Estimation of glomerular filtration rate

1. The glomerular filtration rate (eGFR) is calculated and estimated with Schwartz formula[20]:

eGFR (mL/min/1.73m^2)＝K×height (cm)/Scr (mg/dL), where K is the proportional constant:

K=0.55 (girls over 2 years old, boys 2-12 years old)

K=0.70 (boys over 12 years old)

2. At the same time: Unit conversion for serum creatinine (SCR) value: 1 mg/dL = 88.4 μmol/L

Appendix 2 Blood pressure reference values for children

Boy

Age	Systolic blood pressure, mmHg Height percentile							Diastolic blood pressure, mmHg Height percentile						
	5th	10th	25th	50th	75th	90th	95th	5th	10th	25th	50th	75th	90th	95th
	98	99	101	103	104	106	106	54	54	55	56	57	58	58
2	101	102	104	106	108	109	110	59	59	60	61	62	63	63
3	104	105	107	109	110	112	113	63	63	64	65	66	67	67
4	106	107	109	110	112	114	115	66	67	68	69	70	71	71
5	108	109	110	112	114	115	116	69	70	71	72	73	74	74
6	109	110	112	114	115	117	117	72	72	73	74	75	76	76
7	110	111	113	115	117	118	119	74	74	75	76	77	78	78
8	111	112	114	116	118	119	120	75	76	77	78	79	79	80
9	113	114	116	118	119	121	121	76	77	78	79	80	81	81
10	115	116	117	119	121	122	123	77	78	79	80	81	81	82
11	117	118	119	121	123	124	125	78	78	79	80	81	82	82
12	119	120	122	123	125	127	127	78	79	80	81	82	82	83
13	121	122	124	126	128	129	130	79	79	80	81	82	83	83
14	124	125	127	128	130	132	133	80	80	81	82	83	84	84
15	126	127	129	131	133	134	135	81	81	82	83	84	85	85
16	129	130	132	134	135	137	137	82	83	83	84	85	86	87
17	131	132	134	136	138	139	140	84	85	86	87	87	88	89

Girl

Age	Systolic blood pressure, mmHg							Diastolic blood pressure, mmHg						
	Height percentile							Height percentile						
	5th	10th	25th	50th	75th	90th	95th	5th	10th	25th	50th	75th	90th	95th
1	100	101	102	104	105	106	107	56	57	57	58	59	59	60
2	102	103	104	105	107	108	109	61	62	62	63	64	65	65
3	104	104	105	107	108	109	110	65	66	66	67	68	68	69
4	105	106	107	108	110	111	112	68	68	69	70	71	71	72
5	107	107	108	110	111	112	113	70	71	71	72	73	73	74
6	108	109	110	111	113	114	115	72	72	73	74	74	75	76
7	110	111	112	113	115	116	116	73	74	74	75	76	76	77
8	112	112	114	115	116	118	118	75	75	75	76	77	78	78
9	114	114	115	117	120	121	122	77	77	77	78	79	80	80
10	116	116	117	119	120	121	122	77	77	77	78	79	80	80
11	118	118	119	121	122	123	124	78	78	78	79	80	81	81
12	119	120	121	123	124	125	126	79	79	79	80	81	82	82
13	121	122	123	124	126	127	128	80	80	80	81	82	83	83
14	123	123	125	126	127	129	129	81	81	81	82	83	84	84
15	124	125	126	127	129	130	131	82	82	82	83	84	85	85
16	125	126	127	129	130	131	132	82	82	83	84	85	85	86
17	125	126	127	129	130	131	132	82	83	83	84	85	85	86

The blood pressure at 95th percentile standard[14]

Appendix 3 Comparison and acceptance criteria of laboratory observation indexes

(Compared with the results from the Children's Hospital, Zhejiang University School of Medicine)

Serum index	Acceptance criteria	Urine index	Acceptance criteria
Serum creatinine	±8.8 μmol/L<br>Or ±10%, whichever is larger	Total urinary protein	≤0.2g/L<br>Or ±15%, whichever is larger
Uric acid	±15%	Urinary creatinine	±15%
Urea	±15%		
Albumin	±10%		
Total bilirubin	±20%		
ALT	±20%		
According to the above standards, if the results of 80% of the samples are within the range, such samples shall be deemed "accepted"; if 4 samples in the middle and late period are deemed "accepted", this comparison test shall be deemed "accepted".			

Appendix 4 Parameters and results sample size calculation

Parameters and results in sample size calculation:

2019/4/30 10:57:07 1

Logrank Test Analysis

Numeric Results in Terms of Sample Size when the Test is Two-Sided and T0 is 1

Power	N1	N2	N	Haz Ratio (HR)	Ctrl Prop Surv (S1)	Trt Prop Surv (S2)	Acc-rual Pat'n	Acc-rual Time/ Total Time	Ctrl Loss	Trt Loss	Ctrl to Trt	Trt to Ctrl	Alpha	Beta
0.8009	114	115	229	0.6151	0.5600	0.7000	Equal	2 / 3	0.0000	0.0000	0.0000	0.0000	0.0500	0.1991

References

Lakatos, Edward. 1988. 'Sample Sizes Based on the Log-Rank Statistic in Complex Clinical Trials', Biometrics, Volume 44, March, pages 229-241.
Lakatos, Edward. 2002. 'Designing Complex Group Sequential Survival Trials', Statistics in Medicine, Volume 21, pages 1969-1989.

Report Definitions

Power is the probability of rejecting a false null hypothesis. Power should be close to one.
N1|N2|N are the sample sizes of the control group, treatment group, and both groups, respectively.
E1|E2|E are the number of events in the control group, treatment group, and both groups, respectively.
Hazard Ratio (HR) is the treatment group's hazard rate divided by the control group's hazard rate.
Proportion Surviving is the proportion surviving past time T0.
Accrual Time is the number of time periods (years or months) during which accrual takes place.
Total Time is the total number of time periods in the study. Follow-up time = (Total Time) - (Accrual Time).
Ctrl Loss is the proportion of the control group that is lost (drop out) during a single time period (year or month).
Trt Loss is the proportion of the treatment group that is lost (drop out) during a single time period (year or month).
Ctrl to Trt (drop in) is the proportion of the control group that switch to a group with a hazard rate equal to the treatment group.
Trt to Ctrl (noncompliance) is the proportion of the treatment group that switch to a group with a hazard rate equal to the control group.
Alpha is the probability of rejecting a true null hypothesis. It should be small.
Beta is the probability of accepting a false null hypothesis. It should be small.

Numeric Results in Terms of Events when the Test is Two-Sided and T0 is 1

Power	Ctrl Evts E1	Trt Evts E2	Total Evts E	Haz Ratio (HR)	Ctrl Prop Surv (S1)	Trt Prop Surv (S2)	Acc-rual Pat'n	Acc-rual Time/ Total Time	Ctrl Loss	Trt Loss	Ctrl to Trt	Trt to Ctrl	Alpha	Beta
0.8009	76.6	57.2	133.8	0.6151	0.5600	0.7000	Equal	2 / 3	0.0000	0.0000	0.0000	0.0000	0.0500	0.1991

N1: MMF Group

N2: FK506 Group
