# Supplementary material for: Tacrolimus or Mycophenolate Mofetil for Frequently Relapsing or Steroid-Dependent Nephrotic Syndrome: A Randomized Clinical Trial
Source: JAMA Pediatr. 2025 May 12;179(7):722–9. doi: 10.1001/jamapediatrics.2025.0765 (PMC12070277; doi:10.1001/jamapediatrics.2025.0765)
Supplement: Supplement 2. — Statistical Analysis Plan [file jamapediatr-e250765-s002.pdf]

**AMMENDMENT HISTORY**

1. Page 30 of the Original Statistical Analysis Plan has been updated to include the description “8. Data handling conventions. This study does not present issues with multiplicity testing.”.

2. Remove the following description from section 9.1.3. Primary Efficacy Analysis (Page 30) of the Original Statistical Analysis Plan: “The model incorporates the same covariate as the Logrank test for fitting, and the definition method of covariates is also the same as that in the Logrank test.”.

3. Add 9.1.7 to Page 33 of the Original statistical analysis plan. The subgroup analysis is described as follows:

“9.1.7. Subgroup Analysis

Subgroup analyses will be conducted based on the following baseline information:

（1）Gender;

（2）Age at enrollment: Toddlerhood: under 4 years old, not including 4 years old; Preschool age: 4 years old (including 4 years old) to under 7 years old; School age and above: 7 years old and above (including 7 years old).

（3）Age at onset: Toddlerhood: under 4 years old, not including 4 years old; Preschool age: 4 years old (including 4 years old) to under 7 years old; School age and above: 7 years old and above (including 7 years old).

（4）Duration of disease: less than 1 year, 1 year or more.”.

4. Add 9.1.8 to Page 33 of the Original statistical analysis plan. The sensitivity analysis is described as follows:

“9.1.8 Sensitivity analysis

(1) Sensitivity analysis 1: Based on FAS, the Cox proportional hazards model was used to calculate HR and its 95% confidence interval (CI) to estimate efficacy, with adjusted factors including age, gender, age of onset, and course of kidney disease (less than 1 year, greater than or equal to 1 year).

(2) Sensitivity analysis 2: Based on FAS, the Cox proportional hazards model was used to calculate HR and its 95% confidence interval (CI) to estimate efficacy, with adjusted factors being center, age, gender, age of onset, and course of kidney disease (less than

1 year, greater than or equal to 1 year).
(3) Sensitivity analysis 3: Based on PPS, the Cox proportional hazards model was used to calculate HR and its 95% confidence interval (CI) to estimate efficacy. The adjusted factors were age, gender, age of onset, and duration of kidney disease (less than 1 year, greater than or equal to 1 year).
(4) Sensitivity analysis 4: Based on PPS, the Cox proportional hazards model was used to calculate HR and its 95% confidence interval (CI) to estimate efficacy. The adjusted factors were center, age, gender, age of onset, and course of kidney disease (less than 1 year, greater than or equal to 1 year).”.

**Study of Tacrolimus vs Mycophenolate Mofetil in Pediatric Patients with Frequently Relapsing or Steroid-dependent Nephrotic Syndrome: a Prospective, Randomized, Multicenter, Open-label, and Parallel-arm Study on Efficacy and Safety (the STAMP study)**

# Statistical Analysis Plan

| | |
|---|---|
| **NCT No.:** | NCT04048161 |
| **Protocol No.:** | NSTAMF02 |
| **Protocol version No.:** | V 2.1 |
| **Protocol version date:** | June 10, 2019 |
| **Sponsor:** | Hangzhou Zhongmeihuadong Pharmaceutical Co., Ltd. |
| **Statistical analysis:** | Peking University Clinical Research Institute |
| **Version No.:** | V 1.0 |
| **Version date:** | July 25, 2023 |

**Confidentiality Statement**

This document is intended for use only by the investigators involved in this study, their medical institutions, ethics committees or institutional review committees, and other authorized staff. May not be used, divulged, published or otherwise disclosed without the written permission of the sponsor.

# Signature Page

| | |
|---|---|
| Study title | Study of Tacrolimus vs Mycophenolate Mofetil in Pediatric Patients with Frequently Relapsing or Steroid-dependent Nephrotic Syndrome: a Prospective, Randomized, Multicenter, Open-label, and Parallel-arm Study on Efficacy and Safety |

Statistical analysis issued by: Peking University Clinical Research Institute

I have read this plan and confirm that it accurately describes the implementation plan for the statistical analysis of the study.

SAP prepared by (signature): ______________________

Date: ______________________

Sponsor: Hangzhou Zhongmeihuadong Pharmaceutical Co., Ltd.

Sponsor (signature): ______________________

Date: ______________________

# Revision History

| Version No. | Amended on | Revised by |
|---|---|---|
| V 0.3 | December 5, 2019 | |
| V 0.4 | March 24, 2023 | |
| V 0.5 | May 19, 2023 | |
| V 0.6 | July 3, 2023 | |
| V 0.7 | July 21, 2023 | |
| V 0.8 | July 24, 2023 | |
| V 1.0 | July 25, 2023 | |

# CONTENTS

**1. Abbreviations......10**

**2. Study title......12**

**3. Study objectives and endpoints......12**

3.1 To evaluate the efficacy of the test drugs on the frequently relapsing or steroid-dependent nephrotic syndrome in pediatric patients......12

3.2 To assess the safety outcomes of test drugs......13

**4. Study design......13**

4.1. Design overview......13

4.2. Design principle and case allocation......13

*4.2.1 Difference analysis......13*

*4.2.2 Determination of the number of cases......14*

4.3. Research plan......15

4.4. Clinical trial flow chart......16

4.5. Selection of cases......24

*4.5.1. Inclusion criteria......24*

*4.5.2. Exclusion criteria......24*

*4.5.3. Elimination criteria......25*

*4.5.4. Withdrawal criteria......25*

*4.5.5. Drop-out and treament......26*

4.5.5.1 Definition of drop-out......26

4.5.5.2 Treatment of drop-out cases......26

*4.5.6. Relevant definitions in inclusion and exclusion criteria......27*

**5. Test drugs and their allocation......27**

5.1. Drug name and specifications......27

5.2. Administration method, dose, route and course of treatment......28

*5.2.1. Initial administration protocol......28*

*5.2.2. Treatment of relapses......28*

*5.2.3. Dose adjustment of tacrolimus (FK506)......29*

*5.2.4. Dose adjustment of mycophenolate mofetil (MMF)......29*

5.3. concomitant medication......30

5.4. MEDICATION ADHERENCE......30

**6. Evaluation of efficacy and safety......31**

6.1. Primary efficacy outcomes......31

6.2. Efficacy evaluation......31

*6.2.1. Primary observation outcomes: 1-year relapse-free survival......31*

*6.2.2. Secondary observation outcomes:......31*

*6.2.3. Efficacy criteria* ........ *31*
*6.2.4. Relapse criteria* ........ *31*
6.3. Safety evaluation ........ 32
**7. Statistical analysis ........ 32**
7.1. Analysis data set ........ 32
7.2. General considerations of date analyses ........ 32
*7.2.1. General Statistical methods* ........ *32*
*7.2.2. Statistical inference method* ........ *33*
**8. Data handling conventions ........ 33**
8.1. Content of Statistical analysis ........ 34
*8.1.1. Basic value equilibrium analysis* ........ *34*
*8.1.2. Effectiveness analysis* ........ *34*
*8.1.3. Primary efficacy analysis* ........ *34*
*8.1.4. Secondary efficacy analysis* ........ *35*
*8.1.5. Other analysis* ........ *35*
*8.1.6. Safety analysis* ........ *35*
**9. Statistical analysis results ........ 37**
9.1. Research overview ........ 37
Table 1. Distribution of subjects at each site (All enrolled subjects) ........ 37
Table 2. Distribution of subjects at each site ........ 38
Table 3. Trial completion and distribution of analysis data set (All enrolled subjects) ........ 38
9.2. Demographic characteristics (FAS) ........ 39
Table 4. Baseline demographics ........ 39
Table 5. Baseline vital signs ........ 40
Table 6. Family history and personal history ........ 42
Table 7. History of other diseases, concomitant medications and nephrotic syndrome ........ 45
Table 8. Visual palpation of physical examination Items ........ 46
Table 9. Diagnostic consultation of physical examination Items ........ 48
9.3. Baseline examinations (FAS) ........ 50
Table 10. DNA/antibody tests ........ 50
Table 11. Hepatitis virus ........ 51
Table 12. Examination of mycobacterium tuberculosis and fungi ........ 52
Table 13. Urine test ........ 52
Table 14. Glomerular filtration rate ........ 53
Table 15. Retained biological samples of blood and urine ........ 53
Table 16. Chest X-ray ........ 54
Table 17. ECG examinations ........ 54
Table 18. Primary diagnosis ........ 54
9.4. Efficacy analysis (FAS/PPS) ........ 55
*9.4.1. Primary efficacy indicators: 1-year relapse-free survival rate* ........ *55*

Table 19. 1-year relapse-free survival analysis.......55
Figure 1. 1-year relapse-free survival analysis chart (FAS).......56
Figure 2. 1-year relapse-free survival analysis chart (PPS).......56
*9.4.2. Secondary efficacy indicators.......57*
9.4.2.1. The first relapse survival analysis after enrollment.......57
Table 20. The time of the first relapse after enrollment.......57
Table 21. The first relapse survival analysis after enrollment.......57
9.4.2.2. 6-month relapse-free survival analysis after enrollment.......58
Table 22. 6-month relapse-free survival analysis after enrollment.......58
Figure 3. 6-month relapse-free survival analysis chart (FAS).......58
Figure 4. 6-month relapse-free survival analysis chart (PPS).......58
9.4.2.3. Serum renal function.......59
Table 23. Changes in eGFR relative to baseline at each visit.......59
Figure 5. Quantitative variables of eGFR at each visit.......63
Table 24. Change in Scr from baseline at each visit.......63
Figure 6. Quantitative variables of Scr at each visit.......63
Table 25. Change in serum uric acid from baseline at each visit.......63
Figure 7. Quantitative variables of serum uric acid at each visit.......64
Table 26. Change in blood urea nitrogen from baseline at each visit.......64
Figure 8. Quantitative variables of blood urea nitrogen at each visit.......64
Table 27. Change in serum cystatin C from baseline at each visit.......64
Figure 9. Quantitative variables of serum cystatin C at each visit.......64
9.4.2.4. Cumulative steroid dosage.......64
Table 28. Total amount of steroids taken during the study.......64
Table 29. Total amount of steroids taken during 6 months after enrollment.......64
9.4.2.5. Vital signs.......65
Table 30. Change in systolic blood pressure from baseline at each visit.......65
Table 31. Change in diastolic blood pressure from baseline at each visit.......65
Table 32. Change in height from baseline at each visit.......65
Table 33. Change in weight from baseline at each visit.......65
Table 34. Height percentile.......65
9.4.2.6. Blood lipid.......66
Table 35. Change in triglyceride from baseline at each visit.......66
Table 36. Change in total cholesterol from baseline at each visit.......66
9.4.2.7. Haemoglobin and albumin (ALB).......66
Table 37. Change in haemoglobin from baseline at each visit.......66
Table 38. Change in ALB from baseline at each visit.......66
9.4.2.8. Urine test.......66
Table 39. Change in 24hr urine protein (>3 years old) from baseline at each visit.......66
Table 40. Change in urine protein/creatinine (morning urine) from baseline at each visit.......66
9.4.2.9. Serum liver function and fasting blood glucose.......66
Table 41. Change in glutamyl transpeptidase (GT) from baseline at each visit.......66

Table 42. Change in alanine aminotransferase (ALT) from baseline at each visit ........ 66
Table 43. Change in aspartate aminotransferase (AST) from baseline at each visit ........ 66
Table 44. Change in lactate dehydrogenase from baseline at each visit ........ 66
Table 45. Change in total bilirubin from baseline at each visit........ 66
Table 46. Change in direct bilirubin from baseline at each visit........ 66
Table 47. Change in alkaline phosphatase from baseline at each visit ........ 66
Table 48. Change in total protein from baseline at each visit ........ 66
Table 49. Change in globulin from baseline at each visit ........ 67
Table 50. Change in fasting blood glucosefrom baseline at each visit........ 67
9.4.2.10. Blood routine examination ........ 67
Table 51. Change in leukocyte count (WBC) from baseline at each visit........ 67
Table 52. Change in erythrocyte count (RBC) from baseline at each visit ........ 67
Table 53. Change in platelet count (PLT) from baseline at each visit........ 67
Table 54. Change in lymphocyte percentage (LY) from baseline at each visit ........ 67
9.4.2.11. Urine routine examination........ 67
Table 55. Change in leukocyte from baseline at each visit........ 67
Table 56. Change in erythrocyte from baseline at each visit ........ 67
Table 57. Change in pH from baseline at each visit ........ 67
Table 58. Change in urine protein from baseline at each visit ........ 67
9.4.2.12. Plasma concentration ........ 67
Table 59. Change in tacrolimus concentration in plasma from baseline at each visit ........ 67
Table 60. Change in mycophenolic acid concentration in plasma from baseline at each visit........ 67
*9.4.3. Disease remission* ........ *67*
Table 61. Disease remission at each visit ........ 67
9.5. Safety analysis (SS) ........ 68
*9.5.1. Adverse event (AE)* ........ *68*
Table 62. Between-group comparison of AE........ 68
Table 63. characteristics of AE in each group ........ 68
Table 64. Between-group comparison of SAE ........ 69
Table 65. Summary of AE base on SOC and PT coding........ 69
Table 66. Summary of adverse reactions base on SOC and PT coding ........ 70
*9.5.2. Vital signs* ........ *71*
Table 67. Change in heart rate from baseline at each visit ........ 71
Table 68. Change in respiratory rate from baseline at each visit ........ 75
*9.5.3. Blood routine examination* ........ *75*
Table 69. Summary of blood routine examination-Leukocyte count (WBC) ........ 75
Table 70. Summary of blood routine examination-Lymphocyte percentage (LY) ........ 76
Table 71. Shift-table of blood routine examination results before and after treatment-Leukocyte count (WBC)........ 76
Table 72. Shift-table of blood routine examination results before and after treatment-Erythrocyte count (RBC)........ 77
Table 73. Shift-table of blood routine examination results before and after treatment-Haemoglobin (Hb)........ 77
Table 74. Shift-table of blood routine examination results before and after treatment-Platelet count (PLT)........ 77
Table 75. Shift-table of blood routine examination results before and after treatment-Lymphocyte percentage (LY) ........ 77

*9.5.4. Urine routine examination* ........ 77
Table 76. Shift-table of urine routine examination results before and after treatment-Leukocyte ........ 77
Table 77. Shift-table of urine routine examination results before and after treatment-Erythrocyte ........ 77
Table 78. Shift-table of urine routine examination results before and after treatment-pH ........ 77
Table 79. Shift-table of urine routine examination results before and after treatment-Urine protein ........ 77
Table 80. Shift-table of urine routine examination results before and after treatment-Color ........ 77
Table 81. Shift-table of urine routine examination results before and after treatment-Definition ........ 77
*9.5.5. Serum biochemistry* ........ 78
Table 82. Shift-table of serum biochemistry results before and after treatment-Glutamyl transpeptidase (GT) ........ 78
Table 83. Shift-table of serum biochemistry results before and after treatment-Alanine aminotransferase (ALT) ........ 78
Table 84. Shift-table of serum biochemistry results before and after treatment-Aspartate aminotransferase (AST) ........ 78
Table 85. Shift-table of serum biochemistry results before and after treatment-Lactate dehydrogenase ........ 78
Table 86. Shift-table of serum biochemistry results before and after treatment-Total bilirubin ........ 78
Table 87. Shift-table of serum biochemistry results before and after treatment-Direct bilirubin ........ 78
Table 88. Shift-table of serum biochemistry results before and after treatment-Alkaline phosphatase ........ 78
Table 89. Shift-table of serum biochemistry results before and after treatment-Scr ........ 78
Table 90. Shift-table of serum biochemistry results before and after treatment-Uric acid ........ 78
Table 91. Shift-table of serum biochemistry results before and after treatment-Blood urea nitrogen ........ 78
Table 92. Shift-table of serum biochemistry results before and after treatment-Cystatin C ........ 78
Table 93. Shift-table of serum biochemistry results before and after treatment-Fasting blood glucose ........ 78
Table 94. Shift-table of serum biochemistry results before and after treatment-Total cholesterol ........ 78
Table 95. Shift-table of serum biochemistry results before and after treatment-Triglyceride ........ 78
Table 96. Shift-table of serum biochemistry results before and after treatment-Total protein ........ 78
Table 97. Shift-table of serum biochemistry results before and after treatment-Albumin ........ 78
Table 98. Shift-table of serum biochemistry results before and after treatment-Globulin ........ 78
*9.5.6. DNA/antibody test* ........ 78
Table 99. Shift-table of DNA/antibody test results before and after treatment-Blood EBV-DNA ........ 78
Table 100. Shift-table of DNA/antibody test results before and after treatment-IgM ........ 78
Table 101. Shift-table of DNA/antibody test results before and after treatment-IgG ........ 78
*9.5.7. Urine test* ........ 78
Table 102. Shift-table of urine test results before and after treatment-24hr urine protein (>3 years old) ........ 78
Table 103. Shift-table of urine test results before and after treatment-Urine protein/creatinine ........ 78
*9.5.8. Glomerular filtration rate* ........ 79
Table 104. Shift-table of eGFR results before and after treatment-eGFR ........ 79
*9.5.9. Chest X-ray* ........ 79
Table 105. Shift-table of chest X-ray results before and after treatment ........ 79
*9.5.10. ECG* ........ 79
Table 106. Shift-table of ECG examination results before and after treatment ........ 79
*9.5.11. Concomitant medication and medication adherence* ........ 79
Table 107. Concomitant medication ........ 79
Table 108. Medication adherence ........ 80
**10. Attachments** ........ 81

Table 109. List of subjects drop-out (All randomized subjects) ........ 81
Table 110. List of subjects exclusions (All randomized subjects) ........ 81
Table 111. List of protocol deviations (All randomize subjects) ........ 81
Table 112. List of demographic characteristics (FAS) ........ 81
Table 113. List of family history and personal history (FAS) ........ 81
Table 114. List of history of other diseases and concomitant medications (FAS) ........ 82
Table 115. List of history of nephrotic syndrome (FAS) ........ 82
Table 116. List of treatment history for nephrotic syndrome (FAS) ........ 82
Table 117. List of disease relapse (urine protein) situations (FAS) ........ 82
Table 118. List of primary efficacy indicator (1-year relapse-free) situations (FAS/PPS) ........ 82
Table 119. List of secondary efficacy indicator (first relapse after enrollment) situations (FAS/PPS) ........ 83
Table 120. List of secondary efficacy indicator (6-month relapse-free) situations (FAS/PPS) ........ 83
Table 121. List of blood routine examination results that were normal before treatment and abnormal after treatment (SS) ........ 83
Table 122. List of urine routine examination results that were normal before treatment and abnormal after treatment (SS) 83
Table 123. List of serum biochemistry results that were normal before treatment and abnormal after treatment (SS) ........ 83
Table 124. List of urine test results that were normal before treatment and abnormal after treatment (SS) ........ 84
Table 125. List of eGFR results that were normal before treatment and abnormal after treatment (SS) ........ 84
Table 126. List of chest X-ray results that were normal before treatment and abnormal after treatment (SS) ........ 84
Table 127. List of ECG examination results that were normal before treatment and abnormal after treatment (SS) ........ 84
Table 128. List of AE (SS) ........ 85
Table 129. List of AE-continued (SS) ........ 85
Table 130. List of SAE (SS) ........ 85
Table 131. List of SAE-continued (SS) ........ 85
Table 132. List of concomitant medication (SS) ........ 86

# 1. Abbreviations

| Abbreviations | Description |
|---|---|
| ALT | Alanine aminotransferase |
| ALB | Albumin |
| AUC | Area under curve |
| ACEi | Angiotensin converting enzyme inhibitor |
| ARB | Angiotensin II receptor blocker |
| AE | Adverse event |
| ANCOVA | Analysis of covariance |
| BID | Bis in die |
| CMV | Cytomegalovirus |
| CsA | Cyclosporin A |
| CR | Complete remission |
| CSR | Clinical Study Report |
| CRF | Case Report Form |
| CMH | Cochran’s and Mantel-Haenszel |
| DAE | Drug adverse event |
| EB | Epstein-Barr |
| EBV-DNA | Epstein-Barr virus deoxyribonucleic acid |
| eGFR | Glomerular filtration rate |
| eCRF | Electronic Case Report Form |
| FAS | Full analysis set |
| FR | Frequent relapse |
| FDA | Food and Drug Administration |
| FK506 | Tacrolimus |
| FK-BP-12 | Tacrolimus binding protein-12 |
| FK-BP-52 | Tacrolimus binding protein-52 |

| | |
|---|---|
| FK506-FKBP | Tacrolimus - tacrolimus binding protein |
| FRNS | Frequently relapsing nephrotic syndrome |
| GCP | Good Clinical Practice |
| g | Gram |
| GTP | Guanine nucleotide |
| HBV | Hepatitis B virus |
| HbsAg | Hepatitis B surface antigen |
| HbeAg | Hepatitis B E antigen |
| HbcAb | Hepatitis B core antigen |
| HCV | Hepatitis C virus |
| hr | Hour |
| ITT | Intentionality |
| LSMEAN | Least squares mean |
| LC-MS | Liquid chromatography-mass spectrometry |
| MMF | Mycophenolate mofetil |
| MPA | Mycophenolic acid |
| MPA-d3 | Mycophenolic acid-d3 |
| MPA-AUC | Mycophenolic acid - Area under curve |
| MI | Myocardial infarction |
| mg | Milligram |
| NR | No remission |
| PP | Per-protocol |
| PR | Partial remission |
| PML | Progressive multifocal leukoencephalopathy |
| PRCA | Pure red cell aplasia |
| PPS | Per-protocol set |
| SAS 9.4 | STATISTICAL ANALYSIS SYSTEM Version 9.4 |
| SD | Steroid-dependent |

| | |
|---|---|
| SDNS | Steroid-dependent nephrotic syndrome |
| SRNS | Steroid-resistant nephrotic syndrome |
| SCV | Site close-out visit |
| SAE | Serious adverse event |
| SOP | Standard Operation Procedures |
| SS | Safety set |
| Scr | Serum creatinine |
| TBiL | Total bilirubin |
| TIMP-1 | Tissue inhibitor of metalloproteinase-1 |
| TIA | Transient ischemic attack |
| WHO | World Health Organization |
| WBDC | Web-based data collection |
| α | Inspection level |
| β | Power of test |
| δ | Cut-off value |

## 2. Study title

Study of Tacrolimus vs Mycophenolate Mofetil in Pediatric Patients with Frequently Relapsing or Steroid-dependent Nephrotic Syndrome: a Prospective, Randomized, Multicenter, Open-label, and Parallel-arm Study on Efficacy and Safety

## 3. Study objectives and endpoints

### 3.1 To evaluate the efficacy of the test drugs on the frequently relapsing or steroid-dependent nephrotic syndrome in pediatric patients

**Efficacy outcomes:**

**Primary observation outcomes: 1-year relapse-free survival**

**Secondary observation outcomes:** Renal function; cumulative steroid dosage; blood pressure; height;

weight; blood lipids; hemoglobin; serum albumin; urinary protein/creatinine (morning urine); 24hr urinary protein quantification (applicable to subjects > 3 years old); frequency of relapses; the time of the first relapse after enrollment; relapse-free survival in the first six months after enrollment; adverse reactions;

### 3.2 To assess the safety outcomes of test drugs

Changes of vital signs (respiration and heart rate), routine blood and urine tests, liver function, renal function, blood glucose, ECG, chest X-ray, and observation of clinically reported adverse events and adverse reactions.

## 4. Study design

### 4.1. Design overview

This trial is a prospective, randomized, multicenter, open-label, parallel-arm clinical study, using the Logrank method to compare the difference of 1-year relapse free survival between the two groups. Enrollment was completed by 12 sites using competitive enrollment. The whole observation period is 1 year for this trial. Eligible patients who sign the informed consent form will be randomly divided into FK506 Group or MMF Group at the ratio of 1:1. Both groups follow the "clinical trial flow chart" in operation.

### 4.2. Design principle and case allocation

#### 4.2.1 Difference analysis

In this research, Logrank method is used to compare the difference of 1-year relapse-free survival between the two groups.

Eligible patients will be randomly divided into FK506 Group or MMF Group at the ratio of 1:1. The random codes are generated by the computer and the random envelopes are made according to these random codes. Grouping information is attached in each envelope. Each code is provided with a corresponding random envelope, and the random numbers are given to the subjects from small to large according to the time sequence when the subjects meet the inclusion criteria. The corresponding

random envelopes can only be opened when grouping. Any random number falling off midway after use cannot be used again.

### 4.2.2 Determination of the number of cases

It is estimated that 1-year relapse-free survival is 0.56 in MMF Group, and 0.70 in FK506 Group. The enrollment time of this research is expected to be 2 years, and the total time of this research is 3 years, with a test level $\alpha = 0.05$, and a power of test $1-\beta = 80\%$. It is estimated to have 114 patients in MMF group and 115 patients in FK506 group, with a total of 229 patients. With the consideration of 15% loss of follow-up, a total of 270 patients are needed, including 135 patients in each group.

## 4.3. Research plan

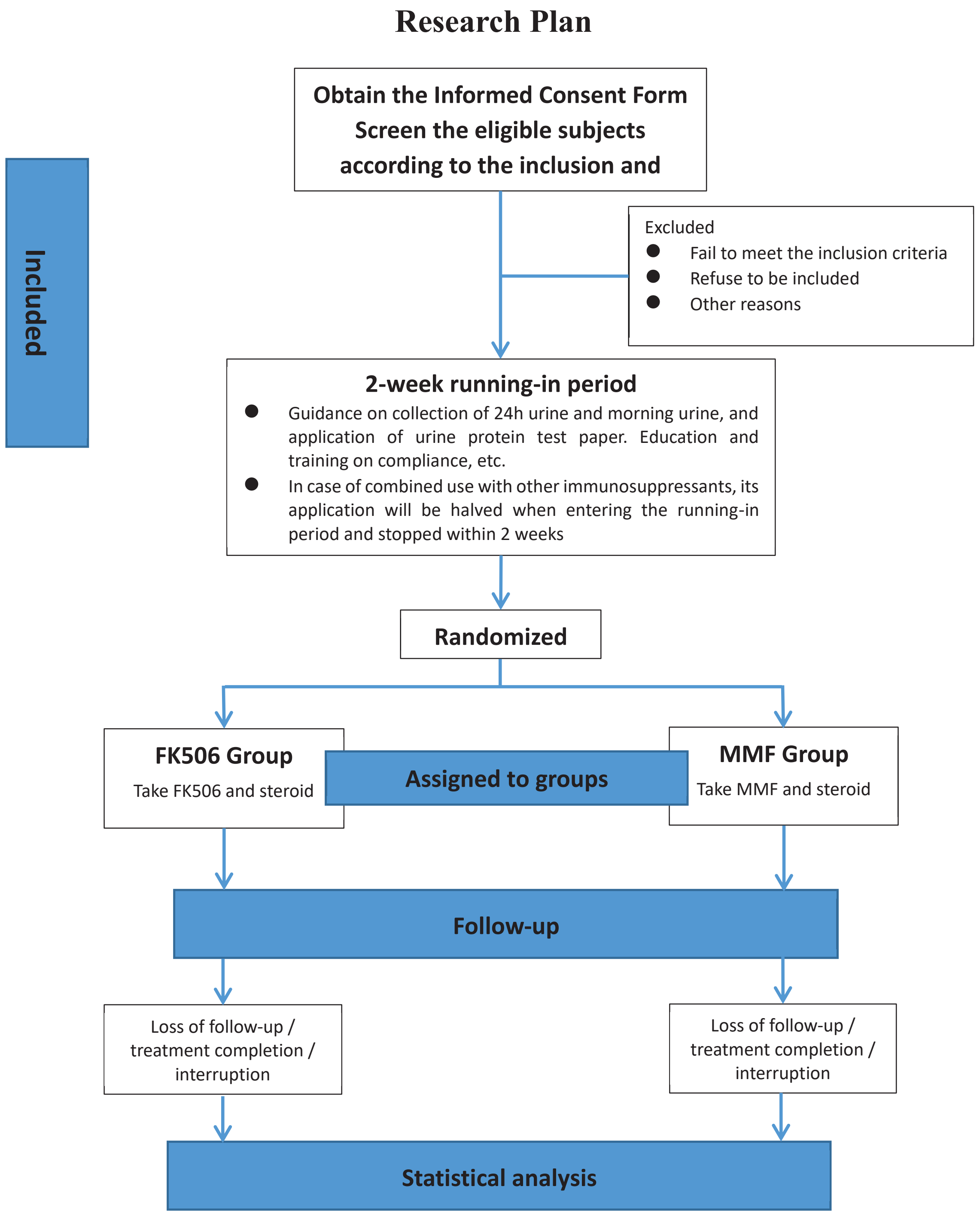

### 4.4. Clinical trial flow chart

## Clinical trial flow chart

| Items | Screening and enrollment period | | Treatment and follow-up period | | | | | | | |
|---|---|---|---|---|---|---|---|---|---|---|
| | Screen (V0) | Visit 1 (V1) | Visit 2 (V2) | Visit 3 (V3) | Visit 4 (V4) | Visit 5 (V5) | Visit 6 (V6) | Visit 7 (V7) | Visit 8 (V8) | Visit 9 (V9) |
| Follow-up week | Day -30 - 0 | Included | Day 7 | Day 28 | Day 56 | Day 112 | Day 168 | Day 224 | Day 280 | Day 364 |
| Sign the informed consent gorm | √ | | | | | | | | | |
| Inclusion/exclusion criteria | | √ | | | | | | | | |

| Items | Screening and enrollment period | | Treatment and follow-up period | | | | | | | |
|---|---|---|---|---|---|---|---|---|---|---|
| | Screen (V0) | Visit 1 (V1) | Visit 2 (V2) | Visit 3 (V3) | Visit 4 (V4) | Visit 5 (V5) | Visit 6 (V6) | Visit 7 (V7) | Visit 8 (V8) | Visit 9 (V9) |
| Stop the original immunosuppressants after 2 weeks | $ | $ | | | | | | | | |
| Randomized | | √ | | | | | | | | |
| General data | √ | | | | | | | | | |
| Medical history (course of disease, frequency of relapses in 1 year and 6 months before enrollment, steroid dosage) | √ | | | | | | | | | |

| Items | Screening and enrollment period | | Treatment and follow-up period | | | | | | | |
|---|---|---|---|---|---|---|---|---|---|---|
| | Screen (V0) | Visit 1 (V1) | Visit 2 (V2) | Visit 3 (V3) | Visit 4 (V4) | Visit 5 (V5) | Visit 6 (V6) | Visit 7 (V7) | Visit 8 (V8) | Visit 9 (V9) |
| Vital signs | √ | √ | √ | √ | √ | √ | √ | √ | √ | √ |
| Height and weight | √ | √ | √ | √ | √ | √ | √ | √ | √ | √ |
| Physical examination | √ | ◎ | √ | √ | √ | √ | √ | √ | √ | √ |
| Blood routine examination | √ | ◎ | √ | √ | √ | √ | √ | √ | √ | √ |
| Urine routine examination | √ | ◎ | √ | √ | √ | √ | √ | √ | √ | √ |
| Fasting blood glucose | √ | | √ | √ | √ | √ | √ | √ | √ | √ |
| Serum liver function | √ | ◎ | √ | √ | √ | √ | √ | √ | √ | √ |
| Blood lipid | √ | ◎ | √ | √ | √ | √ | √ | √ | √ | √ |

| Items | Screening and enrollment period | | Treatment and follow-up period | | | | | | | |
|---|---|---|---|---|---|---|---|---|---|---|
| | Screen (V0) | Visit 1 (V1) | Visit 2 (V2) | Visit 3 (V3) | Visit 4 (V4) | Visit 5 (V5) | Visit 6 (V6) | Visit 7 (V7) | Visit 8 (V8) | Visit 9 (V9) |
| Serum renal function | √ | ◎ | √ | √ | √ | √ | √ | √ | √ | √ |
| Blood EBV-DNA, and CMV antibody | √ | | | | | | | | | √ |
| 24hr urinary protein (>3 years old) | √ | ◎ | √ | √ | √ | √ | √ | √ | √ | √ |
| Urinary protein / creatinine | √ | ◎ | √ | √ | √ | √ | √ | √ | √ | √ |
| eGFR | √ | ◎ | √ | √ | √ | √ | √ | √ | √ | √ |
| Tacrolimus concentration in plasma | | | √ | √ | √ | √ | √ | √ | | √ |

| Items | Screening and enrollment period | | Treatment and follow-up period | | | | | | | |
|---|---|---|---|---|---|---|---|---|---|---|
| | Screen (V0) | Visit 1 (V1) | Visit 2 (V2) | Visit 3 (V3) | Visit 4 (V4) | Visit 5 (V5) | Visit 6 (V6) | Visit 7 (V7) | Visit 8 (V8) | Visit 9 (V9) |
| Mycophenolic acid concentration in plasma | | | √ | | | | √ | | | |
| Chest X-ray | √ | | | | | | | | | √ |
| ECG | √ | | | | | | | | | √ |
| Markers of hepatitis B and hepatitis C virus | √ | | | | | | | | | |
| Examination of Mycobacterium tuberculosis and fungi | √ | | | | | | | | | |

| **Items** | **Screening and enrollment period** | | **Treatment and follow-up period** | | | | | | | |
|---|---|---|---|---|---|---|---|---|---|---|
| | Screen (V0) | Visit 1 (V1) | Visit 2 (V2) | Visit 3 (V3) | Visit 4 (V4) | Visit 5 (V5) | Visit 6 (V6) | Visit 7 (V7) | Visit 8 (V8) | Visit 9 (V9) |
| Biological samples of blood and urine | √ | | | | | | √ | | | √ |
| Drug distribution and measurement | | √ | √ | √ | √ | √ | √ | √ | √ | √ |
| Guidance on the administration of test drug | | √ | √ | √ | √ | √ | √ | √ | √ | √ |
| Clinical efficacy (relapse or no) | | | √ | √ | √ | √ | √ | √ | √ | √ |
| Concomitant medication | √ | √ | √ | √ | √ | √ | √ | √ | √ | √ |

| Items | Screening and enrollment period | | Treatment and follow-up period | | | | | | | |
|---|---|---|---|---|---|---|---|---|---|---|
| | Screen (V0) | Visit 1 (V1) | Visit 2 (V2) | Visit 3 (V3) | Visit 4 (V4) | Visit 5 (V5) | Visit 6 (V6) | Visit 7 (V7) | Visit 8 (V8) | Visit 9 (V9) |
| Adverse event | | | √ | √ | √ | √ | √ | √ | √ | √ |
| Record case report form | √ | √ | √ | √ | √ | √ | √ | √ | √ | √ |

Remarks:

1. Liver function: ALT, TBiL, ALB; Renal function: creatinine, blood Blood urea nitrogen, uric acid, Cystatin C;
2. Blood lipids: Triglycerides, cholesterol; Urinary protein / creatinine: morning urine; Markers of hepatitis B and hepatitis C virus: HBsAg, HBeAg, HBcAb, HCV;
3. Mycobacterium tuberculosis examination: T-sport; Fungal examination: fungal G test or other; Estimated glomerular filtration rate: eGFR = K × height (cm) / serum creatinine (mg/dL) (see Appendix);
4. Visit time window: ±3 days for visit 2, and ±7 days for other visits;

5. $ Items are applicable to the pediatric patients with combined use of other immunosuppressants before enrollment; ◎ Items are optional, depending on the judgment and decision of the investigators;

6. This research accepts the examination data from our hospital within 2 weeks before the screening period, and no repeated test is needed.

### 4.5. Selection of cases

#### 4.5.1. Inclusion criteria

Only those who meet all the conditions listed in this column can be considered for inclusion in this research:

(1) Steroid-sensitive nephrotic syndrome with frequent relapse (FRNS) or steroid dependence (SDNS).

(2) Age: 2-18 years old.

(3) Normal renal function: eGFR ≥90 mL/min/1.73m$^2$.

(4) At the time of enrollment, urinary protein <1+ or urinary protein/creatinine <0.2g/g (<20 mg/mmol) in the morning for 3 consecutive days or more.

(5) No use of tacrolimus, mycophenolate mofetil, cyclosporine A, rituximab or cyclophosphamide within 2 years before enrollment.

#### 4.5.2. Exclusion criteria

Anyone with one of the following conditions cannot be included in this research:

(1) Steroid-resistant nephrotic syndrome (SRNS).

(2) Family history of nephrotic syndrome, chronic glomerulonephritis or uremia.

(3) Leukopenia (leukocyte ≤3.0*10$^9$/L).

(4) Moderate and severe anemia (hemoglobin <9.0 g/dL).

(5) Thrombocytopenia (platelet count <100*10$^{12}$/L).

(6) Those with positive HBV serological indexes (HBsAg or/and HBeAg or/and HBcAb), positive HCV or abnormal liver function (ALT or total bilirubin exceeding 2 times or more of the upper limit, with a continuous increase for 2 weeks).

(7) There are chronic active infections such as Epstein-Barrvirus, cytomegalovirus or Mycobacterium tuberculosis, and the usage of steroids and immunosuppressive agents may aggravate the state of an illness.

(8) Secondary nephrotic syndrome (such as purpura nephritis ,lupus nephritis,etc).

(9) Those who with hematological or endocrine system diseases as well as serious organs illness such as heart, liver or kidney.

(10) Other autoimmune diseases or primary immunodeficiency or tumors.

(11) Those who was known be sensitized to tacrolimus, mycophenolate mofetil, glucocorticoids or any ingredient of the above drugs.

(12) Those who have participated in other clinical trials within three months before enrollment.

(13) Those who was not suitable for participating this study judged by investigator.

### 4.5.3. Elimination criteria

- Those who fail to take the test drugs as required, with a compliance <80% or >120%.
- Those who cannot cooperate well or complete the follow-up as planned, and those who change the drug dose at will.

### 4.5.4. Withdrawal criteria

Patients are free to withdraw from the trial at any time without any reason.

Patients must withdraw from this research in the following conditions:

(1) Withdrawal of the Informed Consent Form.

(2) Violation of the inclusion criteria or compliance with the exclusion criteria.

(3) No administration of the test drug according to the Protocol for 14 consecutive days due to various reasons.

(4) For two consecutive times, the measured value of SCr with an interval of at least 4 weeks has increased more than twice the baseline, and eGFR < 60mL/min.1.73m$^2$.

(5) The target concentration of tacrolimus or mycophenolate mofetil can not be reached after dose adjustment for one month.

(6) ALT or total bilirubin levels reach more than 2 times of the upper limit after medication, with a continuous increase for 2 weeks; if ALT or total bilirubin levels still rise to more than 2 times of the

upper limit after 2 weeks of liver protection drug treatment, stop the test drug. In case of no recovery after 2 weeks of drug withdrawal, the patient shall withdraw from the test.

(7) Occurrence of unknown adverse events of unacceptable nature, severity and/or duration, or occurrence of known unacceptable adverse events or the occurrence frequency exceeding expectations.

(8) The clinical events or safety-affecting events result in withdrawal from the clinical trial, and the investigators believe that such subjects shall withdraw from the trial.

(9) If pregnancy occurs during treatment, withdraw from the trial.

(10) Unauthorized use of drugs prohibited by this research.

### 4.5.5. Drop-out and treament

#### 4.5.5.1 Definition of drop-out

All patients who have completed the Informed Consent Form and screened to be enrolled in the trial have the right to withdraw at any time. Whenever and for whatever reason, subjects who fail to complete the observation period specified in the Protocol are considered as drop-out cases, including the following situations:

- The subject has serious adverse events and the clinician believes that he/she should withdraw from the clinical trial;
- The subject shows aggravated condition or has other symptoms affecting the trial observation during the trial, and the clinician believes that he/she should withdraw from the clinical trial;
- Important deviations occur in the implementation of clinical trial protocol, such as poor compliance, which would cause difficulty in evaluating the drug effect;
- The subject is unwilling to continue the clinical trial and asks to withdraw from the clinical trial, or loss of follow-up has occurred although no request to withdraw from the trial is proposed.

#### 4.5.5.2 Treatment of drop-out cases

The investigators shall contact the drop-off subjects by means of visiting, making an appointment for follow-up, calling, letter and so on, to ask the reasons, record the last medication time and complete the evaluation Items that can be completed. For the cases withdrawing from the trial due to allergy or

other adverse reactions and ineffective treatment, the investigators shall take corresponding treatment measures according to the actual situation of the subjects. The investigators shall properly keep the relevant test data of the drop-off cases, not only for filing, but also for full analysis and statistics.

### 4.5.6. Relevant definitions in inclusion and exclusion criteria

**Steroid-sensitive nephrotic syndrome (SSNS):** Complete remission after 4 weeks of prednisone or prednisolone at standard dose [2mg/kg.d or 60mg/m$^2$. d].

**Steroid-resistant nephrotic syndrome (SRNS):** Lack of complete remission at 4 weeks of therapy with daily prednisone or prednisolone at standard dose [2mg/kg.d or 60mg/m$^2$. d].

**Steroid-dependent nephrotic syndrome (SDNS):** Two consecutive relapses during therapy with prednisone or prednisolone (either at full dose or during tapering) or within 15 days of prednisone or prednisolone discontinuation

**Frequently relapsing nephrotic syndrome (FRNS):** ≥2 relapses per 6 months within 6months of disease onset or 24 relapses per 12 months in any subsequent 12-month period.

**Secondary nephrotic syndrome:** It refers to the nephrotic syndrome with clear causes. Secondary nephrotic syndrome can be caused by autoimmune diseases (such as systemic lupus erythematosus), diabetes, infections (such as bacteria and hepatitis B virus), circulatory system diseases, drug poisoning, etc.

## 5. Test drugs and their allocation

### 5.1. Drug name and specifications

(1) Tacrolimus capsules (trade name: Saifukai®， capsules, 1.0 mg/capsule, 0.5 mg/capsule); Storage conditions: Keep away from sunlight, and store in a dry place below 25°C after sealing.

(2) Mycophenolate mofetil dispersible tablets (trade name: Saikeping®, tablets, 250 mg/tablet); Storage conditions: Store under 30°C and keep away from sunlight.

(3) Storage conditions of prednisone tablets (tablets, 5 mg/tablet): Keep away from sunlight, and store at room temperature after sealing.

[Prednisone tablets can be replaced by other glucocorticoids during the test, but the dose shall have

the same therapeutic effect, for example prednisone 5 mg = prednisolone 5 mg, prednisone 5 mg = methylprednisolone 4mg, etc.]

## 5.2. Administration method, dose, route and course of treatment

### 5.2.1. Initial administration protocol

**FK506 dose:** 0.05-0.10mg/kg/day， BID

Maintained concentration: In the induction period (the first six months after treatment) (visit 2, 3, 4, 5 and 6), the trough plasma concentration of tacrolimus is 5 - 10 ng/mL;

**In the maintenance period, drug dose is gradually reduced when the patient's condition is stable: In the last six months after administration (visit 7, 8, and 9), the** trough concentration **is <5 ng/mL**

**MMF dose:** 20-30 mg/kg/day, BID

Plasma concentration (AUC) [Formula: MPA-AUC =7.75+(6.49*C0)+(0.76*C0.5)+(2.43*C2)]

Maintained concentration: In the induction period (the first six months after treatment) (visit 2, 3, 4, 5 and 6)

**MPA-AUC=30 - 50 μg.h/mL**

**In the maintenance period, drug dose is gradually reduced when the patient's condition is stable: In the last six months after administration (visit 7, 8, and 9), MPA-AUC≤40 μg.h/mL**

**Prednisone dose:** The initial dose is 1.0-1.5 mg/kg every other day or 0.5-0.75 mg/kg every day. The dose shall be reduced after 4 weeks by 0.25 mg/kg (qd alt) or 0.125 mg/kg (every day) every 2-4 weeks. If the condition is stable, continue to reduce to 5mg (body surface area > 1 $m^2$) and 2.5mg (body surface area < 1 $m^2$) qd alt. Maintain at such doses until this research is completed.

### 5.2.2. Treatment of relapses

If the disease is complicated with infection in the relapse for the pediatric patients, anti-infection treatment can be given first. After infection control, if the disease still shows relapse, the prednisone dose shall be restored to 1.0-1.5 mg/kg per day, with a maximum dose ≤60mg; If there is no infection at the time of relapse in the pediatric patients, the prednisone dose should be restored to 1.0-1.5 mg/kg per day directly, with a maximum dose ≤60mg. 5-7 days after the urine protein turns into negative results, the prednisone dose is reduced by 1.0-1.5 mg/kg every other day or 0.5-0.75mg/kg per day ×

4 weeks. Then the dose is reduced as per the above steroid reduction method, while the dose of MMF or FK506 remains unchanged.

### 5.2.3. Dose adjustment of tacrolimus (FK506)

5.2.3.1 Adjust the dose according to the trough plasma concentration of tacrolimus. If the trough plasma concentration cannot meet the target concentration, it can be increased by adding Wuzhi capsules, ketoconazole, Hexin Shuang or cimetidine. If the trough plasma concentration of tacrolimus is >10 ng/mL, the dose of tacrolimus shall be reduced, and the trough concentration shall be rechecked after one week to adjust the target concentration. After treatment, the increase of SCr ≥ 30% of the basic value and eGFR < 60mL/min.1.73m$^2$ (excluding non-drug factors) shall prompt tacrolimus dose reduction by 50%. If the concentration still fails to reach the target after tacrolimus dose adjustment for one month, the subject shall withdraw from the trial.

5.2.3.2 If the trough plasma concentration of tacrolimus in visit 7 is ≥ 5 ng/mL, the dose of tacrolimus needs to be reduced by 20%-30%, and the trough concentration shall be rechecked in visit 8. If the trough concentration of tacrolimus is still ≥ 5 ng/mL in visit 8, it is necessary to continue to reduce the dose of tacrolimus by 20%-30%, and recheck this concentration in visit 9.

### 5.2.4. Dose adjustment of mycophenolate mofetil (MMF)

5.2.4.1 Adjust the dose according to the plasma concentration of mycophenolic acid. If MPA-AUC fails to meet the target, increase the oral dose of mycophenolate mofetil, but the maximum dose shall not exceed 1.0g each time, BID. Recheck MPA-AUC after 1 week and adjust the drug administration to meet the target concentration. If the patient has blood neutrophils less than 1300 per μL or total leukocytes less than 3000 per μL, diarrhea or severe infection, the dose of mycophenolate mofetil needs to be reduced by 25%. If the side effect persists, it shall be adjusted to half of the original dose. If the case of mycophenolate mofetil dose adjustment, the plasma concentration shall be measured according to the judgment of the investigators. If the concentration still fails to reach the target after mycophenolate mofetil dose adjustment for one month, the subject shall withdraw from the trial.

5.2.4.2 If MPA-AUC is ≥ 40 ng/mL in visit 6, the dose of mycophenolate mofetil needs to be reduced by 20%-30%. After that, retest is no longer required.

### 5.3. concomitant medication

- Statins are allowed for use during this research, which can be increased or decreased according to the blood lipid status.
- Various anticoagulant drugs are allowed for use during this research.
- Immunosuppressants other than the test drug are prohibited for use.
- Plasma exchange and biological agents are prohibited for treatment.
- IVIG can be used during this research, but the reason needs to be recorded.
- When hypertension occurs during treatment, angiotensin converting enzyme inhibitor (ACEi) or angiotensin II receptor blocker (ARB) cannot be used, but dihydropyridine calcium antagonists, β-receptor blocker, diuretic and/or α-receptor blocker can be used for control, with a target blood pressure of: <120/80 mmHg.
- Liver protection drugs are allowed for use during this research.
- If blood glucose rises during treatment, the blood glucose shall be actively controlled with hypoglycemic drugs.
- Various Chinese herbal medicines are prohibited for the treatment of kidney diseases during this research.
- The investigators shall ask the subjects to bring all the drugs they are taking at the time of follow-up, so as to check the concomitant medication for the subjects. For the other drugs or treatments that must be continued for the complicated diseases, the drug name (or other treatment name), dosage, times of use and time must be recorded on the Clinical Study Report for analysis and reporting during summary.

### 5.4. Medication adherence

The medication adherence is calculated as follows, and shall be ranging from 80% to 120%:

$$\text{adherence} = \frac{\text{actual administration}}{\quad} \times 100\%$$

planned administration

# 6. Evaluation of efficacy and safety

## 6.1. Primary efficacy outcomes

1-year relapse-free survival

## 6.2. Efficacy evaluation

### 6.2.1. Primary observation outcomes: 1-year relapse-free survival

### 6.2.2. Secondary observation outcomes:

(1) Frequency of relapses; the time of the first relapse after enrollment; relapse-free survival in the first six months after enrollment;

(2) Renal function: Serum creatinine and glomerular filtration rate (eGFR, see Appendix 1 for the calculation method)

(3) Cumulative steroid dosage: The total amount of steroids taken during this research and the total amount of steroids in the first half of the year

(4) Others: Blood pressure (see Appendix 2 for details), height, weight, blood lipid, hemoglobin, blood albumin, urinary protein/creatinine (morning urine), etc.

(5) Adverse reaction

(6) 24hr urinary protein quantification (applicable to subjects > 3 years old)

### 6.2.3. Efficacy criteria

Complete remission (CR): Completely normal in blood biochemistry test and urine examination;

Partial remission (PR): Urinary protein positive < (+++);

No remission (NR): Urinary protein positive ≥ (+++).

### 6.2.4. Relapse criteria

For 3 consecutive days, morning urinary protein changes from negative to (+++) or (++++), or 24hr urinary protein quantification ≥ 50 mg/kg, or urinary protein/creatinine (mg/mg) ≥2.0.

### 6.3. Safety evaluation

Changes of vital signs: Respiration and heart rate.
Various indexes in laboratory examination: Blood and urine routine examination, liver function, renal function, ECG, chest X-ray examination.
Clinically reported adverse events and adverse reactions.

# 7. Statistical analysis

### 7.1. Analysis data set

Full analysis set (FAS): It refers to the set of eligible cases and drop-off cases. The cases are required to have evaluation data after at least one treatment, but excluding the eliminated cases. Per-protocol set (PPS): It refers to the set of cases that meet the inclusion criteria, do not meet the exclusion criteria and complete the treatment plan, that is, the analysis of cases that meet the trial protocol, have good compliance and complete the specified content in CRF (PP analysis).
Safety set (SS): It refers to the actual data with safety index records after receipt of at least one treatment. The missed safety values shall not be carried forward. It includes some eliminated cases that can be evaluated, such as those whose age exceeds the inclusion criteria, but excludes those whose safety judgment cannot be made due to the use of banned drugs. For the incidence of adverse reactions, the number of cases in the safety set is used as the denominator.

### 7.2. General considerations of date analyses

#### 7.2.1. General Statistical methods

SAS 9.4 software is adopted for Statistical analysis. Two-sided test is adopted for all Statistical tests. If the P value is less than 0.05, the tested difference will be considered “Statistically significant” (unless otherwise specified).
**Descriptive analysis:** Sort out and summarize a large number of data obtained from the survey to find out the internal law of these data - centralized trend and decentralized trend. Single factor analysis is mainly carried out with the help of statistics represented by various data, such as mean and percentage.

### 7.2.2. Statistical inference method

- The description of quantitative indicators includes the calculation of the mean, standard deviation, median, minimum value, maximum value, lower quartile (Q1), upper quartile (Q3), as well as the number and percentage of various types of classification indicators.
- In the comparison of the general conditions between the two groups, appropriate methods shall be adopted for analysis according to the type of indicators. The group t-test or Wilcoxon rank sum test is used for the comparison of quantitative data between two groups; Chi-square test or exact probability method is used for classification data; Wilcoxon rank sum test or CMH test is used for hierarchical data.
- The report is mainly expressed in tables: The tables are self-evident, that is, they have table titles, table notes and number of cases.

## 8. Data handling conventions

This study does not present issues with multiplicity testing.

## 9. Data conversion:

Age (years) = (date of signing of informed consent form - date of birth + 1) / 365.25, rounded down.

Drug-related definitions are: definitely related, probably related, and possibly related.

Missing date: Since it is survival analysis data, it does not need to be filled. Missing text-type data is displayed as "" in the list. Numeric data is missing from the list with "." to display. If the data is recorded as "not applicable"/"NA" and "unable to assess"/"NK", the data will be displayed as raw records when described in the inventory, and processed as missing data for Statistical analysis.

Outlier: In general, all assay results will be used for Statistical analysis. If there is a clear outlier caused by special reasons and affects the Statistical analysis, it is missing and marked in the Statistical analysis results/report.

## 9.1. Content of Statistical analysis

### 9.1.1. Basic value equilibrium analysis

The demographic characteristics, general conditions and baseline conditions (basic value indicators) of the two groups are analyzed to measure the comparability of the two groups. Group t-test or Wilcoxon rank sum test is used for measurement data; Wilcoxon rank sum test is used for classification or grade data; $\chi^2$ test or Fisher test is used for classification data.

### 9.1.2. Effectiveness analysis

All effectiveness analysis/global indicators will be based on ITT principles, and FAS is adopted, for only the adjudicated endpoint events. The investigators are required to report all the potential endpoint events in eCRF.

### 9.1.3. Primary efficacy analysis

Logrank test is used for the analysis of relapse-free survival rate which is then used as a covariate for stratification by Sites. HR and its 95% confidence interval (CI) are calculated with Cox proportional hazard model to estimate the efficacy, the confidence interval is applied to verify the superiority of the experimental group and the control group, with a cut-off value of 1 (risk ratio). The Kaplan-Meier curve of relapse-free survival is drawn by treatment groups. The 1-year relapse-free survival rate and its confidence interval are then calculated.

**Calculation rules for the main efficacy indicators:**

- If relapse occurred within 9 visits (within 364±7 days) or unscheduled visits within 365 days of randomization, defined as the occurrence of endpoint events. Time of relapse = date of relapse - Random date + 1, calculated by days.
- If the visit stipulated in the protocol was not completed within 9 visits (within 364±7 days) due to loss of follow-up midway or withdrawal or other reasons, it is defined as censoring. Time of censoring = date of withdrawal - random date + 1, calculated by days.
- If the visit stipulated in the protocol was not completed within 9 visits (within 364±7 days) due to death in the trial, it is defined as censoring. Time of censoring = Death date - random date + 1, calculated by days.

- If the visit stipulated in the protocol was completed and there is no relapse within 9 visits (within 364±7 days), it is defined as censoring. Time of censoring = date of visit 9 (within 364±7 days) - random date + 1, calculated by days.

### 9.1.4. Secondary efficacy analysis

Secondary outcomes (renal function; cumulative steroid dosage; blood pressure; height; body weight; blood lipids; hemoglobin; serum albumin; urinary protein/creatinine (morning urine); 24hr urinary protein quantification (applicable to subjects > 3 years old); frequency of relapses; the time of the first relapse after enrollment; relapse-free rate in the first six months after enrollment) are described and compared with use of the above general Statistical methods, and the adverse reactions are analyzed by descriptive analysis.

### 9.1.5. Other analysis

(1) Case distribution: Different data sets in each group, case distribution in each Site, comparison of total drop-off rate, detailed list of termination reasons, and descriptive analysis.

(2) Compliance analysis: Compare whether the two groups of patients take the test drugs on time and in quantity, whether they take the drugs and foods prohibited in the Protocol, and conduct the same descriptive analysis as well as $\chi^2$ test or Fisher test for analysis.

(3) The incidence of drug withdrawal or drug change due to adverse reactions in the clinical trial shall be analyzed with $\chi^2$ test or Fisher test. The efficacy in the long-term follow-up after the trial is evaluated by descriptive analysis and $\chi^2$ test or Fisher test.

### 9.1.6. Safety analysis

Safety analysis mainly adopts safety set. All adverse events are coded by MedDRA 20.0 to describe the total number and incidence of adverse events (including serious adverse events), and the frequency table is used to describe the frequency, severity of each adverse event and its relationship with the treatment. Adverse events relevant (related, probably related, and possibly relayed) to this research treatment are described and listed separately. Other safety indicators will also be described and summarized. The laboratory changes with clinical significance will be counted as AE.

### 9.1.7. Subgroup Analysis

Subgroup analyses will be conducted based on the following baseline information:

（1）Gender;

（2）Age at enrollment: Toddlerhood: under 4 years old, not including 4 years old; Preschool age: 4 years old (including 4 years old) to under 7 years old; School age and above: 7 years old and above (including 7 years old).

（3）Age at onset: Toddlerhood: under 4 years old, not including 4 years old; Preschool age: 4 years old (including 4 years old) to under 7 years old; School age and above: 7 years old and above (including 7 years old).

（4）Duration of disease: less than 1 year, 1 year or more.

### 9.1.8 Sensitivity analysis

(1) Sensitivity analysis 1: Based on FAS, the Cox proportional hazards model was used to calculate HR and its 95% confidence interval (CI) to estimate efficacy, with adjusted factors including age, gender, age of onset, and course of kidney disease (less than 1 year, greater than or equal to 1 year).

(2) Sensitivity analysis 2: Based on FAS, the Cox proportional hazards model was used to calculate HR and its 95% confidence interval (CI) to estimate efficacy, with adjusted factors being center, age, gender, age of onset, and course of kidney disease (less than 1 year, greater than or equal to 1 year).

(3) Sensitivity analysis 3: Based on PPS, the Cox proportional hazards model was used to calculate HR and its 95% confidence interval (CI) to estimate efficacy. The adjusted factors were age, gender, age of onset, and duration of kidney disease (less than 1 year, greater than or equal to 1 year).

(4) Sensitivity analysis 4: Based on PPS, the Cox proportional hazards model was used to calculate HR and its 95% confidence interval (CI) to estimate efficacy. The adjusted factors were center, age, gender, age of onset, and course of kidney disease (less than 1 year, greater than or equal to 1 year).

# 10. Statistical analysis results

## 10.1. Research overview

Table 1. Distribution of subjects at each site (All enrolled subjects)

Note: 1. The Site name is not displayed in following table, only the site number. Same below. 2. No. of subjects completed = No. of subjects enrolled - No. of subjects drop-out. Elimination refers

| Research Site | Group | No. of subjects enrolled | No. of subjects drop-out | Drop-out rate | No. of subjects eliminated | Elimination rate | No. of subjects completed |
|---|---|---|---|---|---|---|---|
| Site 1 | Experimental group | | | | | | |
| | Control group | | | | | | |
| | Total | | | | | | |
| Site 2 | Experimental group | | | | | | |
| | Control group | | | | | | |
| | Total | | | | | | |
| … | | | | | | | |
| Total | Experimental group | | | | | | |
| | Control group | | | | | | |
| | Total | | | | | | |

to subjects who complete the trial, but were not included in the PPS. Subjects who withdrew due to the endpoint event were considered to have completed the trial. 3. Drop-out rate = No. of subjects drop-out / No. of subjects enrolled; Elimination rate = No. of subjects eliminated / No. of subjects enrolled.

Table 2. Distribution of subjects at each site

| | PPS | | | FAS | | | SS | | |
|---|---|---|---|---|---|---|---|---|---|
| Site | Experimental group | Control group | Total | Experimental group | Control group | Total | Experimental group | Control group | Total |
| Site 1 | | | | | | | | | |
| Site 2 | | | | | | | | | |
| Total | | | | | | | | | |

Table 3. Trial completion and distribution of analysis data set (All enrolled subjects)

| Items | Indicator | Experimental group | Control group | Total |
|---|---|---|---|---|
| No. of subjects enrolled | Yes | | | |
| | No | | | |
| Complete 1 week follow-up | Yes | | | |
| | No | | | |
| Complete 4 week follow-up | Yes | | | |
| | No | | | |
| Complete 8 week follow-up | Yes | | | |
| | No | | | |
| Complete 16 week follow-up | Yes | | | |
| | No | | | |
| Complete 24 week follow-up | Yes | | | |
| | No | | | |
| Complete 32 week follow-up | Yes | | | |
| | No | | | |
| Complete 40 week follow-up | Yes | | | |
| | No | | | |
| Complete 52 week follow-up | Yes | | | |
| | No | | | |
| Trial completion | Yes | | | |

| Items | Indicator | Experimental group | Control group | Total |
|---|---|---|---|---|
| | No | | | |
| Treatment discontinuation reason | Subject voluntarily withdrew prematurely | | | |
| | Decision by the investigator | | | |
| | Poor compliance that decided by the investigator | | | |
| | Meet the exclusion criteria, or did not meet the inclusion criteria | | | |
| | Refusal to continue trial participation | | | |
| | Lack of efficacy | | | |
| | Adverse events | | | |
| | Lost to follow-up | | | |
| | Death | | | |
| | Other | | | |
| FAS | | | | |
| PPS | | | | |
| SS | | | | |

Note: Follow-up time (years) = (last visit date - random date + 1) / 365.25. Complete 1 week follow-up: follow-up duration ⩾ 1 week, and so on.

## 10.2. Demographic characteristics (FAS)

Table 4. Baseline demographics

| Items | Indicator | Experimental group | Control group | Total |
|---|---|---|---|---|
| Age (years) | N (Missing) | | | |
| | Mean (SD) | | | |
| | Median | | | |
| | Q1, Q3 | | | |
| | Min, Max | | | |
| | Statistics | | | |
| | P-value | | | |
| Gender | male n (%) | | | |

| Items | Indicator | Experimental group | Control group | Total |
|---|---|---|---|---|
| | Female n (%) | | | |
| | Total (Missing) | | | |
| | Statistics | | | |
| | P-value | | | |
| Ethnicity | Han n (%) | | | |
| | Others n (%) | | | |
| | Total (Missing) | | | |
| | Statistics | | | |
| | P-value | | | |
| Source of disease | Outpatient n (%) | | | |
| | Ward n (%) | | | |
| | Total (Missing) | | | |
| | Statistics | | | |
| | P-value | | | |

Table 5. Baseline vital signs

| Items | Indicator | Experimental group | Control group | Total |
|---|---|---|---|---|
| Height (cm) | N (Missing) | | | |
| | Mean (SD) | | | |
| | Median | | | |
| | Q1, Q3 | | | |
| | Min, Max | | | |
| | Statistics | | | |
| | P-value | | | |
| Weight (kg) | N (Missing) | | | |
| | Mean (SD) | | | |
| | Median | | | |
| | Q1, Q3 | | | |

| Items | Indicator | Experimental group | Control group | Total |
| --- | --- | --- | --- | --- |
| | Min, Max | | | |
| | Statistics | | | |
| | P-value | | | |
| BMI (kg/m$^2$) | N (Missing) | | | |
| | Mean (SD) | | | |
| | Median | | | |
| | Q1, Q3 | | | |
| | Min, Max | | | |
| | Statistics | | | |
| | P-value | | | |
| Temperature (℃) | N (Missing) | | | |
| | Mean (SD) | | | |
| | Median | | | |
| | Q1, Q3 | | | |
| | Min, Max | | | |
| | Statistics | | | |
| | P-value | | | |
| Systolic blood pressure (mmHg) | N (Missing) | | | |
| | Mean (SD) | | | |
| | Median | | | |
| | Q1, Q3 | | | |
| | Min, Max | | | |
| | Statistics | | | |
| | P-value | | | |
| Diastolic blood pressure (mmHg) | N (Missing) | | | |
| | Mean (SD) | | | |
| | Median | | | |
| | Q1, Q3 | | | |

| Items | Indicator | Experimental group | Control group | Total |
|---|---|---|---|---|
| | Min, Max | | | |
| | Statistics | | | |
| | P-value | | | |
| Heart rate (times/min) | N (Missing) | | | |
| | Mean (SD) | | | |
| | Median | | | |
| | Q1, Q3 | | | |
| | Min, Max | | | |
| | Statistics | | | |
| | P-value | | | |
| Respiratory rate (times/minute) | N (Missing) | | | |
| | Mean (SD) | | | |
| | Median | | | |
| | Q1, Q3 | | | |
| | Min, Max | | | |
| | Statistics | | | |
| | P-value | | | |

Table 6. Family history and personal history

| Items | Indicator | Experimental group | Control group | Total |
|---|---|---|---|---|
| Family history | No n (%) | | | |
| | Yes n (%) | | | |
| | Total (Missing) | | | |
| | Statistics | | | |
| | P-value | | | |
| History of drug allergy | No n (%) | | | |
| | Yes n (%) | | | |
| | Total (Missing) | | | |

| Items | Indicator | Experimental group | Control group | Total |
|---|---|---|---|---|
| | Statistics | | | |
| | P-value | | | |
| History of surgery | No n (%) | | | |
| | Yes n (%) | | | |
| | Total (Missing) | | | |
| | Statistics | | | |
| | P-value | | | |
| History of pet ownership | No n (%) | | | |
| | Yes n (%) | | | |
| | Total (Missing) | | | |
| | Statistics | | | |
| | P-value | | | |
| Number of gestation | N (Missing) | | | |
| | Mean (SD) | | | |
| | Median | | | |
| | Q1, Q3 | | | |
| | Min, Max | | | |
| | Statistics | | | |
| | P-value | | | |
| Number of parturition | N (Missing) | | | |
| | Mean (SD) | | | |
| | Median | | | |
| | Q1, Q3 | | | |
| | Min, Max | | | |
| | Statistics | | | |
| | P-value | | | |
| Birth weight (kg) | N (Missing) | | | |
| | Mean (SD) | | | |

| Items | Indicator | Experimental group | Control group | Total |
|---|---|---|---|---|
| | Median | | | |
| | Q1, Q3 | | | |
| | Min, Max | | | |
| | Statistics | | | |
| | P-value | | | |
| History of dystocia | No n (%) | | | |
| | Yes n (%) | | | |
| | Total (Missing) | | | |
| | Statistics | | | |
| | P-value | | | |
| Feeding | Mixed feeding n (%) | | | |
| | Single feeding n (%) | | | |
| | Total (Missing) | | | |
| | Statistics | | | |
| | P-value | | | |
| Partial eclipse | No n (%) | | | |
| | Yes n (%) | | | |
| | Total (Missing) | | | |
| | Statistics | | | |
| | P-value | | | |
| History of growth and development | Normal n (%) | | | |
| | Abnormal n (%) | | | |
| | Total (Missing) | | | |
| | Statistics | | | |
| | P-value | | | |
| Vaccination history | No n (%) | | | |
| | Yes n (%) | | | |
| | Total (Missing) | | | |

| Items | Indicator | Experimental group | Control group | Total |
|---|---|---|---|---|
| | Statistics | | | |
| | P-value | | | |

Table 7. History of other diseases, concomitant medications and nephrotic syndrome

| Items | Indicator | Experimental group | Control group | Total |
|---|---|---|---|---|
| History of other diseases and concomitant medications | No n (%) | | | |
| | Yes n (%) | | | |
| | Total (Missing) | | | |
| | Statistics | | | |
| | P-value | | | |
| History of nephrotic syndrome (years) | N (Missing) | | | |
| | Mean (SD) | | | |
| | Median | | | |
| | Q1, Q3 | | | |
| | Min, Max | | | |
| | Statistics | | | |
| | P-value | | | |
| History of nephrotic syndrome (Number of relapses in the past 1 year) | N (Missing) | | | |
| | Mean (SD) | | | |
| | Median | | | |
| | Q1, Q3 | | | |
| | Min, Max | | | |
| | Statistics | | | |
| | P-value | | | |
| History of nephrotic syndrome (Number of relapses in the past 6 months) | N (Missing) | | | |
| | Mean (SD) | | | |
| | Median | | | |
| | Q1, Q3 | | | |

| Items | Indicator | Experimental group | Control group | Total |
|---|---|---|---|---|
| | Min, Max | | | |
| | Statistics | | | |
| | P-value | | | |

Note: History of other diseases and concomitant medications: history of diseases other than the nephrotic syndrome, such as pneumonia, cardiovascular, diabetes, purpura and enuresis, etc.

Table 8. Visual palpation of physical examination Items

| Items | Indicator | Experimental group | Control group | Total |
|---|---|---|---|---|
| Heart | Normal n (%) | | | |
| | Abnormal but not clinically significant n (%) | | | |
| | Abnormal and clinically significant n (%) | | | |
| | Not tested n (%) | | | |
| | Total (Missing) | | | |
| | Statistics | | | |
| | P-value | | | |
| Superficial lymph node | Normal n (%) | | | |
| | Abnormal but not clinically significant n (%) | | | |
| | Abnormal and clinically significant n (%) | | | |
| | Not tested n (%) | | | |
| | Total (Missing) | | | |
| | Statistics | | | |
| | P-value | | | |
| Skin and sclera | Normal n (%) | | | |
| | Abnormal but not clinically significant n (%) | | | |
| | Abnormal and clinically significant n (%) | | | |
| | Not tested n (%) | | | |
| | Total (Missing) | | | |
| | Statistics | | | |
| | P-value | | | |

| | |
|---|---|
| Thyroid gland | Normal n (%) |
| | Abnormal but not clinically significant n (%) |
| | Abnormal and clinically significant n (%) |
| | Not tested n (%) |
| | Total (Missing) |
| | Statistics |
| | P-value |
| Limbs and spine | Normal n (%) |
| | Abnormal but not clinically significant n (%) |
| | Abnormal and clinically significant n (%) |
| | Not tested n (%) |
| | Total (Missing) |
| | Statistics |
| | P-value |
| Lung, abdomen and back | Normal n (%) |
| | Abnormal but not clinically significant n (%) |
| | Abnormal and clinically significant n (%) |
| | Not tested n (%) |
| | Total (Missing) |
| | Statistics |
| | P-value |
| Nervous system | Normal n (%) |
| | Abnormal but not clinically significant n (%) |
| | Abnormal and clinically significant n (%) |
| | Not tested n (%) |
| | Total (Missing) |
| | Statistics |
| | P-value |
| Other | Normal n (%) |

| | | | | |
|---|---|---|---|---|
| | Abnormal but not clinically significant n (%) | | | |
| | Abnormal and clinically significant n (%) | | | |
| | Not tested n (%) | | | |
| | Total (Missing) | | | |
| | Statistics | | | |
| | P-value | | | |

Table 9. Diagnostic consultation of physical examination Items

| Items | Indicator | Experimental group | Control group | Total |
|---|---|---|---|---|
| Defecation | Normal n (%) | | | |
| | Abnormal but not clinically significant n (%) | | | |
| | Abnormal and clinically significant n (%) | | | |
| | Not tested n (%) | | | |
| | Total (Missing) | | | |
| | Statistics | | | |
| | P-value | | | |
| Fever/dizziness/vertigo | Normal n (%) | | | |
| | Abnormal but not clinically significant n (%) | | | |
| | Abnormal and clinically significant n (%) | | | |
| | Not tested n (%) | | | |
| | Total (Missing) | | | |
| | Statistics | | | |
| | P-value | | | |
| Dry mouth and eyes | Normal n (%) | | | |
| | Abnormal but not clinically significant n (%) | | | |
| | Abnormal and clinically significant n (%) | | | |
| | Not tested n (%) | | | |
| | Total (Missing) | | | |
| | Statistics | | | |

| | |
|---|---|
| | P-value |
| Gastrointestinal digestion | Normal n (%) |
| | Abnormal but not clinically significant n (%) |
| | Abnormal and clinically significant n (%) |
| | Not tested n (%) |
| | Total (Missing) |
| | Statistics |
| | P-value |
| Myalgia/muscle weakness | Normal n (%) |
| | Abnormal but not clinically significant n (%) |
| | Abnormal and clinically significant n (%) |
| | Not tested n (%) |
| | Total (Missing) |
| | Statistics |
| | P-value |
| Arthralgia/arthritis | Normal n (%) |
| | Abnormal but not clinically significant n (%) |
| | Abnormal and clinically significant n (%) |
| | Not tested n (%) |
| | Total (Missing) |
| | Statistics |
| | P-value |
| Dry/itchy skin | Normal n (%) |
| | Abnormal but not clinically significant n (%) |
| | Abnormal and clinically significant n (%) |
| | Not tested n (%) |
| | Total (Missing) |
| | Statistics |
| | P-value |

| | | | | |
|---|---|---|---|---|
| Other | Normal n (%) | | | |
| | Abnormal but not clinically significant n (%) | | | |
| | Abnormal and clinically significant n (%) | | | |
| | Not tested n (%) | | | |
| | Total (Missing) | | | |
| | Statistics | | | |
| | P-value | | | |

## 10.3. Baseline examinations (FAS)

Table 10. DNA/antibody tests

| Items | Indicator | Experimental group | Control group | Total |
|---|---|---|---|---|
| Blood EBV-DNA | Normal n (%) | | | |
| | Abnormal but not clinically significant n (%) | | | |
| | Abnormal and clinically significant n (%) | | | |
| | Not tested n (%) | | | |
| | Total (Missing) | | | |
| | Statistics | | | |
| | P-value | | | |
| Blood CMV antibody | Normal n (%) | | | |
| | Abnormal but not clinically significant n (%) | | | |
| | Abnormal and clinically significant n (%) | | | |
| | Not tested n (%) | | | |
| | Total (Missing) | | | |
| | Statistics | | | |
| | P-value | | | |
| IgM | Normal n (%) | | | |
| | Abnormal but not clinically significant n (%) | | | |
| | Abnormal and clinically significant n (%) | | | |

| | | | | |
|---|---|---|---|---|
| | Not tested n (%) | | | |
| | Total (Missing) | | | |
| | Statistics | | | |
| | P-value | | | |
| IgG | Normal n (%) | | | |
| | Abnormal but not clinically significant n (%) | | | |
| | Abnormal and clinically significant n (%) | | | |
| | Not tested n (%) | | | |
| | Total (Missing) | | | |
| | Statistics | | | |
| | P-value | | | |

Table 11. Hepatitis virus

| Items | Indicator | Experimental group | Control group | Total |
|---|---|---|---|---|
| HBs-Ag | Normal n (%) | | | |
| | Abnormal but not clinically significant n (%) | | | |
| | Abnormal and clinically significant n (%) | | | |
| | Not tested n (%) | | | |
| | Total (Missing) | | | |
| | Statistics | | | |
| | P-value | | | |
| Anti-HCV | Normal n (%) | | | |
| | Abnormal but not clinically significant n (%) | | | |
| | Abnormal and clinically significant n (%) | | | |
| | Not tested n (%) | | | |
| | Total (Missing) | | | |
| | Statistics | | | |
| | P-value | | | |

Table 12. Examination of mycobacterium tuberculosis and fungi

| Items | Indicator | Experimental group | Control group | Total |
|---|---|---|---|---|
| Mycobacterium tuberculosis test | Normal n (%) | | | |
| | Abnormal but not clinically significant n (%) | | | |
| | Abnormal and clinically significant n (%) | | | |
| | Not tested n (%) | | | |
| | Total (Missing) | | | |
| | Statistics | | | |
| | P-value | | | |
| Fungal examination | Fungal G test n (%) | | | |
| | Fungal other n (%) | | | |
| | Total (Missing) | | | |
| | Statistics | | | |
| | P-value | | | |
| Fungal examination result | Normal n (%) | | | |
| | Abnormal but not clinically significant n (%) | | | |
| | Abnormal and clinically significant n (%) | | | |
| | Not tested n (%) | | | |
| | Total (Missing) | | | |
| | Statistics | | | |
| | P-value | | | |

Table 13. Urine test

| Items | Indicator | Experimental group | Control group | Total |
|---|---|---|---|---|
| 24hr urine protein (>3 years old) | Normal n (%) | | | |
| | Abnormal but not clinically significant n (%) | | | |
| | Abnormal and clinically significant n (%) | | | |
| | Not tested n (%) | | | |
| | Total (Missing) | | | |

| | | | | |
|---|---|---|---|---|
| | Statistics | | | |
| | P-value | | | |
| Urine protein/serum creatinine | Normal n (%) | | | |
| | Abnormal but not clinically significant n (%) | | | |
| | Abnormal and clinically significant n (%) | | | |
| | Not tested n (%) | | | |
| | Total (Missing) | | | |
| | Statistics | | | |
| | P-value | | | |

Table 14. Glomerular filtration rate

| Items | Indicator | Experimental group | Control group | Total |
|---|---|---|---|---|
| Examine or not | No n (%) | | | |
| | Yes n (%) | | | |
| | Total (Missing) | | | |
| | Statistics | | | |
| | P-value | | | |
| eGFR | Normal n (%) | | | |
| | Abnormal but not clinically significant n (%) | | | |
| | Abnormal and clinically significant n (%) | | | |
| | Not tested n (%) | | | |
| | Total (Missing) | | | |
| | Statistics | | | |
| | P-value | | | |

Table 15. Retained biological samples of blood and urine

| Items | Indicator | Experimental group | Control group | Total |
|---|---|---|---|---|
| Retained biological samples of blood and urine | No n (%) | | | |
| | Yes n (%) | | | |

| | | | | |
|---|---|---|---|---|
| | Total (Missing) | | | |
| | Statistics | | | |
| | P-value | | | |

Table 16. Chest X-ray

| Items | Indicator | Experimental group | Control group | Total |
|---|---|---|---|---|
| Result | Normal n (%) | | | |
| | Abnormal but not clinically significant n (%) | | | |
| | Abnormal and clinically significant n (%) | | | |
| | Not tested n (%) | | | |
| | Total (Missing) | | | |
| | Statistics | | | |
| | P-value | | | |

Table 17. ECG examinations

| Items | Indicator | Experimental group | Control group | Total |
|---|---|---|---|---|
| Result | Normal n (%) | | | |
| | Abnormal but not clinically significant n (%) | | | |
| | Abnormal and clinically significant n (%) | | | |
| | Not tested n (%) | | | |
| | Total (Missing) | | | |
| | Statistics | | | |
| | P-value | | | |

Table 18. Primary diagnosis

| Items | Indicator | Experimental group | Control group | Total |
|---|---|---|---|---|
| Primary diagnosis | Steroid-sensitive nephrotic syndrome (SSNS) n (%) | | | |
| | Steroid-resistant nephrotic syndrome (SRNS) n (%) | | | |
| | Secondary nephrotic syndrome n (%) | | | |

| | |
|---|---|
| | Steroid-dependent nephrotic syndrome (SDNS) n (%) |
| | Frequently relapsing nephrotic syndrome (FRNS) n (%) |
| | Other nephrotic syndrome n (%) |
| | Total (Missing) |
| | Statistics |
| | P-value |

## 10.4. Efficacy analysis (FAS/PPS)

### 10.4.1. Primary efficacy indicators: 1-year relapse-free survival rate

Table 19. 1-year relapse-free survival analysis

| | | FAS | | PPS | |
|---|---|---|---|---|---|
| Items | Indicator | Experimental group | Control group | Experimental group | Control group |
| 1-year recurrence time (days) | N (Missing) | | | | |
| | Endpoint event (%) | | | | |
| | Censoring (%) | | | | |
| | Median survival time | | | | |
| | 95%CI | | | | |
| | Q1, Q3 | | | | |
| | Log-rank test | | | | |
| | P-value | | | | |
| Cox regression analysis | Hazard Ratio | | | | |
| | 95%CI | | | | |
| | Wald chi-square | | | | |
| | P-value | | | | |

Note: The independent variables of Cox regression analysis are group and Site. Same below.

Figure 1. 1-year relapse-free survival analysis chart (FAS)

Figure 2. 1-year relapse-free survival analysis chart (PPS)

### 10.4.2. Secondary efficacy indicators

#### 10.4.2.1. The first relapse survival analysis after enrollment

Table 20. The time of the first relapse after enrollment

| | | FAS | | PPS | |
|---|---|---|---|---|---|
| Items | Indicator | Experimental group | Control group | Experimental group | Control group |
| The time of the first relapse after enrollment (days) | N (Missing) | | | | |
| | Mean (SD) | | | | |
| | Median | | | | |
| | Q1, Q3 | | | | |
| | Min, Max | | | | |
| | 95%CI | | | | |
| | P-value | | | | |

Note: The time of first relapse after enrollment (days) = Date of first relapse after enrollment - Random date + 1.

Table 21. The first relapse survival analysis after enrollment

| | | FAS | | PPS | |
|---|---|---|---|---|---|
| Items | Indicator | Experimental group | Control group | Experimental group | Control group |
| The time of the first relapse after enrollment (days) | N (Missing) | | | | |
| | Endpoint event (%)(%) | | | | |
| | Censoring (%) | | | | |
| | Median survival time | | | | |
| | 95%CI | | | | |
| | Q1, Q3 | | | | |
| | Log-rank test | | | | |
| Cox regression analysis | Hazard Ratio | | | | |
| | 95%CI | | | | |
| | Wald Chi-square | | | | |
| | P-value | | | | |

### 10.4.2.2. **6-month relapse-free survival analysis after enrollment**

Table 22. 6-month relapse-free survival analysis after enrollment

| Items | Indicator | FAS | | PPS | |
|---|---|---|---|---|---|
| | | Experimental group | Control group | Experimental group | Control group |
| 6-month recurrence time (days) | N (Missing) | | | | |
| | Endpoint event (%)(%) | | | | |
| | Censoring (%) | | | | |
| | Median survival time | | | | |
| | 95%CI | | | | |
| | Q1, Q3 | | | | |
| | Log-rank test | | | | |
| Cox regression analysis | Hazard Ratio | | | | |
| | 95%CI | | | | |
| | Wald Chi-square | | | | |
| | P-value | | | | |

Figure 3. 6-month relapse-free survival analysis chart (FAS)

Figure 4. 6-month relapse-free survival analysis chart (PPS)

### 10.4.2.3.**Serum renal function**

Table 23. Changes in eGFR relative to baseline at each visit

| | | FAS | | | PPS | | |
|---|---|---|---|---|---|---|---|
| Items | Indicator | Experimental group | Control group | Total | Experimental group | Control group | Total |
| Baseline | N (Missing) | | | | | | |
| | Mean (SD) | | | | | | |
| | Median | | | | | | |
| | Q1, Q3 | | | | | | |
| | Min, Max | | | | | | |
| | 95%CI | | | | | | |
| Between-group comparison in baseline | Statistics | | | | | | |
| | P-value | | | | | | |
| Visit 2/Day 7±3 | N (Missing) | | | | | | |
| | Mean (SD) | | | | | | |
| | Median | | | | | | |
| | Q1, Q3 | | | | | | |
| | Min, Max | | | | | | |
| | 95%CI | | | | | | |
| Between-group comparison in Visit 2/Day 7±3 | Statistics | | | | | | |
| | P-value | | | | | | |
| Change in Visit 2/Day 7±3 from baseline | N (Missing) | | | | | | |
| | Mean (SD) | | | | | | |
| | Median | | | | | | |
| | Q1, Q3 | | | | | | |
| | Min, Max | | | | | | |
| | 95%CI | | | | | | |
| Pre- and post-treatment comparison of intra-group | Statistical method | | | | | | |
| | Statistics | | | | | | |
| | P-value | | | | | | |
| Comparison of changes between groups pre- and post-treatment | Statistics | | | | | | |
| | P-value | | | | | | |
| | N (Missing) | | | | | | |

| Items | Indicator | FAS Experimental group | FAS Control group | FAS Total | PPS Experimental group | PPS Control group | PPS Total |
|---|---|---|---|---|---|---|---|
| Visit 4/Day 56±7 | Mean (SD) | | | | | | |
| | Median | | | | | | |
| | Q1, Q3 | | | | | | |
| | Min, Max | | | | | | |
| | 95%CI | | | | | | |
| Between-group comparison in Visit 4/Day 56±7 | Statistics | | | | | | |
| | P-value | | | | | | |
| Change in Visit 4/Day 56±7 from baseline | N (Missing) | | | | | | |
| | Mean (SD) | | | | | | |
| | Median | | | | | | |
| | Q1, Q3 | | | | | | |
| | Min, Max | | | | | | |
| | 95%CI | | | | | | |
| Pre- and post-treatment comparison of intra-group | Statistical method | | | | | | |
| | Statistics | | | | | | |
| | P-value | | | | | | |
| Comparison of changes between groups pre- and post-treatment | Statistics | | | | | | |
| | P-value | | | | | | |
| Visit 5/Day 112±7 | N (Missing) | | | | | | |
| | Mean (SD) | | | | | | |
| | Median | | | | | | |
| | Q1, Q3 | | | | | | |
| | Min, Max | | | | | | |
| | 95%CI | | | | | | |
| Between-group comparison in Visit 5/Day 112±7 | Statistics | | | | | | |
| | P-value | | | | | | |
| Change in Visit 5/Day 112±7 from baseline | N (Missing) | | | | | | |
| | Mean (SD) | | | | | | |
| | Median | | | | | | |
| | Q1, Q3 | | | | | | |
| | Min, Max | | | | | | |

| Items | Indicator | FAS Experimental group | FAS Control group | FAS Total | PPS Experimental group | PPS Control group | PPS Total |
|---|---|---|---|---|---|---|---|
| | 95%CI | | | | | | |
| Pre- and post-treatment comparison of intra-group | Statistical method | | | | | | |
| | Statistics | | | | | | |
| | P-value | | | | | | |
| Comparison of changes between groups pre- and post-treatment | Statistics | | | | | | |
| | P-value | | | | | | |
| Visit 6/Day 168±7 | N (Missing) | | | | | | |
| | Mean (SD) | | | | | | |
| | Median | | | | | | |
| | Q1, Q3 | | | | | | |
| | Min, Max | | | | | | |
| | 95%CI | | | | | | |
| Between-group comparison in Visit 6/Day 168±7 | Statistics | | | | | | |
| | P-value | | | | | | |
| Change in Visit 6/Day 168±7 from baseline | N (Missing) | | | | | | |
| | Mean (SD) | | | | | | |
| | Median | | | | | | |
| | Q1, Q3 | | | | | | |
| | Min, Max | | | | | | |
| | 95%CI | | | | | | |
| Pre- and post-treatment comparison of intra-group | Statistical method | | | | | | |
| | Statistics | | | | | | |
| | P-value | | | | | | |
| Comparison of changes between groups pre- and post-treatment | Statistics | | | | | | |
| | P-value | | | | | | |
| Visit 7/Day 224±7 | N (Missing) | | | | | | |
| | Mean (SD) | | | | | | |
| | Median | | | | | | |
| | Q1, Q3 | | | | | | |
| | Min, Max | | | | | | |
| | 95%CI | | | | | | |

| Items | Indicator | FAS Experimental group | FAS Control group | FAS Total | PPS Experimental group | PPS Control group | PPS Total |
|---|---|---|---|---|---|---|---|
| Between-group comparison in Visit 7/Day 224±7 | Statistics | | | | | | |
| | P-value | | | | | | |
| Change in Visit 7/Day 224±7 from baseline | N (Missing) | | | | | | |
| | Mean (SD) | | | | | | |
| | Median | | | | | | |
| | Q1, Q3 | | | | | | |
| | Min, Max | | | | | | |
| | 95%CI | | | | | | |
| Pre- and post-treatment comparison of intra-group | Statistical method | | | | | | |
| | Statistics | | | | | | |
| | P-value | | | | | | |
| Comparison of changes between groups pre- and post-treatment | Statistics | | | | | | |
| | P-value | | | | | | |
| Visit 8/Day 280±7 | N (Missing) | | | | | | |
| | Mean (SD) | | | | | | |
| | Median | | | | | | |
| | Q1, Q3 | | | | | | |
| | Min, Max | | | | | | |
| | 95%CI | | | | | | |
| Between-group comparison in Visit 8/Day 280±7 | Statistics | | | | | | |
| | P-value | | | | | | |
| Change in Visit 8/Day 280±7 from baseline | N (Missing) | | | | | | |
| | Mean (SD) | | | | | | |
| | Median | | | | | | |
| | Q1, Q3 | | | | | | |
| | Min, Max | | | | | | |
| | 95%CI | | | | | | |
| Pre- and post-treatment comparison of intra-group | Statistical method | | | | | | |
| | Statistics | | | | | | |
| | P-value | | | | | | |
| | Statistics | | | | | | |

| Items | Indicator | FAS Experimental group | FAS Control group | FAS Total | PPS Experimental group | PPS Control group | PPS Total |
|---|---|---|---|---|---|---|---|
| Comparison of changes between groups pre- and post-treatment | P-value | | | | | | |
| Visit 9/Day 364±7 | N (Missing) | | | | | | |
| | Mean (SD) | | | | | | |
| | Median | | | | | | |
| | Q1, Q3 | | | | | | |
| | Min, Max | | | | | | |
| | 95%CI | | | | | | |
| Between-group comparison in Visit 9/Day 364±7 | Statistics | | | | | | |
| | P-value | | | | | | |
| Change in Visit 9/Day 364±7 from baseline | N (Missing) | | | | | | |
| | Mean (SD) | | | | | | |
| | Median | | | | | | |
| | Q1, Q3 | | | | | | |
| | Min, Max | | | | | | |
| | 95%CI | | | | | | |
| Pre- and post-treatment comparison of intra-group | Statistical method | | | | | | |
| | Statistics | | | | | | |
| | P-value | | | | | | |
| Comparison of changes between groups pre- and post-treatment | Statistics | | | | | | |
| | P-value | | | | | | |

Figure 5. Quantitative variables of eGFR at each visit

Table 24. Change in Scr from baseline at each visit
Ibid. Same below.

Figure 6. Quantitative variables of Scr at each visit

Table 25. Change in serum uric acid from baseline at each visit

Figure 7. Quantitative variables of serum uric acid at each visit

Table 26. Change in blood urea nitrogen from baseline at each visit

Figure 8. Quantitative variables of blood urea nitrogen at each visit

Table 27. Change in serum cystatin C from baseline at each visit

Figure 9. Quantitative variables of serum cystatin C at each visit

#### 10.4.2.4. **Cumulative steroid dosage**

Table 28. Total amount of steroids taken during the study

| Items | Indicator | Experimental group | Control group | Total |
|---|---|---|---|---|
| Total amount of steroids taken during the study | N (Missing) | | | |
| | Mean (SD) | | | |
| | Median | | | |
| | Q1, Q3 | | | |
| | Min, Max | | | |
| | Statistics | | | |
| | P-value | | | |

Table 29. Total amount of steroids taken during 6 months after enrollment

| Items | Indicator | Experimental group | Control group | Total |
|---|---|---|---|---|
| Total amount of steroids taken during 6 months after enrollment | N (Missing) | | | |
| | Mean (SD) | | | |
| | Median | | | |
| | Q1, Q3 | | | |
| | Min, Max | | | |

| Items | Indicator | Experimental group | Control group | Total |
| --- | --- | --- | --- | --- |
| | Statistics | | | |
| | P-value | | | |

### 10.4.2.5. Vital signs

Table 30. Change in systolic blood pressure from baseline at each visit

Table 31. Change in diastolic blood pressure from baseline at each visit

Table 32. Change in height from baseline at each visit

Table 33. Change in weight from baseline at each visit

Table 34. Height percentile

| Items | Indicator | Experimental group | Control group | Total |
| --- | --- | --- | --- | --- |
| Baseline | N (Missing) | | | |
| | Mean (SD) | | | |
| | Median | | | |
| | Q1, Q3 | | | |
| | Min, Max | | | |
| | Statistics | | | |
| | P-value | | | |
| Visit 5/Day 112±7 | N (Missing) | | | |
| | Mean (SD) | | | |
| | Median | | | |
| | Q1, Q3 | | | |
| | Min, Max | | | |
| | Statistics | | | |
| | P-value | | | |
| Visit 9/Day 364±7 | N (Missing) | | | |
| | Mean (SD) | | | |
| | Median | | | |

| Items | Indicator | Experimental group | Control group | Total |
|---|---|---|---|---|
| | Q1, Q3 | | | |
| | Min, Max | | | |
| | Statistics | | | |
| | P-value | | | |

Note: Data from the external file “XX”.

#### 10.4.2.6. **Blood lipid**

Table 35. Change in triglyceride from baseline at each visit

Table 36. Change in total cholesterol from baseline at each visit

#### 10.4.2.7. **Haemoglobin and albumin (ALB)**

Table 37. Change in haemoglobin from baseline at each visit

Table 38. Change in ALB from baseline at each visit

#### 10.4.2.8. **Urine test**

Table 39. Change in 24hr urine protein (>3 years old) from baseline at each visit

Table 40. Change in urine protein/creatinine (morning urine) from baseline at each visit

#### 10.4.2.9. **Serum liver function and fasting blood glucose**

Table 41. Change in glutamyl transpeptidase (GT) from baseline at each visit

Table 42. Change in alanine aminotransferase (ALT) from baseline at each visit

Table 43. Change in aspartate aminotransferase (AST) from baseline at each visit

Table 44. Change in lactate dehydrogenase from baseline at each visit

Table 45. Change in total bilirubin from baseline at each visit

Table 46. Change in direct bilirubin from baseline at each visit

Table 47. Change in alkaline phosphatase from baseline at each visit

Table 48. Change in total protein from baseline at each visit

Table 49. Change in globulin from baseline at each visit

Table 50. Change in fasting blood glucosefrom baseline at each visit

#### 10.4.2.10. **Blood routine examination**

Table 51. Change in leukocyte count (WBC) from baseline at each visit

Table 52. Change in erythrocyte count (RBC) from baseline at each visit

Table 53. Change in platelet count (PLT) from baseline at each visit

Table 54. Change in lymphocyte percentage (LY) from baseline at each visit

#### 10.4.2.11. Urine routine examination

Table 55. Change in leukocyte from baseline at each visit

Table 56. Change in erythrocyte from baseline at each visit

Table 57. Change in pH from baseline at each visit

Table 58. Change in urine protein from baseline at each visit

#### 10.4.2.12. Plasma concentration

Table 59. Change in tacrolimus concentration in plasma from baseline at each visit

Table 60. Change in mycophenolic acid concentration in plasma from baseline at each visit

### 10.4.3. Disease remission

Table 61. Disease remission at each visit

| Items | Indicator | Experimental group | Control group | Total |
|---|---|---|---|---|
| Visit 1/Day 0 | Complete remission (CR) n (%) | | | |
| | Partial remission (PR) n (%) | | | |
| | Non remission (NR) n (%) | | | |
| | Total (Missing) | | | |
| | Statistics | | | |
| | P-value | | | |
| Visit 2/Day 7±3 | Complete remission (CR) n (%) | | | |

| | |
|---|---|
| | Partial remission (PR) n (%) |
| | Non remission (NR) n (%) |
| | Total (Missing) |
| | Statistics |
| | P-value |
| … | |

## 10.5. Safety analysis (SS)

### 10.5.1. Adverse event (AE)

Table 62. Between-group comparison of AE

| Items | Experimental group | | | Control group | | | P-value |
|---|---|---|---|---|---|---|---|
| | No. of AEs | No. of subjects | Percentage | No. of AEs | No. of subjects | Percentage | |
| AE | | | | | | | |
| AE of severe severity | | | | | | | |
| Drug-related AE | | | | | | | |
| Serious adverse event (SAE) | | | | | | | |
| Drug-related SAE | | | | | | | |
| Drop-out due to AE | | | | | | | |
| Drop-out due to SAE | | | | | | | |

Note 1: The definitions related to drugs are “definitely related”, “probably related”, “possibly related”, “unlikely related” and “not related”.

Note 2: The data of SAE that cause the drop-out came from the external file “XX”.

Table 63. characteristics of AE in each group

| Items | | Experimental group | | | Control group | | | P-value |
|---|---|---|---|---|---|---|---|---|
| | | No. of AEs | No. of subjects | Incidence | No. of AEs | No. of subjects | Incidence | |
| Relationship with study drug | Definitely related | | | | | | | |
| | Probably related | | | | | | | |

| Items | | Experimental group | | | Control group | | | P-value |
|---|---|---|---|---|---|---|---|---|
| | | No. of AEs | No. of subjects | Incidence | No. of AEs | No. of subjects | Incidence | |
| | Possibly related | | | | | | | |
| | Unlikely related | | | | | | | |
| | Not related | | | | | | | |
| Outcome | Recovered/resolved | | | | | | | |
| | Recovered/resolved with sequelae | | | | | | | |
| | Not recovered | | | | | | | |
| | Recovering/resolving | | | | | | | |
| | Death | | | | | | | |
| | Unknown | | | | | | | |

Table 64. Between-group comparison of SAE

| Items | Experimental group (n=XX) | | | Control group (n=XX) | | | Total (n=XX) | | | P-value |
|---|---|---|---|---|---|---|---|---|---|---|
| | No. of AEs | No. of subjects | Incidence (%) | No. of AEs | No. of subjects | Incidence (%) | No. of AEs | No. of subjects | Incidence (%) | |
| Results in death | | | | | | | | | | |
| Requires hospitalization | | | | | | | | | | |
| Prolongs hospitalization | | | | | | | | | | |
| Disability | | | | | | | | | | |
| Dysfunction | | | | | | | | | | |
| Congenital malformation | | | | | | | | | | |
| Life threatening | | | | | | | | | | |
| Other | | | | | | | | | | |

Table 65. Summary of AE base on SOC and PT coding

| Indicator | Experimental group (n=XX) | | | Control group (n=XX) | | | Total (n=XX) | | | |
|---|---|---|---|---|---|---|---|---|---|---|
| | No. of AEs | No. of subjects | Incidence (%) | No. of AEs | No. of subjects | Incidence (%) | No. of AEs | No. of subjects | Incidence (%) | P-value |
| AE | | | | | | | | | | |

| Indicator | Experimental group (n=XX) | | | Control group (n=XX) | | | Total (n=XX) | | | |
|---|---|---|---|---|---|---|---|---|---|---|
| | No. of AEs | No. of subjects | Incidence (%) | No. of AEs | No. of subjects | Incidence (%) | No. of AEs | No. of subjects | Incidence (%) | P-value |
| SOC1 | | | | | | | | | | |
| PT1 | | | | | | | | | | |
| PT2 | | | | | | | | | | |
| … | | | | | | | | | | |
| SOC2 | | | | | | | | | | |
| PT1 | | | | | | | | | | |
| PT2 | | | | | | | | | | |
| … | | | | | | | | | | |

Table 66. Summary of adverse reactions base on SOC and PT coding

| Indicator | Experimental group (n=XX) | | | Control group (n=XX) | | | Total (n=XX) | | | |
|---|---|---|---|---|---|---|---|---|---|---|
| | No. of AEs | No. of subjects | Incidence (%) | No. of AEs | No. of subjects | Incidence (%) | No. of AEs | No. of subjects | Incidence (%) | P-value |
| Adverse event | | | | | | | | | | |
| SOC1 | | | | | | | | | | |
| PT1 | | | | | | | | | | |
| PT2 | | | | | | | | | | |
| … | | | | | | | | | | |
| SOC2 | | | | | | | | | | |
| PT1 | | | | | | | | | | |
| PT2 | | | | | | | | | | |
| … | | | | | | | | | | |

## 10.5.2. Vital signs

Table 67. Change in heart rate from baseline at each visit

| Items | Indicator | Experimental group | Control group | Total |
|---|---|---|---|---|
| Baseline | N (Missing) | | | |
| | Mean (SD) | | | |
| | Median | | | |
| | Q1, Q3 | | | |
| | Min, Max | | | |
| | 95%CI | | | |
| Between-group comparison in baseline | Statistics | | | |
| | P-value | | | |
| Visit 2/Day 7±3 | N (Missing) | | | |
| | Mean (SD) | | | |
| | Median | | | |
| | Q1, Q3 | | | |
| | Min, Max | | | |
| | 95%CI | | | |
| Between-group comparison in Visit 2/Day 7±3 | Statistics | | | |
| | P-value | | | |
| Change in Visit 2/Day 7±3 from baseline | N (Missing) | | | |
| | Mean (SD) | | | |
| | Median | | | |
| | Q1, Q3 | | | |
| | Min, Max | | | |
| | 95%CI | | | |
| Pre- and post-treatment comparison of intra-group | Statistical method | | | |
| | Statistics | | | |
| | P-value | | | |
| Comparison of changes between groups pre- and post-treatment | Statistics | | | |
| | P-value | | | |
| Visit 4/Day 56±7 | N (Missing) | | | |
| | Mean (SD) | | | |

| Items | Indicator | Experimental group | Control group | Total |
|---|---|---|---|---|
| | Median | | | |
| | Q1, Q3 | | | |
| | Min, Max | | | |
| | 95%CI | | | |
| Between-group comparison in Visit 4/Day 56±7 | Statistics | | | |
| | P-value | | | |
| Change in Visit 4/Day 56±7 from baseline | N (Missing) | | | |
| | Mean (SD) | | | |
| | Median | | | |
| | Q1, Q3 | | | |
| | Min, Max | | | |
| | 95%CI | | | |
| Pre- and post-treatment comparison of intra-group | Statistical method | | | |
| | Statistics | | | |
| | P-value | | | |
| Comparison of changes between groups pre- and post-treatment | Statistics | | | |
| | P-value | | | |
| Visit 5/Day 112±7 | N (Missing) | | | |
| | Mean (SD) | | | |
| | Median | | | |
| | Q1, Q3 | | | |
| | Min, Max | | | |
| | 95%CI | | | |
| Between-group comparison in Visit 5/Day 112±7 | Statistics | | | |
| | P-value | | | |
| Change in Visit 5/Day 112±7 from baseline | N (Missing) | | | |
| | Mean (SD) | | | |
| | Median | | | |
| | Q1, Q3 | | | |
| | Min, Max | | | |
| | 95%CI | | | |
| | Statistical method | | | |

| Items | Indicator | Experimental group | Control group | Total |
|---|---|---|---|---|
| Pre- and post-treatment comparison of intra-group | Statistics | | | |
| | P-value | | | |
| Comparison of changes between groups pre- and post-treatment | Statistics | | | |
| | P-value | | | |
| Visit 6/Day 168±7 | N (Missing) | | | |
| | Mean (SD) | | | |
| | Median | | | |
| | Q1, Q3 | | | |
| | Min, Max | | | |
| | 95%CI | | | |
| Between-group comparison in Visit 6/Day 168±7 | Statistics | | | |
| | P-value | | | |
| Change in Visit 6/Day 168±7 from baseline | N (Missing) | | | |
| | Mean (SD) | | | |
| | Median | | | |
| | Q1, Q3 | | | |
| | Min, Max | | | |
| | 95%CI | | | |
| Pre- and post-treatment comparison of intra-group | Statistical method | | | |
| | Statistics | | | |
| | P-value | | | |
| Comparison of changes between groups pre- and post-treatment | Statistics | | | |
| | P-value | | | |
| Visit 7/Day 224±7 | N (Missing) | | | |
| | Mean (SD) | | | |
| | Median | | | |
| | Q1, Q3 | | | |
| | Min, Max | | | |
| | 95%CI | | | |
| Between-group comparison in Visit 7/Day 224±7 | Statistics | | | |
| | P-value | | | |
| | N (Missing) | | | |

| Items | Indicator | Experimental group | Control group | Total |
|---|---|---|---|---|
| Change in Visit 7/Day 224±7 from baseline | Mean (SD) | | | |
| | Median | | | |
| | Q1, Q3 | | | |
| | Min, Max | | | |
| | 95%CI | | | |
| Pre- and post-treatment comparison of intra-group | Statistical method | | | |
| | Statistics | | | |
| | P-value | | | |
| Comparison of changes between groups pre- and post-treatment | Statistics | | | |
| | P-value | | | |
| Visit 8/Day 280±7 | N (Missing) | | | |
| | Mean (SD) | | | |
| | Median | | | |
| | Q1, Q3 | | | |
| | Min, Max | | | |
| | 95%CI | | | |
| Between-group comparison in Visit 8/Day 280±7 | Statistics | | | |
| | P-value | | | |
| Change in Visit 8/Day 280±7 from baseline | N (Missing) | | | |
| | Mean (SD) | | | |
| | Median | | | |
| | Q1, Q3 | | | |
| | Min, Max | | | |
| | 95%CI | | | |
| Pre- and post-treatment comparison of intra-group | Statistical method | | | |
| | Statistics | | | |
| | P-value | | | |
| Comparison of changes between groups pre- and post-treatment | Statistics | | | |
| | P-value | | | |
| Visit 9/Day 364±7 | N (Missing) | | | |
| | Mean (SD) | | | |
| | Median | | | |

| Items | Indicator | Experimental group | Control group | Total |
|---|---|---|---|---|
| | Q1, Q3 | | | |
| | Min, Max | | | |
| | 95%CI | | | |
| Between-group comparison in Visit 9/Day 364±7 | Statistics | | | |
| | P-value | | | |
| Change in Visit 9/Day 364±7 from baseline | N (Missing) | | | |
| | Mean (SD) | | | |
| | Median | | | |
| | Q1, Q3 | | | |
| | Min, Max | | | |
| | 95%CI | | | |
| Pre- and post-treatment comparison of intra-group | Statistical method | | | |
| | Statistics | | | |
| | P-value | | | |
| Comparison of changes between groups pre- and post-treatment | Statistics | | | |
| | P-value | | | |

Table 68. Change in respiratory rate from baseline at each visit

Ibid.

### 10.5.3. Blood routine examination

Table 69. Summary of blood routine examination-Leukocyte count (WBC)

| Items | Indicator | Experimental group | Control group | Total |
|---|---|---|---|---|
| Visit 1/Day 0 | Within the normal range n (%) | | | |
| | Below the normal range n (%) | | | |
| | Higher than the normal range n (%) | | | |
| | Total (Missing) | | | |
| | Statistics | | | |
| | P-value | | | |

| | | | | |
|---|---|---|---|---|
| Visit 9/Day 364±7 | Within the normal range n (%) | | | |
| | Below the normal range n (%) | | | |
| | Higher than the normal range n (%) | | | |
| | Total (Missing) | | | |
| | Statistics | | | |
| | P-value | | | |

Table 70. Summary of blood routine examination-Lymphocyte percentage (LY)

| Items | Indicator | Experimental group | Control group | Total |
|---|---|---|---|---|
| Visit 1/Day 0 | Within the normal range n (%) | | | |
| | Below the normal range n (%) | | | |
| | Higher than the normal range n (%) | | | |
| | Total (Missing) | | | |
| | Statistics | | | |
| | P-value | | | |
| Visit 9/Day 364±7 | Within the normal range n (%) | | | |
| | Below the normal range n (%) | | | |
| | Higher than the normal range n (%) | | | |
| | Total (Missing) | | | |
| | Statistics | | | |
| | P-value | | | |

Table 71. Shift-table of blood routine examination results before and after treatment-Leukocyte count (WBC)

| | | Day 364±7 after treatment | | | | | |
|---|---|---|---|---|---|---|---|
| Group | Before treatment | Normal | Abnormal but not clinically significant | Abnormal and clinically significant | Not tested | Missing | Total |
| Experimental group | Normal | | | | | | |
| | Abnormal but not clinically significant | | | | | | |
| | Abnormal and clinically significant | | | | | | |
| | Not tested | | | | | | |

| Group | Before treatment | Day 364±7 after treatment | | | | | Total |
|---|---|---|---|---|---|---|---|
| | | Normal | Abnormal but not clinically significant | Abnormal and clinically significant | Not tested | Missing | |
| | Missing | | | | | | |
| | Total | | | | | | |
| Control group | Normal | | | | | | |
| | Abnormal but not clinically significant | | | | | | |
| | Abnormal and clinically significant | | | | | | |
| | Not tested | | | | | | |
| | Missing | | | | | | |
| | Total | | | | | | |

Table 72. Shift-table of blood routine examination results before and after treatment-Erythrocyte count (RBC)
Ibid. Same below.

Table 73. Shift-table of blood routine examination results before and after treatment-Haemoglobin (Hb)

Table 74. Shift-table of blood routine examination results before and after treatment-Platelet count (PLT)

Table 75. Shift-table of blood routine examination results before and after treatment-Lymphocyte percentage (LY)

### 10.5.4. Urine routine examination

Table 76. Shift-table of urine routine examination results before and after treatment-Leukocyte
Table 77. Shift-table of urine routine examination results before and after treatment-Erythrocyte
Table 78. Shift-table of urine routine examination results before and after treatment-pH
Table 79. Shift-table of urine routine examination results before and after treatment-Urine protein
Table 80. Shift-table of urine routine examination results before and after treatment-Color
Table 81. Shift-table of urine routine examination results before and after treatment-Definition

### 10.5.5. Serum biochemistry

Table 82. Shift-table of serum biochemistry results before and after treatment-Glutamyl transpeptidase (GT)
Table 83. Shift-table of serum biochemistry results before and after treatment-Alanine aminotransferase (ALT)
Table 84. Shift-table of serum biochemistry results before and after treatment-Aspartate aminotransferase (AST)
Table 85. Shift-table of serum biochemistry results before and after treatment-Lactate dehydrogenase
Table 86. Shift-table of serum biochemistry results before and after treatment-Total bilirubin
Table 87. Shift-table of serum biochemistry results before and after treatment-Direct bilirubin
Table 88. Shift-table of serum biochemistry results before and after treatment-Alkaline phosphatase
Table 89. Shift-table of serum biochemistry results before and after treatment-Scr
Table 90. Shift-table of serum biochemistry results before and after treatment-Uric acid
Table 91. Shift-table of serum biochemistry results before and after treatment-Blood urea nitrogen
Table 92. Shift-table of serum biochemistry results before and after treatment-Cystatin C
Table 93. Shift-table of serum biochemistry results before and after treatment-Fasting blood glucose
Table 94. Shift-table of serum biochemistry results before and after treatment-Total cholesterol
Table 95. Shift-table of serum biochemistry results before and after treatment-Triglyceride
Table 96. Shift-table of serum biochemistry results before and after treatment-Total protein
Table 97. Shift-table of serum biochemistry results before and after treatment-Albumin
Table 98. Shift-table of serum biochemistry results before and after treatment-Globulin

### 10.5.6. DNA/antibody test

Table 99. Shift-table of DNA/antibody test results before and after treatment-Blood EBV-DNA
Table 100. Shift-table of DNA/antibody test results before and after treatment-IgM
Table 101. Shift-table of DNA/antibody test results before and after treatment-IgG

### 10.5.7. Urine test

Table 102. Shift-table of urine test results before and after treatment-24hr urine protein (>3 years old)
Table 103. Shift-table of urine test results before and after treatment-Urine protein/creatinine

### 10.5.8. Glomerular filtration rate

Table 104. Shift-table of eGFR results before and after treatment-eGFR

### 10.5.9. Chest X-ray

Table 105. Shift-table of chest X-ray results before and after treatment

### 10.5.10. ECG

Table 106. Shift-table of ECG examination results before and after treatment

### 10.5.11. Concomitant medication and medication adherence

Table 107. Concomitant medication

| Items | Indicator | Experimental group | Control group | Total |
|---|---|---|---|---|
| Concomitant medication | No n (%) | | | |
| | Yes n (%) | | | |
| | Total (Missing) | | | |
| | Statistics | | | |
| | P-value | | | |

Table 108. Medication adherence

| Items | Indicator | Experimental group | Control group | Total |
|---|---|---|---|---|
| Adherence to tacrolimus capsules/mycophenolate mofetil dispersible tablets (%) | N (Missing) | | | |
| | Mean (SD) | | | |
| | Median | | | |
| | Q1, Q3 | | | |
| | Min, Max | | | |
| | Statistics | | | |
| | P-value | | | |
| Adherence to tacrolimus capsules/mycophenolate mofetil dispersible tablets | Excellent (80-120%) n (%) | | | |
| | Poor (<80% or >120%) n (%) | | | |
| | Total (Missing) | | | |
| | Statistics | | | |
| | P-value | | | |
| Adherence to steroids (%) | N (Missing) | | | |
| | Mean (SD) | | | |
| | Median | | | |
| | Q1, Q3 | | | |
| | Min, Max | | | |
| | Statistics | | | |
| | P-value | | | |
| Adherence to steroids | Excellent (80-120%) n (%) | | | |
| | Poor (<80% or >120%) n (%) | | | |
| | Total (Missing) | | | |
| | Statistics | | | |
| | P-value | | | |

# 11. Attachments

Table 109. List of subjects drop-out (All randomized subjects)

| Site | Subject code | Random number | Group | Drop-out | Trial completion | Termination date | Treatment discontinuation reason | FAS | PPS | SS | Reason for not be included in the PPS |
|---|---|---|---|---|---|---|---|---|---|---|---|

Table 110. List of subjects exclusions (All randomized subjects)

| Site | Subject code | Random number | Group | Exclusion | Trial completion | Termination date | Treatment discontinuation reason | FAS | PPS | SS | Reason for not be included in the PPS |
|---|---|---|---|---|---|---|---|---|---|---|---|

Table 111. List of protocol deviations (All randomize subjects)

Note: Analysis based on external file "XX".

Table 112. List of demographic characteristics (FAS)

| Site | Subject code | Random number | Group | Date of birth | Age (years) | Gender | Ethnicity | Other ethnicity | Source of disease | Height (cm) | Weight (kg) | BMI (kg/$m^2$) |
|---|---|---|---|---|---|---|---|---|---|---|---|---|

Table 113. List of family history and personal history (FAS)

| Site | Subject code | Random number | Group | Family history | History of drug allergy | History of surgery | History of pet ownership | Number of gestation | Number of parturition | Birth weight (kg) | History of dystocia | Feeding history | Partial eclipse | History of growth and development | Vaccination history |
|---|---|---|---|---|---|---|---|---|---|---|---|---|---|---|---|

Table 114. List of history of other diseases and concomitant medications (FAS)

| Site | Subject code | Random number | Group | Disease name | Start date | End date | Disease persists or not | Drug name | Drug dosage form | Dosage | Frequency | Still on medication or not |
|---|---|---|---|---|---|---|---|---|---|---|---|---|
| | | | | | | | | | | | | |

Table 115. List of history of nephrotic syndrome (FAS)

| Site | Subject code | Random number | Group | Onset date | Diagnosis date | Course of disease | No. of relapses in the past 1 year | No. of relapses in the past 6 months | Time to recurrence | Negative conversion time of urine protein |
|---|---|---|---|---|---|---|---|---|---|---|
| | | | | | | | | | | |

Table 116. List of treatment history for nephrotic syndrome (FAS)

| Site | Subject code | Random number | Group | Drug name | Drug dosage form | Dosage | Frequency of medication | Start medication date | Stop medication date | Still on medication or not |
|---|---|---|---|---|---|---|---|---|---|---|
| | | | | | | | | | | |

Table 117. List of disease relapse (urine protein) situations (FAS)

| Site | Subject code | Random number | Group | Visit | Relapse or not | Serial number | Relapse start date | Relapse end date | Description of relapse |
|---|---|---|---|---|---|---|---|---|---|
| | | | | | | | | | |

Table 118. List of primary efficacy indicator (1-year relapse-free) situations (FAS/PPS)

| Site | Subject code | Random number | Group | Relapse or not | Relapse start date | Relapse end date | Random date | Analyze date | Endpoint event |
|---|---|---|---|---|---|---|---|---|---|
| | | | | | | | | | |

Table 119. List of secondary efficacy indicator (first relapse after enrollment) situations (FAS/PPS)

| Site | Subject code | Random number | Group | Relapse or not | Relapse start date | Relapse end date | Random date | Analyze date | Endpoint event |
|---|---|---|---|---|---|---|---|---|---|

Table 120. List of secondary efficacy indicator (6-month relapse-free) situations (FAS/PPS)

| Site | Subject code | Random number | Group | Relapse or not | Relapse start date | Relapse end date | Random date | Analyze date | Endpoint event |
|---|---|---|---|---|---|---|---|---|---|

Table 121. List of blood routine examination results that were normal before treatment and abnormal after treatment (SS)

| Site | Subject code | Random number | Group | Examination items | Visit | Baseline result | Baseline unit | Baseline clinical judgment | Visit result | Visit unit | Visit clinical judgment |
|---|---|---|---|---|---|---|---|---|---|---|---|

Table 122. List of urine routine examination results that were normal before treatment and abnormal after treatment (SS)

| Site | Subject code | Random number | Group | Examination items | Visit | Baseline result | Baseline unit | Baseline clinical judgment | Visit result | Visit unit | Visit clinical judgment |
|---|---|---|---|---|---|---|---|---|---|---|---|

Table 123. List of serum biochemistry results that were normal before treatment and abnormal after treatment (SS)

| Site | Subject code | Random number | Group | Examination items | Visit | Baseline result | Baseline unit | Baseline clinical judgment | Visit result | Visit unit | Visit clinical judgment |
|---|---|---|---|---|---|---|---|---|---|---|---|

Table 124. List of urine test results that were normal before treatment and abnormal after treatment (SS)

| Site | Subject code | Random number | Group | Examination items | Visit | Baseline result | Baseline unit | Baseline clinical judgment | Visit result | Visit unit | Visit clinical judgment |
|---|---|---|---|---|---|---|---|---|---|---|---|

Table 125. List of eGFR results that were normal before treatment and abnormal after treatment (SS)

| Site | Subject code | Random number | Group | Visit | Baseline result | Baseline clinical judgment | Visit result | Visit clinical judgment |
|---|---|---|---|---|---|---|---|---|

Table 126. List of chest X-ray results that were normal before treatment and abnormal after treatment (SS)

| Site | Subject code | Random number | Group | Visit | Baseline result | Baseline clinical judgment | Visit result | Visit clinical judgment | Description of abnormal |
|---|---|---|---|---|---|---|---|---|---|

Table 127. List of ECG examination results that were normal before treatment and abnormal after treatment (SS)

| Site | Subject code | Random number | Group | Visit | Baseline result | Baseline clinical judgment | Visit result | Visit clinical judgment | Description of abnormal |
|---|---|---|---|---|---|---|---|---|---|

Table 128. List of AE (SS)

| Site | Subject code | Random number | Group | AE ID | Description of AE | Severity | Start date | Treatment or not | AE ended or not | End date | Treatment | Relationships with drug | Measures taken on study drug |
|---|---|---|---|---|---|---|---|---|---|---|---|---|---|
| | | | | | | | | | | | | | |

Table 129. List of AE-continued (SS)

| Site | Subject code | Random number | Group | AE ID | Description of AE | AE outcomes | Recovery/cure date | Details of sequelae | Death date | SAE or not | Cause withdraw or not |
|---|---|---|---|---|---|---|---|---|---|---|---|
| | | | | | | | | | | | |

Table 130. List of SAE (SS)

| Site | Subject code | Random number | Group | SAE ID | Type of SAE | Report date | Description of SAE | SAE situations | Death date | Severity | Onset Date | SAE outcomes | Relationships with drug |
|---|---|---|---|---|---|---|---|---|---|---|---|---|---|
| | | | | | | | | | | | | | |

Table 131. List of SAE-continued (SS)

| Site | Subject code | Random number | Group | SAE ID | Type of SAE | Report time | Description of SAE | Measures taken on study drug | Reported SAE situation | Details of SAE cccurrence and treatment |
|---|---|---|---|---|---|---|---|---|---|---|
| | | | | | | | | | | |

Table 132. List of concomitant medication (SS)

| Site | Subject code | Random number | Group | CM ID | China approved drug names | Administration route | Single dose | Unit | Frequency | Purpose of medication | Dosing start date | Still in use | Dosing end date |
|---|---|---|---|---|---|---|---|---|---|---|---|---|---|
| | | | | | | | | | | | | | |
