# Supplementary material for: Tacrolimus or Mycophenolate Mofetil for Frequently Relapsing or Steroid-Dependent Nephrotic Syndrome: A Randomized Clinical Trial
Source: JAMA Pediatr. 2025 May 12;179(7):722–9. doi: 10.1001/jamapediatrics.2025.0765 (PMC12070277; doi:10.1001/jamapediatrics.2025.0765)
Supplement: Supplement 3. — eTable 1. Reasons for Active Withdrawal eTable 2. Baseline Characteristics of the Retained Participants eTable 3. 1-Year Relapse-Free Survival Using Cox Regression (FAS and PPS) eTable 4. Adverse Events eTable 5. Comparison of eGFRs Estimated via Various Formulas and Assessment of Differences in Renal Function Between the TAC and MMF Groups via the MMRM eFigure. Subgroup Analysis of Relapses Within 1-year eMethods 1. Pharmacokinetic Studies eMethods 2. Statistical Analysis eReferences. [file jamapediatr-e250765-s003.pdf]

# Supplemental Online Content

Wang J, Liu F, Yan W, et al. Tacrolimus or mycophenolate mofetil for frequently relapsing or steroid-dependent nephrotic syndrome: a randomized clinical trial. *JAMA Pediatr*. Published online May 12, 2025. doi:10.1001/jamapediatrics.2025.0765

**eTable 1.** Reasons for Active Withdrawal
**eTable 2.** Baseline Characteristics of the Retained Participants
**eTable 3.** 1-Year Relapse-Free Survival Using Cox Regression (FAS and PPS)
**eTable 4.** Adverse Events
**eTable 5.** Comparison of eGFRs Estimated via Various Formulas and Assessment of Differences in Renal Function Between the TAC and MMF Groups via the MMRM
**eFigure.** Subgroup Analysis of Relapses Within 1-year
**eMethods 1.** Pharmacokinetic Studies
**eMethods 2.** Statistical Analysis
**eReferences.**

This supplemental material has been provided by the authors to give readers additional information about their work.

© 2025 Wang J et al. *JAMA Pediatrics*.

**eTable 1.** Reasons for Active Withdrawal

| Group | No. | Reason |
|---|---|---|
| TAC Group | 1037 | Parents wanted to give the subject traditional Chinese medicine. |
| MMF Group | 2010 | The subject experienced several relapses in nine months. |
| | 9006 | The subject experienced two relapses and was hospitalized for treatment. |
| | 9030 | The subject experienced two relapses and was hospitalized for treatment. |
| | 1011 | The subject experienced severe gastrointestinal reactions. |
| | 5017 | The subject was unable to visit on time. |
| | 11010 | The subject experienced one relapse in a short period of time. |
| | 11014 | The subject experienced one relapse in a short period of time. |
| | 1007 | The subject experienced several relapses. |
| | 1033 | The subject experienced several relapses |
| | 5044 | The subject experienced several relapses |

NOTE: 1 patient in the TAC arm and 10 patients in the MMF arm

© 2025 Wang J et al. *JAMA Pediatrics*.

**eTable 2.** Baseline Characteristics of the Retained Participants

| **Characteristic** | **TAC group (n=129)** | **MMF group (n=114)** | ***P* value** |
|---|---|---|---|
| Age of enrollment, median (range), year | 6.86 (2.16-16.43) | 7.29 (1.95-16.53) | 0.66 |
| Male sex, No. (%) | 96(74.42%) | 83 (72.81%) | 0.78 |
| Height, mean ± SD, cm | 118.56±20.71 | 121.24±21.37 | 0.43 |
| Weight, mean ± SD, kg | 27.06±12.34 | 29.34±15.74 | 0.52 |
| BMI, mean ± SD, $kg/m^2$ | 18.36±3.64 | 18.63±3.67 | 0.52 |
| Systolic blood pressure, mean ± SD, mmHg | 105.00±11.22 | 105.54 ±10.52 | 0.70 |
| Diastolic blood pressure, mean ± SD, mmHg | 65.67±9.78 | 66.09±9.62 | 0.69 |
| eGFR*, mean ± SD, $ml/min/1.73m^2$ | 164.55±35.86 | 164.79±38.79 | 0.96 |
| Serum creatinine, mean ± SD, µmol/L | 36.00±10.49 | 37.03±12.05 | 0.58 |
| Total cholesterol, mean ± SD, mmol/L | 6.57±2.32 | 6.29±1.91 | 0.51 |
| Albumin, mean ± SD, g/L | 34.26±7.18 | 34.84±7.19 | 0.83 |
| Total protein, mean ± SD, g/L | 58.13±8.02 | 58.38±8.36 | 0.81 |
| Fasting blood glucose, mean ± SD, mmol/L | 4.34±0.72 | 4.44±0.82 | 0.34 |
| Duration of disease, Median (IQR), year | 1.40 (0.75, 3.00) | 1.18 (0.75, 2.50) | 0.53 |
| No. of relapses in the prestudy half year, mean ± SD, times | 2.15 ±0.74 | 2.28 ±1.11 | 0.44 |

NOTE: Values are means ± SDs, medians (ranges) or medians (IQRs). TAC, tacrolimus; MMF, mycophenolate mofetil; BMI, body mass index (calculated as weight in kilograms divided by height in meters squared).
*eGFR, estimated glomerular filtration rate (calculated by the Schwartz formula[1])

© 2025 Wang J et al. *JAMA Pediatrics*.

**eTable 3.** 1-Year Relapse-Free Survival Using Cox Regression (FAS and PPS)

| | | FAS | | PPS | |
|---|---|---|---|---|---|
| Item | Index | TAC group | MMF group | TAC group | MMF group |
| Survival time | N (missing) | 135 (0) | 135 (0) | 131 (0) | 127 (0) |
| | No. event* | 24 | 56 | 24 | 53 |
| | Logrank test | 20.21 | | 18.34 | |
| | P value | <0.001 | | <0.001 | |
| Survival time Cox regression(1) | Hazard ratio | 0.35 | | 0.36 | |
| | 95% CI | 0.21, 0.56 | | 0.22, 0.58 | |
| | Wald chi square | 18.84 | | 17.25 | |
| | P value | <0.001 | | <0.001 | |
| Survival time Cox regression [adjusted](2) | Hazard ratio | 0.35 | | 0.36 | |
| | 95% CI | 0.22, 0.57 | | 0.22, 0.59 | |
| | Wald chi square | 17.80 | | 16.53 | |
| | P value | <0.001 | | <0.001 | |
| Survival time Cox regression [adjusted](3) | Hazard ratio | 0.35 | | 0.36 | |
| | 95% CI | 0.22, 0.57 | | 0.22, 0.59 | |
| | Wald chi square | 17.67 | | 16.37 | |
| | P value | <0.001 | | <0.001 | |

*Event is defined as relapse.

Cox regression (1): the independent variables are group and center.

Cox regression [adjusted] (2), adjusted factors: age at enrollment, sex, age at onset, duration of kidney disease.

Cox regression [adjusted] (3), adjusted factors: center, age at enrollment, sex, age at onset, duration of kidney disease.

© 2025 Wang J et al. *JAMA Pediatrics*.

**eTable 4.** Adverse Events

| Event | TAC group (n=135) | MMF group (n=135) | *P* Value |
|---|---|---|---|
| Any adverse event (AE), n (%) | 128 (94.81) | 125 (92.59) | 0.62 |
| AEs associated with the study medication | 46 (34.07) | 49 (36.30) | 0.80 |
| Serious adverse events (SAE) | 12 (8.89) | 14 (10.37) | 0.84 |
| SAEs associated with the study medication | 5 (3.70) | 7 (5.19) | 0.77 |
| Infectious and invasive diseases | 3 (2.22) | 4 (2.96) | |
| Pneumonia | 2 (1.48) | 1 (0.74) | |
| Chronic tonsillitis | 0 | 1 (0.74) | |
| Bacterial tonsillitis | 1 (0.74) | 0 | |
| Upper respiratory tract infections | 0 | 2 (1.48) | |
| Diseases of the endocrine system | 0 | 1 (0.74) | |
| Adrenal insufficiency | 0 | 1 (0.74) | |
| Metabolic and nutritional diseases | 1 (0.74) | 0 | |
| Hyperglycemia | 1 (0.74) | 0 | |
| Lesions, poisonings, and complications of procedures | 0 | 1 (0.74) | |
| Superficial erosion of the lower extremity | 0 | 1 (0.74) | |
| Diseases of the skin and subcutaneous tissue | 1 (0.74) | 0 | |
| Cystic acne | 1 (0.74) | 0 | |
| Diseases of the blood and lymphatic system | 0 | 1 (0.74) | |
| Lymphadenitis | 0 | 1 (0.74) | |
| AEs leading to study withdrawal | 1 (0.74) | 1 (0.74) | 1.00 |
| SAEs leading to study withdrawal | 1 (0.74) | 0 | 1.00 |

*Data expressed as number of patients (percent).*

© 2025 Wang J et al. *JAMA Pediatrics*.

**eTable 5.** Comparison of eGFRs Estimated via Various Formulas and Assessment of Differences in Renal Function Between the TAC and MMF Groups via the MMRM

| End Point | TAC group (n=135) | MMF group (n=135) | *P* value |
|---|---|---|---|
| eGFR value from baseline calculated by the Schwartz formula[1] | 164.81±35.71 | 165.54±39.46 | 0.87 |
| eGFR value from last visit calculated by the Schwartz formula | 158.43±34.26 | 163.40±33.19 | 0.26 |
| Change in eGFR from baseline to last visit calculated by the Schwartz formula | -7.71±31.67 | -1.56±31.18 | 0.14 |
| eGFR value from baseline calculated by the updated Schwartz formula[2] | 106.59±15.96 | 107.99±17.82 | 0.50 |
| eGFR value from last visit calculated by the updated Schwartz formula | 106.09±16.26 | 113.42±18.76 | 0.002 |
| Change in eGFR from baseline to last visit calculated by the updated Schwartz formula | -0.69±19.11 | 6.44±17.47 | 0.004 |
| eGFR value from baseline calculated by the equation K × [heigh(ht)/serum creatinine(sCr)], (used a sex-dependent K)[3] | 123.84±24.24 | 126.15±29.73 | 0.49 |
| eGFR value from last visit calculated by the equation K × [heigh(ht)/serum creatinine(sCr)], (used a sex-dependent K) | 120.65±24.65 | 124.82±23.34 | 0.12 |
| Change in eGFR from baseline to last visit calculated by the equation K × [heigh(ht)/serum creatinine(sCr)], (used a sex-dependent K) | -4.00±27.51 | -0.12±25.20 | 0.26 |
| eGFR value from baseline calculated by the equation K × [heigh(ht)/serum creatinine(sCr)], (used a sex-and age-dependent K)[3] | 112.73±21.80 | 114.68±26.88 | 0.51 |
| eGFR value from last visit calculated by the equation K × [heigh(ht)/serum creatinine(sCr)], (used a sex-and age-dependent K) | 109.71±21.77 | 113.64±21.02 | 0.08 |
| Change in eGFR from baseline to last visit calculated by the equation K × [heigh(ht)/serum creatinine(sCr)], (used a sex-and age-dependent K) | -3.70±24.68 | -0.18±23.09 | 0.26 |
| eGFR value from baseline calculated by the equation K × [1/cystatin C(cysC)], (used a sex-dependent K)[3] | 97.30±24.49 | 99.32±21.40 | 0.42 |
| eGFR value from last visit calculated by the equation K × [1/cystatin C(cysC)], (used a sex-dependent K) | 94.95±20.58 | 101.48±23.49 | 0.05 |
| Change in eGFR from baseline to last visit calculated by the equation K × [1/cystatin C(cysC)], (used a sex-dependent K) | -2.04±26.62 | 3.54±22.50 | 0.12 |
| eGFR value from baseline calculated by the equation K × [1/cystatin C(cysC)], (used a sex-and age-dependent K)[3] | 96.89±25.03 | 98.37±20.67 | 0.51 |

© 2025 Wang J et al. *JAMA Pediatrics*.

| | | | |
|---|---|---|---|
| eGFR value from last visit calculated by the equation K × [1/cystatin C(cysC)], (used a sex-and age-dependent K) | 94.47±20.29 | 100.68±22.79 | 0.05 |
| delta eGFR from baseline to last visit calculated by the equation K × [1/cystatin C(cysC)], (used a sex-and age-dependent K) | -2.17±26.72 | 3.55±22.15 | 0.11 |
| Least-squares mean change from baseline in eGFR at month 12 - ml/min/1.73 $m^2$ | -12.28±1.57 | -5.14±1.60 | |
| Least-squares mean change from baseline in serum creatine at month 12 - (μmol/L) | 3.49±0.48 | 1.10±0.49 | |
| Least-squares mean change from baseline in serum urea nitrogen at month 12 - (mmol/L) | 0.42±0.07 | -0.18±0.07 | |
| Least-squares mean change from baseline in Serum cystatin C at month 12 - (mg/L) | 0.05±0.01 | 0.00±0.01 | |

**Reference**


1. Schwartz GJ, Brion LP, Spitzer A. THE USE OF PLASMA CREATININE CONCENTRATION FOR ESTIMATING GLOMERULAR-FILTRATION RATE IN INFANTS, CHILDREN, AND ADOLESCENTS. *Pediatric Clinics of North America* 1987; **34**(3): 571-90.
2. Schwartz GJ, Munoz A, Schneider MF, et al. New Equations to Estimate GFR in Children with CKD. *Journal of the American Society of Nephrology* 2009; **20**(3): 629-37.
3. Pierce CB, Munoz A, Ng DK, Warady BA, Furth SL, Schwartz GJ. Age- and sex-dependent clinical equations to estimate glomerular filtration rates in children and young adults with chronic kidney disease. *Kidney International* 2021; **99**(4): 948-56.


© 2025 Wang J et al. *JAMA Pediatrics*.

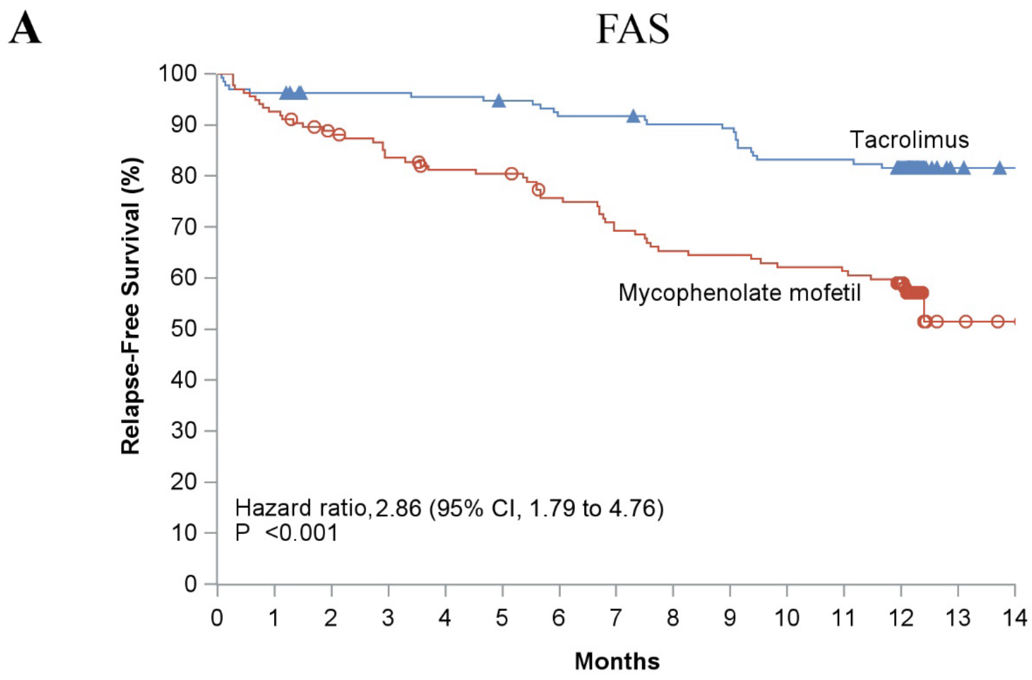


| No. at Risk | | | | | | | | | | | | | | |
|---|---|---|---|---|---|---|---|---|---|---|---|---|---|---|
| Tacrolimus | 135 | 130 | 126 | 126 | 125 | 123 | 119 | 119 | 116 | 115 | 107 | 107 | 99 | 3 | 1 |
| Mycophenolate mofetil | 135 | 125 | 117 | 109 | 104 | 103 | 95 | 87 | 82 | 81 | 78 | 77 | 70 | 3 | 1 |

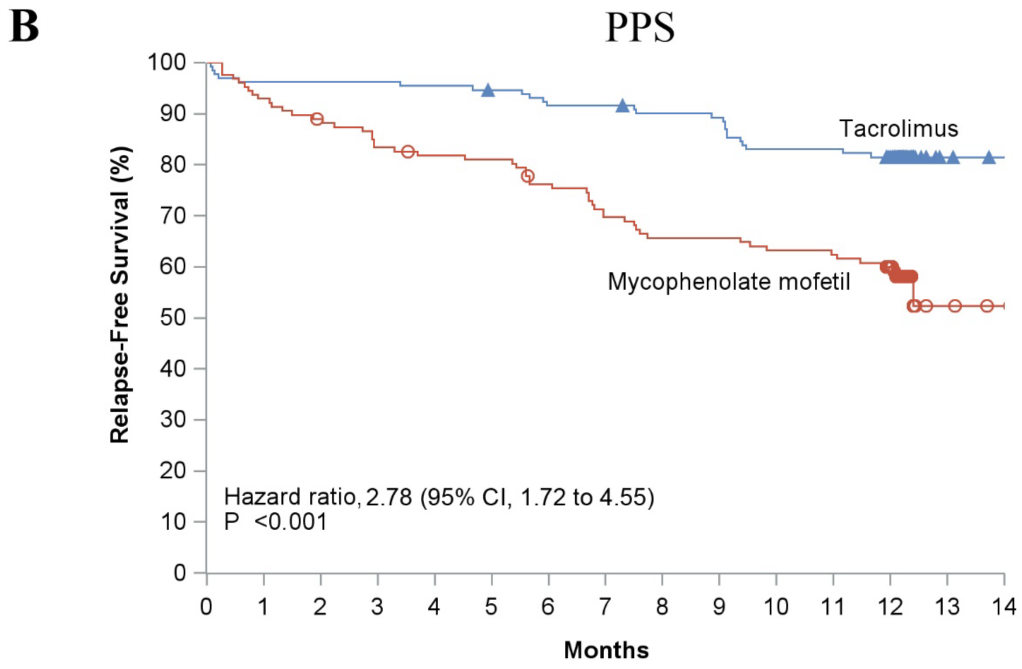


| No. at Risk | | | | | | | | | | | | | | |
|---|---|---|---|---|---|---|---|---|---|---|---|---|---|---|
| Tacrolimus | 131 | 126 | 126 | 126 | 125 | 123 | 119 | 119 | 116 | 115 | 107 | 107 | 99 | 3 | 1 |
| Mycophenolate mofetil | 127 | 118 | 112 | 105 | 102 | 101 | 94 | 86 | 81 | 81 | 78 | 77 | 70 | 3 | 1 |

**eFigure.** Subgroup Analysis of Relapses Within 1-year

© 2025 Wang J et al. *JAMA Pediatrics*.

**eMethods 1.** Pharmacokinetic Studies

Enzyme-linked immunosorbent assays (ELISA) were used to measure plasma TAC trough levels according to the manufacturer's instructions. Plasma levels of TAC were measured at visit 2th (1 week after receiving TAC treatment). Plasma TAC levels were regularly checked (visit 2th, 3th, 4th, 5th, 6th, 7th, 9th) for all TAC group patients.

A 3-point abbreviated pharmacokinetic profile for MPA was performed at visit 2th and visit 6th based on MPA plasma concentrations before oral intake of MMF (C0), 30 min (C0.5) and 2 hours (C2) thereafter[1]. The area under the time-concentration curve (AUC) was calculated as follows:

MPA -AUC = 7.75 + (6.49*C0) + (0.76*C0.5) + (2.43*C2)[1-3].

The MPA pharmacokinetic profile was analyzed in a central laboratory (Department of Clinical Pharmacology, Hangzhou Leading Pharmatech Co. Ltd). MPA was measured by ultra-performance liquid chromatography-tandem mass spectrometry method.

© 2025 Wang J et al. *JAMA Pediatrics*.

**eMethods 2.** Statistical Analysis

The sample size calculation is based on the primary hypothesis. On the basis of previous studies(ref), we assume a 12-month relapse-free survival rate of 0.56 for the MMF group and 0.70 for the TAC group. The enrollment period was expected to last two years, with a total study duration of three years. Using the log-rank test module in PASS software with the proportion survival model to compare the survival rates, 229 patents based on a 1:1 ratio of allocation are required to detect a hazard ratio of 0.8 (0.56/0.70) at a two-sided type I error of 0.05 to ensure a power of 80%. Considering a loss to follow-up rate of 15% (without considering screening), the total sample size of 229 patients was inflated to 270, with 135 patients in each group.

The primary analysis of the primary outcome will be based on the intention-to-treat (ITT) principle using the full analysis set (FAS).

The endpoint event is defined as a relapse with the following calculation rules:

1. If a relapse occurred within a visit of 9/364±7 days or during an unplanned visit within 365 days, the endpoint event was defined as "relapse," with the following calculation rules: days after randomization, it was defined as an endpoint event. The time to relapse was calculated as the date of relapse minus the date of randomization plus one (day).
2. If, within visit 9/day 364±7, the visit as specified in the plan was not completed due to loss to follow-up, withdrawal, or other reasons, it was censored. The time censored is calculated as the date of withdrawal minus the date of randomization plus one, calculated in days.
3. If, within visit 9/day 364±7, the visit as specified in the plan was not completed because of death during the trial, it was censored. The censored time was calculated as the date of death minus the date of randomization plus one, calculated in days.
4. If a visit of 9/7 on day 364±7 was completed as specified in the plan and there was no relapse, the patient was censored. The time censored was calculated as the date of visit 9/day 364±7 minus the date of randomization plus one, calculated in days.

The time to relapse was defined according to the following rules:

Relapse Event: If relapse occurred during Visit 9 (Day 364 ± 7 days) or within 365 days, the time to relapse = relapse date - randomization date + 1 day.

Censoring for Loss to Follow-up/Withdrawal: If Visit 9 is missed due to loss to follow-up or withdrawal, the censored time = withdrawal date - randomization date + 1 day.

Censoring for death: If Visit 9 is missed due to death, censored time = death date -

© 2025 Wang J et al. *JAMA Pediatrics*.

randomization date + 1 day.

Censoring for No Relapse: If Visit 9 is completed without relapse, censored time = Visit 9 date - randomization date + 1 day.

The P value for the primary analysis for differences in relapse-free survival rates between the two groups was obtained from the log-rank test to draw conclusions, and a univariate Cox proportional hazards model was used to obtain the hazard ratio (HR) and 95% confidence interval (CI) as estimates of the treatment effect after assessment of the proportional hazards assumption. The confidence interval is used to determine the superiority of the experimental group over the control group, with a boundary value of 1 (hazard ratio). Kaplan–Meier curves of relapse-free survival rates over 12 months in the two groups were plotted. Sensitivity analysis of the primary outcome was conducted on the basis of the PPS dataset by replicating the Cox model with adjustments for patient age, age of onset, sex, duration of disease at baseline as covariates, and study center. Furthermore, subgroup analyses were conducted via the same model according to the categorized baseline characteristics, including age (<1 yr, ≥1 yr), age at onset (<1 yr, ≥1 yr), sex, and disease duration (<1 yr, ≥1 yr) in the FAS and PPS populations.

A mixed model with repeated measurements (MMRM) was employed to compare the change in eGFR from baseline between the two treatment groups. The model includes the fixed effects of treatment group, categorical visit (12 monthly visits), and a treatment-by-visit interaction, with a fixed effect covariate of baseline eGFR, a random effect of intercept and visit, and an unstructured covariance matrix. From the MMRM, the following statistics will be produced: 1) the least-square mean (LSM) and standard error (SE) and 95% CI for each treatment group and 2) the least-square mean difference (LSMD) between treatment groups using placebo as a reference, its corresponding SE and 95% CI and p value for the LSMD test.

Analysis methods for secondary outcomes

The analysis utilized a mixed model with repeated measurements (MMRM) to compare the change in eGFR from baseline between the two treatment groups. The model included fixed effects for treatment group, visit (12 monthly visits), and treatment-by-visit interaction, with baseline eGFR as a covariate. The random effects included intercept and visit, and an unstructured covariance matrix was used. The following outputs were generated: 1) least-square means (LSM), standard errors (SE), and 95% CIs for each treatment group; and 2) LSM differences (LSMD) between treatment groups (placebo as reference), along with corresponding SE, 95% CI, and p values for the LSMD test.

The annualized slope of the eGFR over 12 months between the two groups was tested via a

© 2025 Wang J et al. *JAMA Pediatrics*.

linear mixed effects model with random effects. The model was fitted with fixed effects of treatment group, categorical visit (12 monthly visits), treatment-by-visit interaction, baseline eGFR and random effects of intercept and time and with the assumption of an unstructured covariance structure. The annualized eGFR slope for each treatment group, SE and 95% CI are reported. Blood creatinine, BMI, cystatin C, and uric acid were analyzed via the same methods described above for eGFR. The missing data were assumed at random for the above analyses, and all reported 95% CIs were not adjusted for multiplicity.

The annualized slope of the eGFR over 12 months was compared between the two treatment groups via a linear mixed-effects model. The model included fixed effects for treatment group, categorical visit (12 monthly visits), treatment-by-visit interaction, and baseline eGFR, as well as random effects for intercept and time. An unstructured covariance structure was assumed. The annualized eGFR slope in each group and the mean difference between groups, standard error (SE), and 95% confidence interval (CI) are reported.

Blood creatinine, BMI, cystatin C, and uric acid were analyzed via the same approach as the primary outcome.

Missing data were assumed to be missing at random, and all reported 95% CIs were unadjusted for multiplicity.

Safety outcomes were analyzed via the safety analysis set. Adverse events (AEs) (including serious adverse events) were coded with MedDRA 26.0 and summarized as cumulative rates and incidence rates. Comparisons between groups were made via the chi-square test or Fisher's exact test, with P values reported only.

© 2025 Wang J et al. *JAMA Pediatrics*.

**eReferences.**

1. Pawinski T, Kunicki PK, Sobieszczanska-Malek M, Gralak B, Szlaska I. A limited sampling strategy for estimating mycophenolic acid area under the curve in adult heart transplant patients treated with concomitant cyclosporine. J Clin Pharm Ther 2009; 34: 89-101.

2. Gellermann J, Weber L, Pape L, Tönshoff B, Hoyer P, Querfeld U. Mycophenolate mofetil versus cyclosporin A in children with frequently relapsing nephrotic syndrome. J Am Soc Nephrol 2013;24:1689-97.

3. Trautmann A, Boyer O, Hodson E, et al. IPNA clinical practice recommendations for the diagnosis and management of children with steroid-sensitive nephrotic syndrome. Pediatric Nephrology (Berlin, Germany) 2023;38:877-919.

© 2025 Wang J et al. *JAMA Pediatrics*.
