# Supplementary material for: Tacrolimus or Mycophenolate Mofetil for Frequently Relapsing or Steroid-Dependent Nephrotic Syndrome: A Randomized Clinical Trial
Source: JAMA Pediatr. 2025 May 12;179(7):722–9. doi: 10.1001/jamapediatrics.2025.0765 (PMC12070277; doi:10.1001/jamapediatrics.2025.0765)
Supplement: Supplement 4. — Data Sharing Statement [file jamapediatr-e250765-s004.pdf]

Data Sharing Statement

Wang. Tacrolimus or Mycophenolate Mofetil for Frequently Relapsing or Steroid-Dependent Nephrotic Syndrome. *JAMA Pediatr*. Published May 12, 2025.
doi:10.1001/jamapediatrics.2025.0765

Data
Additional Information: Clinical Trials Register: NCT04048161
https://clinicaltrials.gov/study/NCT04048161?titles=STAMP&page=3&rank=30#more-information
Data available: No

Additional Information
Explanation for why data not available: The data supporting the results of the present study are available from the corresponding author upon reasonable request.
